# Development of a Nucleoside Photosensitizer Efficiently Activated by One- or Two-Photon Absorption in the Optical Therapeutic Window

**DOI:** 10.1021/jacs.6c00216

**Published:** 2026-06-08

**Authors:** Sourav Kanti Seth, Sean J. Hoehn, Chris Acquah, Liraz Levi, Carlos E. Crespo-Hernández

**Affiliations:** 1 Department of Chemistry, 2546 Case Western Reserve University, Cleveland, Ohio 44106, United States; 2 Department of Pediatrics, Case Western Reserve University School of Medicine, Cleveland, Ohio 44106, United States; 3 Celloram Inc., Cleveland, Ohio 44106, United States

## Abstract

Developing DNA/RNA nucleoside prodrugs that absorb in the red-to-near-infrared is crucial for applications requiring deep-tissue penetration. This study reports the rational design and synthesis of a novel thiocarbonyl analog, 5-(5-(4-(dimethylamino)­phenyl)­thiophen-2-yl)-2′,3′,5′-tri-*O*-benzoyl-6-aza-2,4-dithiouridine (DATU), efficiently activated through one- and two-photon absorption in the red-to-near-infrared spectrum. We report DATU’s electronic structure and its photophysical and triplet-state properties under both excitation, comparing these results to its carbonyl counterpart, 5-(5-(4-(dimethylamino)­phenyl)­thiophen-2-yl)-2’,3′,5′-tri-*O*-benzoyl-6-azauridine (DAU). DATU exhibits strong visible one-photon absorption, with ε_522_ = (0.73 ± 0.02) × 10^4^ M^–1^ cm^–1^ in aqueous solution, extending to ∼750 nm. Additionally, it features a triplet-state lifetime of 1.7 ± 0.1 μs and a singlet-oxygen quantum yield of 56% in benzene. Photoexcited DATU generates both singlet oxygen and hydroxyl radicals in solvents of varying polarity (water, methanol, acetonitrile, and benzene), functioning as both a type I and type II photosensitizer depending on the cellular environment. Notably, replacing carbonyl with thiocarbonyl groups increases the two-photon absorption cross section at 800 nm 3-fold, from 55 ± 5 GM for DAU to 160 ± 20 GM for DATU, the largest reported for a nucleoside analog. *In vitro* investigations using 4T1 murine mammary carcinoma cells confirm that DATU exhibits significant photocytotoxicity under one-photon (525 nm, 6 μM) and two-photon (800 nm, 25 μM) activation, with negligible dark toxicity. Together, these findings position DATU as the most promising nucleoside photosensitizer for deep-tissue photodynamic therapy (PDT) to date, laying the foundation for future mechanistic/*in vivo* studies while broadly guiding the development of next-generation thiocarbonyl photosensitizers for PDT, bioimaging, and photocatalysis.

## Graphical abstract

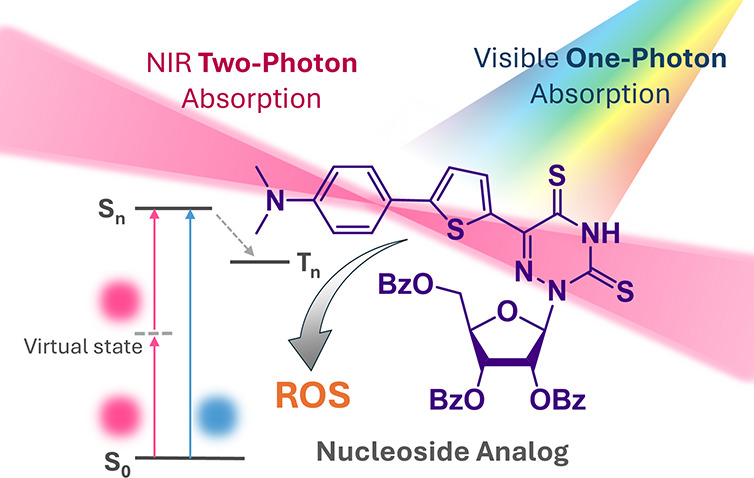

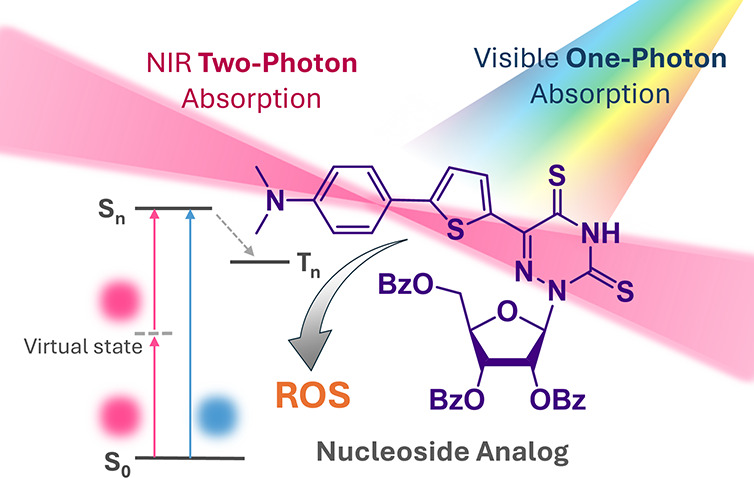

## Introduction

Sulfur-substituted nucleobases have a rich history in clinical medicine, spanning over 50 years, as agents for cancer treatment, anti-inflammatory purposes, and immunosuppression.^1,2^ Notable examples of these prodrugs include azathioprine, 6-mercaptopurine (6-MP), and 6-thioguanine (6-TG),^1–3^ with azathioprine and 6-MP still listed as essential medicines by the World Health Organization.^4^ These prodrugs are enzymatically converted into 6-thioguanine nucleotides within cells, which then incorporate into DNA and disrupt the replication process in rapidly dividing cells.^1,5^ For their groundbreaking work in uncovering the biochemical mechanisms behind these agents and promoting rational drug development, Gertrude B. Elion and George H. Hitchings were awarded the Nobel Prize in Physiology or Medicine in 1988.^6^

Since the early 2010s, sulfur-substituted nucleobases, also known as thiobases, have gained increased attention as promising candidates for photodynamic therapy (PDT). This interest is primarily due to their intrinsic photoreactivity and potential in cancer treatment.^3,7–9^ Extensive studies conducted by our research group^3,7,10–12^ and others^13,14^ have focused on their phototherapeutic potential. These studies have also demonstrated that thionation red-shifts the absorption spectra, increases the intersystem crossing (ISC) efficiency to populate long-lived triplet states, increases the photoreactivity, and generates high amounts of singlet oxygen (^1^O_2_). Furthermore, these studies indicate that thiobase derivatives are effective PDT agents in both normoxic and hypoxic conditions.^9,15,16^ Under hypoxic conditions, the long-lived triplet state reacts with DNA through photocycloaddition^15^ and photo-cross-linking^16^ reactions. Indeed, the dual functionality of the thiobases—DNA metabolization and light-triggered phototoxicity—has sparked significant interest as dual-mode anticancer agents.^7,16–21^ However, their characteristic absorption in the UVA region and their small two-photon absorption cross sections (2PACS) in the near-infrared have limited their clinical use primarily to topical skin cancer applications.^1,8,22–25^ Therefore, an essential goal in advancing thiobase-based prodrugs as PDT agents for deep-tissue treatment is to extend their absorption into the optical therapeutic window (∼650–950 nm).^26^ This extension would not only allow for deeper tissue penetration but also reduce off-target damage, which is necessary for effectively treating deep-seated tumors. Yet, efforts to design thiobase prodrug analogs absorbing one or two photons in the therapeutic window have remained elusive during the past two decades until now.^3,9,12^

In this study, we present the synthesis of a rationally designed thiobase analog of a 6-azauridine derivative, specifically 5-(5-(4-(dimethylamino)­phenyl)­thiophen-2-yl)-2′,3′,5′-tri-*O*-benzoyl-6-aza-2,4-dithiouridine (referred to as DATU, see Scheme 1). DATU was designed as a potential prodrug based on the precedent of its parent nucleoside, 2′,3′,5′-tri-*O*-acetyl-6-azauridine (azaribine), which has been clinically used for the treatment of psoriasis.^27–29^ In azaribine, the acetyl-protected ribose enhances lipophilicity and cell membrane penetration, after which intracellular enzymes remove the acetyl groups to release the active nucleoside, 6-azauridine. By analogy, DATU introduces a benzoyl-protected sugar moiety to similarly increase membrane permeability and promote cellular internalization. Although benzoyl groups are generally more stable than acetyl groups and less frequently explored in biological systems,^30^ literature reports indicate that benzoyl and benzoyl-derived esters can undergo enzymatic removal by DNA polymerases and cellular esterases in related sugar-based systems,^30–33^ suggesting that they are not inherently resistant to intracellular hydrolysis. Collectively, these precedents support the design rationale that DATU may undergo intracellular enzymatic removal of its benzoyl groups to generate the free nucleoside and subsequently act as a metabolically activated nucleoside analog, although direct biological validation remains to be established.

**Scheme 1:**
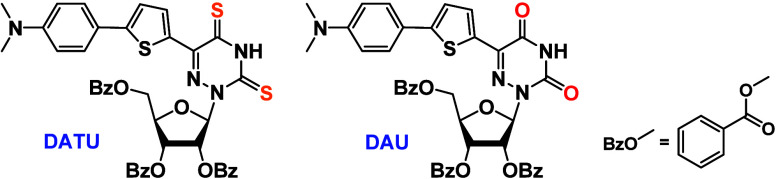
Structures of DATU (5-(5-(4-(Dimethylamino)­phenyl)­thiophen-2-yl)-2′,3′,5′-tri-*O*-benzoyl-6-aza-2,4-dithiouridine) and Its Oxygen Congener, DAU (5-(5-(4-(Dimethylamino)­phenyl)­thiophen-2-yl)-2′,3′,5′-tri-*O*-benzoyl-6-azauridine)

The results presented in this study demonstrate that DATU features a one-photon absorption spectrum that extends up to 750 nm in aqueous solution and efficiently absorbs two photons at 800 nm in 1,4-dioxane. In addition, we investigated its electronic structure and photophysical properties, as well as its triplet-state properties following both one-photon and two-photon absorption, comparing these results with those of the carbonyl counterpart. Our findings indicate that DATU exhibits a large one-photon absorption in the visible region, with an extinction coefficient of ε_510_ = (1.00 ± 0.05) × 10^4^ M^–1^ cm^–1^ in benzene. It also has a triplet-state lifetime of 1.7 ± 0.1 μs and a singlet oxygen quantum yield of 56%. Notably, DATU exhibits the highest two-photon absorption cross section (2PACS) of 160 ± 20 GM among nucleoside analogs reported to date when excited at 800 nm. This 2PACS value is nearly three times larger than that of DAU, its carbonyl counterpart, which has a 2PACS of 55 ± 5 GM at 800 nm in 1,4-dioxane and previously held the record with a value of 90 ± 5 GM at 840 nm.^34^

Experiments using the 1,3-diphenylisobenzofuran (DPBF) and the hydroxyphenyl fluorescein (HPF) probes in polar organic solvents (methanol and acetonitrile), as well as in aqueous solution at pH 7.4, demonstrate the formation of singlet oxygen and hydroxyl radical species following the selective photoactivation of DATU at 525 nm. These results evidence that DATU can act both as a type I and type II photosensitizer depending on the cellular environment. Strikingly, *in vitro* investigations using 4T1 murine mammary carcinoma cells demonstrate that DATU acts as an efficient photosensitizer under both one-photon (525 nm) and two-photon (800 nm) excitation. These findings position DATU as the most promising nucleoside photosensitizer for deep-tissue treatment to date, laying the groundwork for future in-depth investigations into its use as a PDT agent for other cancer cell lines and *in vivo* studies.

## Results and Discussion

### Steady-State Absorption and Emission Spectra

Figure 1 compares the absorption spectra of DATU and DAU in 1,4-dioxane and benzene. In 1,4-dioxane, DATU shows absorption maxima at 295 nm (ε_295_ = (2.35 ± 0.05) × 10^4^ M^–1^ cm^–1^), 381 nm (ε_381_ = (2.30 ± 0.05) × 10^4^ M^–1^ cm^–1^), and 487 nm (ε_487_ = (1.00 ± 0.05) × 10^4^ M^–1^ cm^–1^). In comparison, DAU exhibits absorption bands at 314 nm (ε_314_ = (1.49 ± 0.05) × 10^4^ M^–1^ cm^–1^) and 425 nm (ε_425_ = (2.35 ± 0.05) × 10^4^ M^–1^ cm^–1^). In benzene, DATU displays absorption maxima at 292 nm (ε_292_ = (2.24 ± 0.05) × 10^4^ M^–1^ cm^–1^), 388 nm (ε_388_ = (2.18 ± 0.05) × 10^4^ M^–1^ cm^–1^), and 510 nm (ε_510_ = (1.00 ± 0.05) × 10^4^ M^–1^ cm^–1^), extending up to approximately 700 nm. For DAU, the absorption maxima appear at 318 nm (ε_318_ = (1.55 ± 0.05) × 10^4^ M^–1^ cm^–1^) and 435 nm (ε_435_ = (2.27 ± 0.05) × 10^4^ M^–1^ cm^–1^) in benzene. The observed redshift in band maxima with increasing solvent polarity indicates solvatochromic effects, which are typically seen in ππ* states and charge-transfer absorption bands in organic chromophores. Additionally, DATU does not exhibit fluorescence, as noted in other thiobases.^35^ This lack of fluorescence emission suggests that one-photon absorption of DATU primarily leads to nonradiative relaxation pathways, such as internal conversion or intersystem crossing. In contrast, DAU shows significant fluorescence, with a broad band maximum at 565 nm and fluorescence quantum yields of 15 ± 1% in 1,4-dioxane and 16 ± 1% in benzene (Figure 1b). The excitation spectra of DAU closely resemble the experimental absorption spectra in both solvents, confirming their optical purity (Figure S1). Furthermore, the fluorescence band aligns well with observations for the ribose sugar deprotected DAU in 1,4-dioxane.^34^

**Figure 1:**
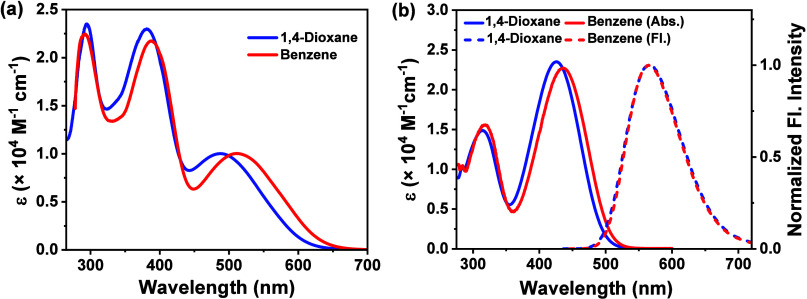
(a) UV–vis absorption spectra of DATU, and (b) UV–vis absorption and fluorescence spectra of DAU in 1,4-dioxane and benzene. The fluorescence spectra were collected exciting DAU at 425 and 435 nm, respectively.

### Ground- and Excited-State Calculations

Ground-state quantum-chemical calculations were performed for DATU and DAU at the B3LYP_G_-D3BJ/def2-TZVPD/CPCM level of theory in 1,4-dioxane and benzene, and the optimized ground-state geometries are presented in Figure S2. These calculations indicate that both DATU and DAU exist as two rotamers in the ground state, referred to as DATU_syn_ and DATU_anti_, as well as DAU_syn_ and DAU_anti_ (Scheme 2). For DATU, the *syn*-rotamer is predicted to be 2.68 kJ mol^–1^ more stable than *anti*-rotamer. This energy difference results in a Boltzmann population distribution of approximately 75% DATU_syn_ and 25% DATU_anti_, similar to that found in a previous study on a related 6-azauridine derivative.^36^ For DAU, the *syn*-rotamer is estimated to be more stable than the *anti*-rotamer by 3.91 kJ mol^–1^, leading to a rotamer distribution of approximately 83% *syn* and 17% *anti.*

**Scheme 2:**
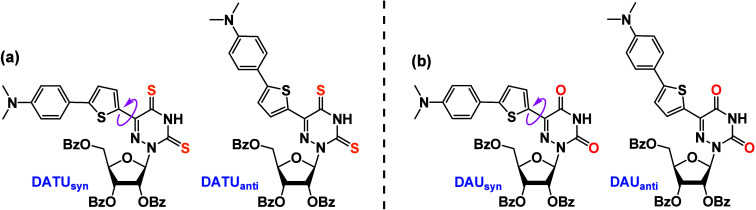
Structures of *syn*- and *anti*-Rotamers of (a) DATU and (b) DAU^a^

a The "*syn*"/"*anti*" notation is used here concerning the N6 and thiophene’s S atoms of the DATU molecule.

Excited-state calculations were performed in the Franck–Condon region for DATU and DAU at the TD-X/def2-TZVPD/CPCM level of theory in 1,4-dioxane and benzene. Here, X denotes one of the following functionals: M062X, CAM-B3LYP, X3LYP, PBE0, or B3LYP. These calculations enable the characterization of their electronic transitions, the evaluation of the energies and characters of excited states, and the determination of spin–orbit coupling constants (SOCs) for the ISC pathways from singlet to triplet states upon excitation. Among the tested functionals, M062X and CAM-B3LYP best reproduced the experimental absorption spectra (see SI and Figures S3–S6) while effectively accounting for the significant charge-transfer (CT) character of the singlet excited state in these molecules (Figure 2). For simplicity, the results for DATU and DAU in 1,4-dioxane using the M062X functional are discussed here, as nearly identical results were obtained in both solvents (see Tables S1–S9 and Tables S10–S17). Additionally, both the DATU_syn_ and DATU_anti_ rotamers display very similar vertical electronic transitions and state characteristics (see Tables S1 and S2). Consequently, the Kohn–Sham orbitals for only the major isomer, DATU_syn_, are depicted in Figure 2. The simulated absorption spectra align well with the experimental spectra (Figure 2), falling within the expected accuracy range of these calculations of ± 0.2 to 0.3 eV.^37^ The lowest-energy band comprises S_1_(*n*π*) and S_2_(ππ*) absorptions, which are nearly isoenergetic (2.70 and 2.77 eV for DATU_syn_, and 2.68 and 2.72 eV for DATU_anti_, respectively). The second-lowest absorption band predominantly corresponds to the S_3_(ππ*) state transition, occurring at approximately 3.5 eV for both rotamers, while the highest energy band consists of multiple higher-order singlet (S_*n*_) transitions.

**Figure 2:**
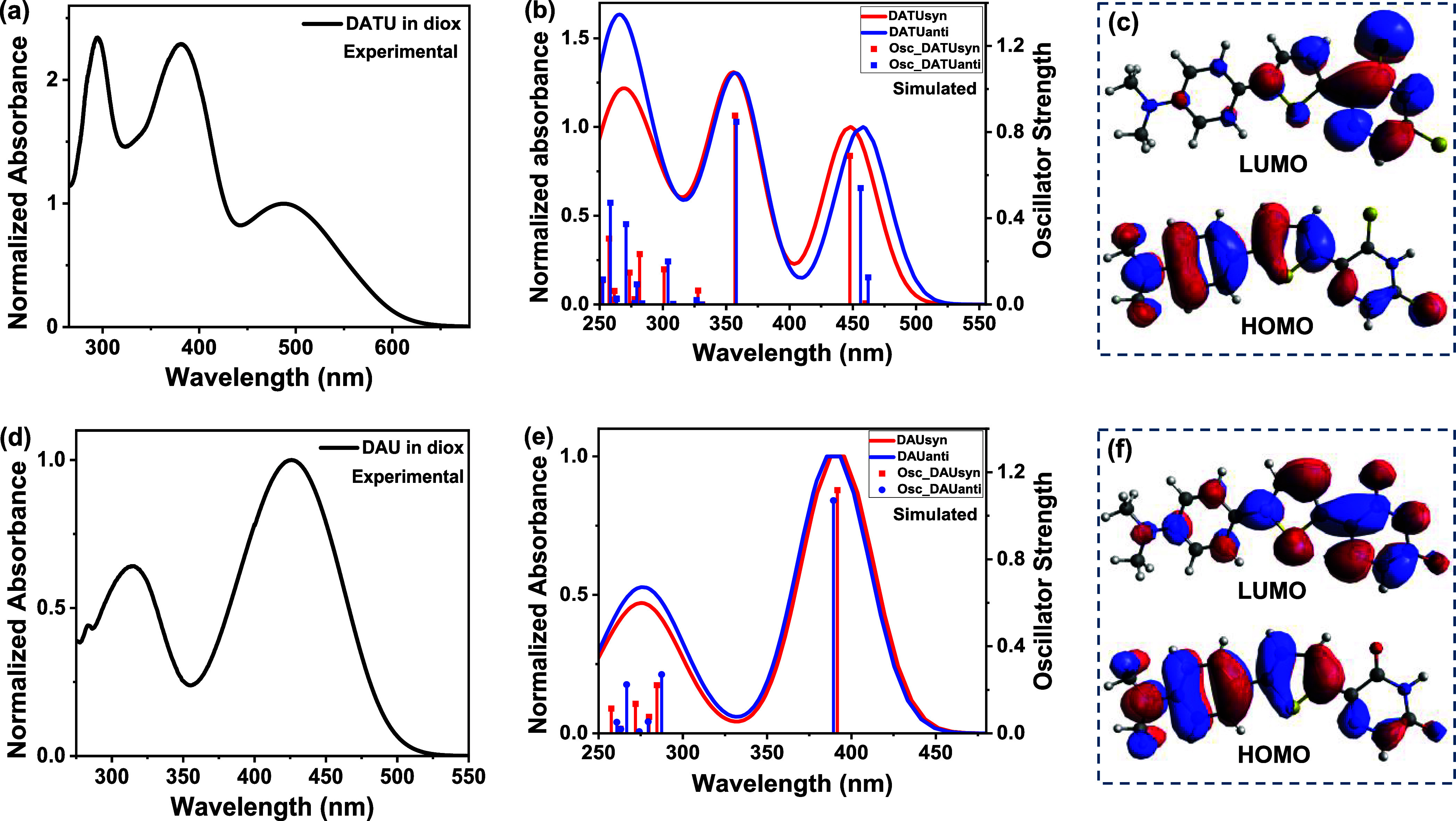
Experimental absorption spectra for (a) DATU and (d) DAU in 1,4-dioxane, alongside the simulated absorption spectra for (b) DATU and (e) DAU in 1,4-dioxane. The corresponding Kohn–Sham orbitals for HOMO and LUMO are shown for (c) DATU and (f) DAU.

The S_1_(*n*π*) state features nonbonding orbitals localized on the sulfur atoms of the thiocarbonyl groups within the 6-aza-2,4-dithiouridine moiety. This is consistent with typical thionated systems, with the HOMO-2 and HOMO-3 serving as the dominant contributors of the *n*-orbitals. The S_2_(ππ*) state exhibits CT character, where the HOMO primarily localized on the 5-(4-(dimethylamino)­phenyl)­thiophene, while the LUMO is localized on the 6-aza-2,4-dithiouridine moieties. The S_3_(ππ*) state corresponds to a transition from HOMO to LUMO+1, with LUMO+1 largely localized on the thiophene and 6-aza-2,4-dithiouridine moieties. This indicates a combination of locally excited (LE) and CT states.

The simulated absorption spectra for DAU in both solvents closely match the experimental spectra, with the lowest-energy absorption band corresponding to the S_1_(ππ*) transition (Figure 2). This S_1_(ππ*) state corresponds to the transition from HOMO to LUMO and displays a mixed LE and CT character. In contrast, the S_2_(ππ*) state of DATU predominantly exhibits CT character. This difference is linked to the electron density distributions of the HOMO and LUMO in both molecules: The HOMO of DAU has an electron density distribution like that of DATU, while their LUMOs differ significantly (refer to Figure 2). In DAU, the LUMO is more delocalized across the thiophene and 6-azauridine moieties, whereas in DATU, it is more localized on the 6-azadithiouridine moiety. Additionally, the calculated dipole moments for the S_0_ geometries are approximately 8.1 D for the *syn*-rotamer and 6.7 D for the *anti*-rotamer of DAU, compared to 10.1 D for the *syn*-rotamer and 8.9 D for the *anti*-rotamers of DATU. These results indicate that the thionation of DAU increases both the ground-state dipole moment of the molecule and enhances the electron-accepting properties of the 6-azadithiouridine moiety. This leads to a greater intramolecular CT character during excitation at the lowest-energy absorption band.

The SOCs for DATU and DAU were also calculated using the optimized S_0_ geometries (Tables S6 and S14). The SOCs are significantly larger for DATU than for DAU, indicating a greater likelihood of intersystem crossing (ISC) in DATU. Since both rotamers of each molecule show very similar SOCs (refer to Tables S2, S4, S10, and S12), this discussion will focus exclusively on the *syn*-rotamers, which are the major isomers. For *syn*-DATU, the S_1_(*n*π*) → T_4_(ππ*), S_1_(*n*π*) → T_3_(ππ*), and S_1_(*n*π*) → T_1_(ππ*) transitions have large SOC values of 66.2, 112.1, and 41.2 cm^–1^, respectively. This indicates that the S_1_(*n*π*) serves as the primary ISC doorway state, as it is nearly isoenergetic with the S_2_(ππ*) state within the limits of computational accuracy. Conversely, for the *syn*-DAU, the S_1_(ππ*) → T_2_(ππ*) and S_1_(ππ*) → T_1_(ππ*) pathways exhibit SOCs of only 0.3 and 0.1 cm^–1^, respectively, suggesting significantly lower probability for ISC to the triplet manifold. This finding agrees with the El-Sayed propensity rules.^38,39^ The predicted decrease in ISC pathways for DAU compared to DATU corresponds with DAU’s relatively high fluorescence yield. The predicted electronic relaxation pathways derived from these quantum-chemical calculations are illustrated in Figure 3 for both DATU and DAU.

**Figure 3:**
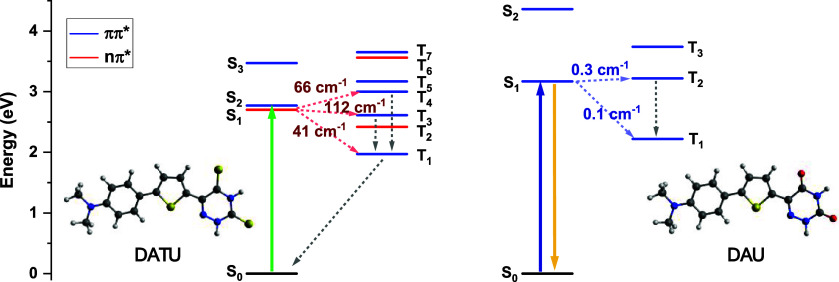
Jabłoński diagram for the major (*syn*) isomers of DATU and DAU, illustrating the dominant relaxation pathways following excitations to the respective lowest-energy absorption bands.

Geometry optimizations were performed for the S_1_, S_2_, and T_1_ states of DATU and for the S_1_ and T_1_ states of DAU to probe possible excited-state relaxation pathways. Due to the high computational cost of these large structures, calculations were restricted to the *syn*-rotamers (major) in 1,4-dioxane. Convergence was achieved only with the B3LYP functional among other functionals (CAM-B3LYP and X3LYP); thus, all optimized structures were obtained at the TD-B3LYP/def2-TZVPD/CPCM level of theory, except for the T_1_ state of DAU, which did not converge. The optimized geometries show that, for DATU, the S_2_ and T_1_ states are planar, whereas the S_1_ state exhibits a strongly twisted 6-azadithiouracil ring with a torsion angle ≈ 90° (Figure S7). In contrast, the S_1_ state of DAU remains nearly planar. Simulated excited-state absorption spectra calculated at the optimized DATU geometries using M062X indicate similar bands for S_1_ and T_1_, while the S_2_ band is red-shifted by approximately 70 nm (Figure S8). These simulated spectra are not expected to directly model experimental transient absorption features, which are strongly influenced by overlapping ground-state bleach and charge-transfer contributions (for DATU) or by stimulated emission (for DAU).

### Femtosecond Transient Absorption Spectroscopy

Femtosecond transient absorption spectroscopy (fs-TAS) was used to experimentally elucidate the electronic relaxation pathways of DATU and DAU, comparing the results to the quantum-chemical predictions presented in Figure 3. Excitation at 520 nm primarily populated the S_2_(ππ*) state in DATU, while excitation at 400 nm predominantly accessed the S_1_(ππ*) state in DAU, in both 1,4-dioxane and benzene. The fs-TAS spectral features and kinetics for each compound are nearly identical in both solvents. Consequently, the spectral evolution, evolution-associated difference spectra (EADS), and representative kinetic traces for DATU and DAU in 1,4-dioxane are presented in Figures 4 and 5, respectively. The corresponding fs-TAS data in benzene are shown in Figures S9 and S10.

**Figure 4:**
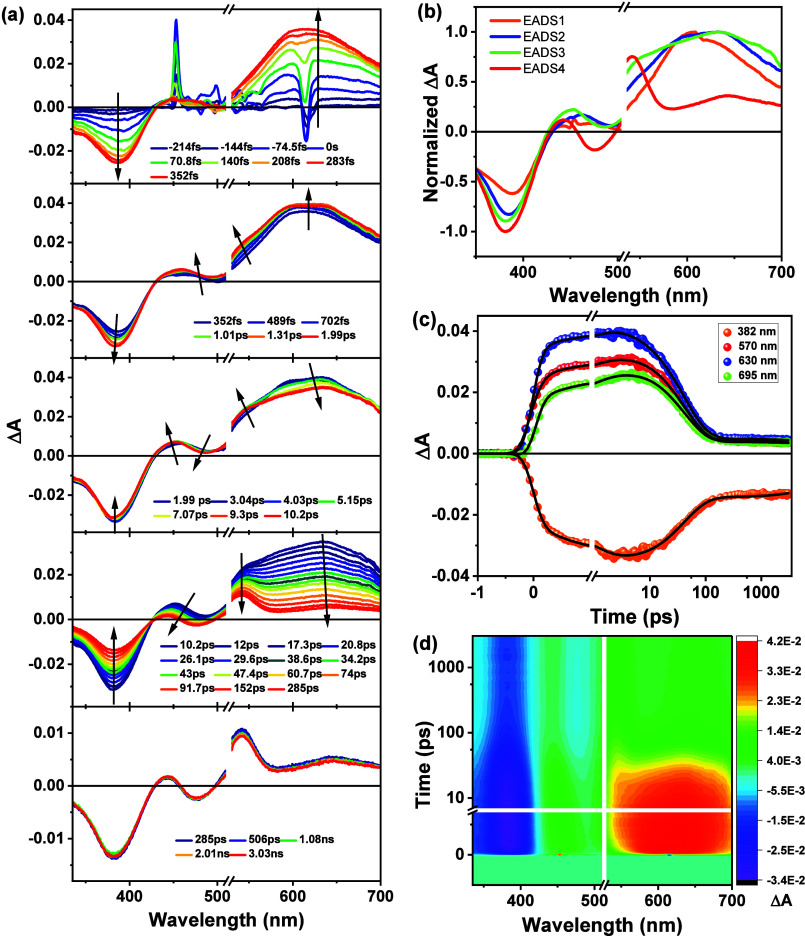
(a) Spectral evolution of fs-TAS of DATU in 1,4-dioxane at 520 nm excitation, (b) extracted EADS, (c) kinetic decay traces with fits at different wavelengths, and (d) contour plots of fs-TAS. The breaks on the *x*-axis of (a, b, and d) cover the scattering from the pump pulse, and those on the *x*-axis in (c) and the *y*-axis in (d) represent the change in the scale from linear to logarithmic. The sharp signals in the top panel of (a) correspond to the stimulated Raman scattering and coherent solvent signals, which were used to define time zero at its maximum amplitude.

**Figure 5:**
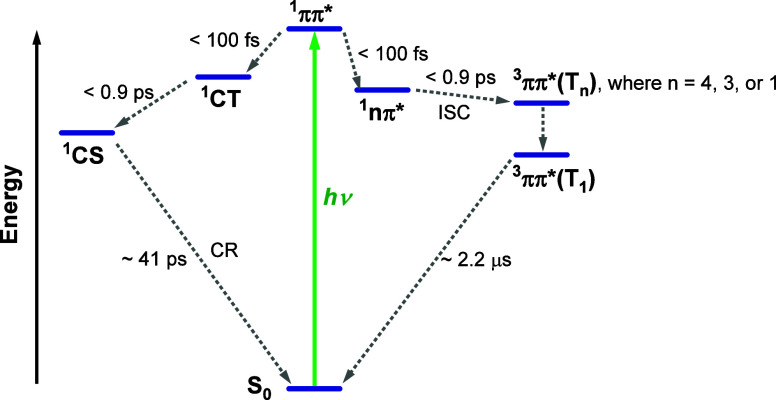
A qualitative relaxation mechanism for DATU upon photoexcitation.

For DATU, the fs-TAS spectra reveal complex dynamics in the excited states and display a distinct combination of ground-state bleach (GSB) and excited-state absorption (ESA). Specifically, there is a negative GSB band at 383 nm and a broad positive ESA centered at 615 nm. The ESA observed within the pump–probe cross-correlation of approximately 300 fs after photoexcitation (Figure 4a, top panel) is attributed to a combined population of the vibrationally excited ^1^ππ*­(CT state) and ^1^ *n*π*­(S_1_) states in both *syn*- and *anti*-rotamers, which coexist in solution. The initially populated ^1^ππ*­(S_2_) state is proposed to branch into two pathways, forming a ^1^ππ*­(CT state) as indicated from the featureless broad ESA band, and the vibrationally excited ^1^nπ*­(S_1_) state following ultrafast internal conversion (ca. <100 fs). Subsequently, during approximately 1.5 ps, the overall spectra, including both GSB and ESA bands, continue to increase slightly in amplitude, indicative of the possible growth of a new state, and a shoulder appears at around 540 nm, attributed to the observation of triplet states (Figure 4a, second panel). This observation is attributed to the simultaneous population of two states: (1) a charge-separated state (CS) arising from the previously formed ^1^ππ*­(CT) state and (2) hot triplet states generated from the previously populated ^1^ *n*π*­(S_1_) state. Spectral shifts are noted within approximately 10 ps (Figure 4a, third panel), which are ascribed to a combination of conformational and solvent relaxations of the CS state and hot triplet states relaxing to populate the relaxed ^3^ππ*­(T_1_) state in both *syn*- and *anti*-rotamers. Over the next ∼200 ps, the GSB and ESA bands decay, with the 540 nm shoulder becoming prominently visible, thereby highlighting the long-lived ESA (Figure 4a, fourth panel). This process corresponds mainly to the charge-recombination (CR) of the CS state, with the population returning to the ground state. After about 285 ps, the spectra remain largely unchanged for up to 3 ns (Figure 4a, fifth panel) due to the long-lived nature of the ^3^ππ*­(T_1_) state. Global analysis of the fs-TAS data reveals four lifetimes: 0.90 ± 0.08 ps (0.44 ± 0.05 ps in benzene), 5.0 ± 0.7 ps (3.9 ± 0.4 ps in benzene), 41 ± 2 ps (56 ± 4 ps in benzene), and >2 ns (>2 ns in benzene). The first lifetime is assigned to a combination of ultrafast intramolecular CS state population and ^1^ππ* → ^1^ *n*π* → ^3^ππ* ISC. The second lifetime is assigned to conformational relaxation and solvent reorganization, the third lifetime to CR to the ground state from the CS state, and the fourth lifetime to the long-lived triplet state (see Figure 5). The associated EADS are shown in Figure 4b, while representative kinetic fits and contour plots are depicted in Figure 4c and Figure 4d, respectively. The slower conformational relaxation of DATU in 1,4-dioxane compared to benzene likely stems from the higher viscosity of 1,4-dioxane (η ≈ 1.2 cP) versus that of benzene (0.6 cP). The ISC lifetimes (0.90 ± 0.08 ps in 1,4-dioxane and 0.44 ± 0.05 ps in benzene) are in good agreement with those reported for other thiobase analogs,^3,7^ which typically exhibit ISC lifetimes in the subpicosecond range. No emission is observed from the S_2_ state of DATU, likely due to ultrafast excited-state branching and pronounced charge-transfer character, which promote efficient nonradiative relaxation to the ground state, further reinforced by energy gap law–driven enhancement of nonradiative decay pathways.^40–44^

Figure 6 illustrates the fs-TAS data for DAU, revealing more complex dynamics compared to DATU. These dynamics are characterized by competitive interplay among GSB, ESA, and stimulated emission (SE). The transient spectra exhibit a prominent GSB band at 420 nm, a broad positive ESA at 645 nm, and a faint SE feature at 532 nm within the initial ∼300 fs time window (top panel, Figure 6a). Following these processes, three successive relaxation processes are evident, showing minor spectral shifts within the time windows of ∼2.5 and ∼9 ps (second and third panels, Figure 6a) and ∼40 ps (fourth panel, Figure 6a). Subsequently, the transient absorption species gradually decrease over the 2.4 ns observation window (bottom panel, Figure 6a). The global analysis of the fs-TAS data identifies five lifetimes: 0.79 ± 0.05 ps (τ_1_, 0.70 ± 0.05 ps in benzene), 3.2 ± 0.4 ps (τ_2_, 2.8 ± 0.4 ps in benzene), 16.1 ± 0.8 ps (τ_3_, 10.0 ± 0.9 ps in benzene), 750 ± 100 ps (τ_4_, 650 ± 100 ps in benzene), and >2.4 ns (τ_5_, > 2.4 ns in benzene). The associated EADS and kinetic fits are shown in Figure 6b and Figure 6c, respectively. These lifetimes and spectral changes are attributed to conformational/vibrational relaxation, solvent relaxation, and partial decay of the S_1_ state from both rotamers of DAU. This interpretation is supported by the observation that the GSB signal at 422 nm remains virtually constant within the first approximately 50 ps time window (Figure 6c), as well as by quantum-chemical calculations reported in the previous section (see also Figure 3). Like DATU, conformational/solvent relaxation occurs slightly more slowly in 1,4-dioxane than in benzene, which can be attributed to the higher viscosity of 1,4-dioxane.

**Figure 6:**
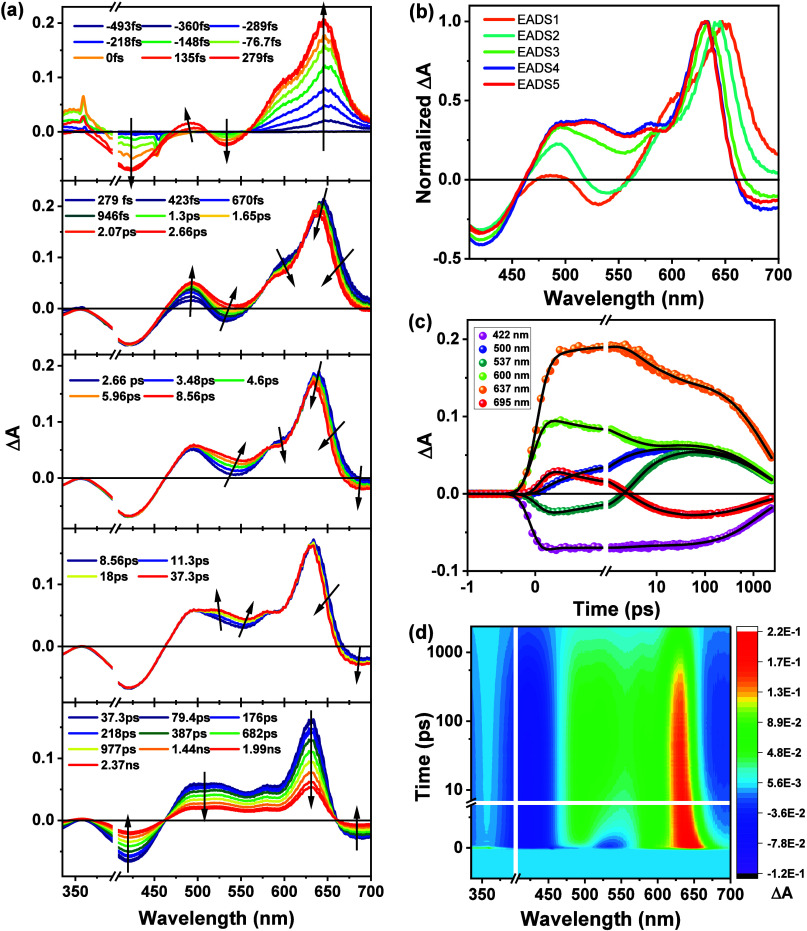
(a) Spectral evolution of fs-TAS of DAU in 1,4-dioxane at 400 nm excitation, (b) extracted EADS, (c) kinetic decay traces with fits at different wavelengths, and (d) contour plots of fs-TAS. The breaks on the *x*-axis of (a and d) cover the scattering from the pump pulse, and those on the *x*-axis of (c) and the *y*-axis in (d) represent the change in the scale from linear to logarithmic. The sharp signals in the top panel of (a) correspond to the stimulated Raman scattering and coherent solvent signals, which were used to define time-zero at its maximum amplitude.

### Picoseconds to Microseconds Transient Absorption Spectroscopy and Determination of Singlet Oxygen Quantum Yields

The fs-TAS data shown in Figures 4 and 6 and Figures S9 and S10 for DATU and DAU illustrate the presence of long-lived transient species with decay times longer than 2.4 ns. To assess the lifetimes of these long-lived transient species, we conducted transient absorption spectroscopy experiments in the picosecond-to-microsecond range. The microsecond transient absorption data for DATU and DAU are presented in Figures 7 and 8 in 1,4-dioxane, while the transient data in benzene are shown in Figures S11 and S12. In both solvents, the overall transient absorption spectra indicate a combination of competing contributions from GSB and EAS. The spectral features and the decay kinetics are similar in both solvents. Consequently, this discussion will focus solely on the data obtained in 1,4-dioxane.

**Figure 7:**
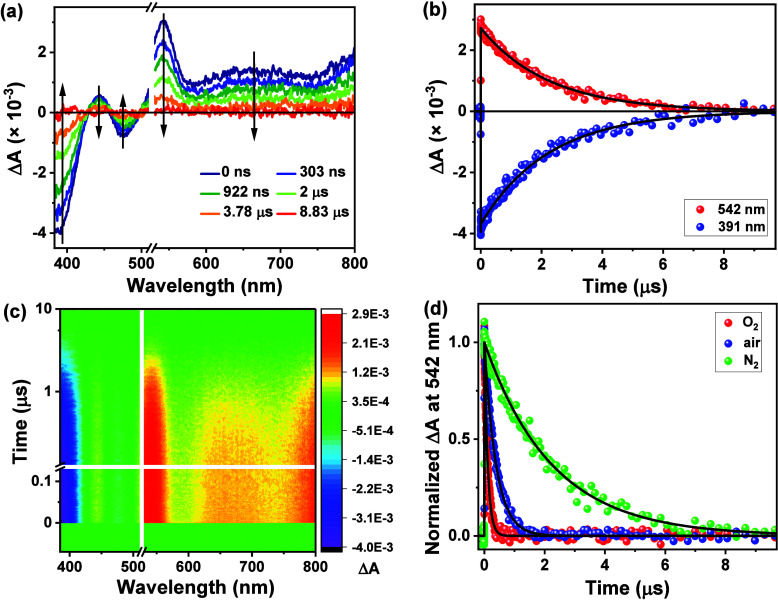
(a) ps-to-μs-TAS of DATU, (b) respective kinetic traces, and (c) contour plots for TAS data in N_2_-saturated 1,4-dioxane following excitation at 520 nm. Breaks on the *x*-axis of (a and c) cover the scattering from the pump pulse, while that on the *y*-axis of (c) represents the change in the scale from linear to logarithmic. (d) Representative kinetic traces of TAS recorded under O_2_, air, and N_2_-saturated conditions.

**Figure 8:**
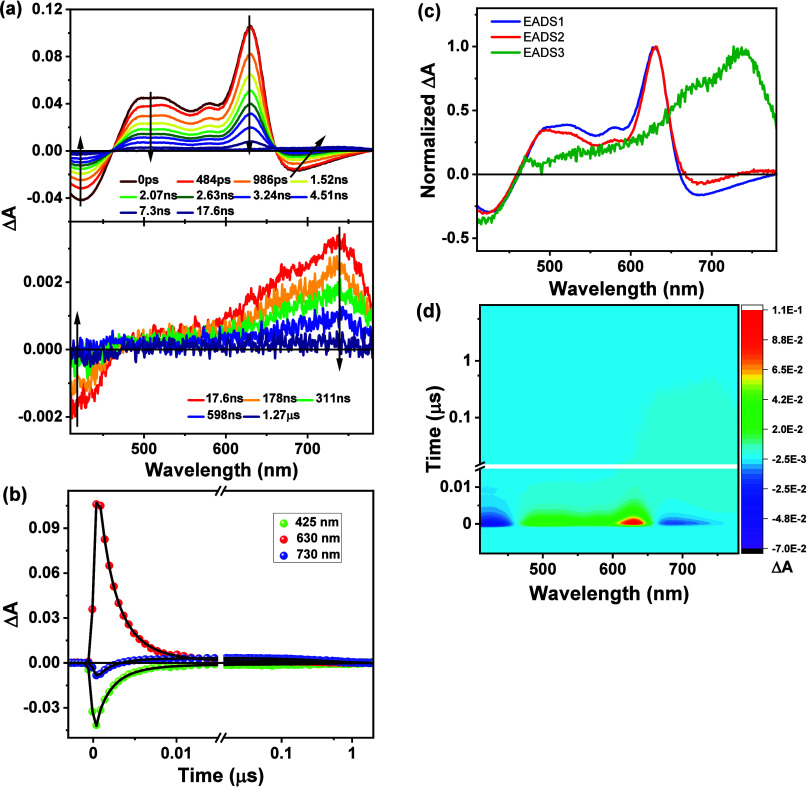
(a) ps-to-μs-TAS of DAU, (b) respective kinetic traces, (c) EADS in air-equilibrated 1,4-dioxane following excitation at 400 nm, and (d) contour plots of TAS. Breaks on the *x*-axis of (b) and the *y*-axis in (d) represent the change in the scale from linear to logarithmic.

Figure 7a illustrates the transient absorption spectra of DATU when excited at 520 nm under N_2_-saturated conditions in 1,4-dioxane. Figure 7b and Figure 7c present the kinetic decay traces and the contour plot, respectively. Two negative-amplitude bands are detected: a larger band around 390 nm and a weaker one near 480 nm, both attributed to GSB. Additionally, a transient absorption species with maxima at approximately 445, 542, 645 nm, and above 800 nm is observed. This ESA signal is attributed to triplet-state absorption, as confirmed by quenching kinetics under air and O_2_-saturated conditions (Figure 7d and Figure S13). The transient data decay monotonically to the baseline within approximately 8 μs in both 1,4-dioxane and benzene under N_2_-saturated conditions. Global analyses reveal the lifetimes of the excited triplet states. A single-component model satisfactorily fits the data in both solvents, yielding triplet-state lifetimes of 2.2 ± 0.1 μs in 1,4-dioxane and 1.73 ± 0.10 μs in benzene under N_2_-saturated conditions. Under air- and O_2_-saturated conditions, these lifetimes decreased to 390 ± 15 and 110 ± 5 ns in 1,4-dioxane and to 265 ± 10 and 75 ± 4 ns in benzene, respectively, leading to bimolecular quenching rate constants of 1.4 × 10^9^ M^–1^ s^–1^ for both solvents, considering the reported oxygen concentrations in both solvents.^45^ Representative kinetic traces under these conditions are depicted in Figure 7d and Figure S11d. The generation of singlet oxygen quantum yield (Φ_Δ_) was quantified using the ^1^O_2_ sensitive probe, DPBF,^46–49^ and tetraphenylporphyrin (TPP) as the reference standard in benzene (Φ_Δ_ = 0.62, 355 nm excitation).^50,51^ Figure S14 shows that DATU has a Φ_Δ_ of 0.56 ± 0.04 in benzene. These results demonstrate that DATU is a promising PDT agent, exhibiting microsecond triplet-state lifetimes in both 1,4-dioxane and benzene, along with a 56% singlet oxygen yield in benzene. Based on the combined results from experimental transient absorption spectroscopy and quantum-chemical calculations, a qualitatively proposed relaxation mechanism for DATU is presented in Figure 5.

The picoseconds-to-microsecond TAS measurements were conducted for DAU to capture the complete decay dynamics of the long-lived transient species (Figure 8) and to complement the femtosecond TAS results reported in the previous section. The spectral features and dynamics are consistent across both solvents, aligning with observations in the femtosecond to two-nanosecond time window (Figure 6). The spectral evolution, EADS, and kinetic traces of DAU are shown in Figure 8 in air-equilibrated 1,4-dioxane, while those in benzene are presented in Figure S12. The transient spectra show a rapid decay in amplitude, accompanied by the emergence of a positive band near 740 nm (as indicated by the arrows), with approximately 97% of the population decaying within about 18 ns, relative to the GSB band at ∼422 nm (top panel, Figure 8a). This process corresponds to the branching and decay of the S_1_ population, primarily returning to the ground state via fluorescence emission, with a minor fraction (around 3%) undergoing ISC to the triplet manifold. The dynamics involve the relaxation of both rotamers. The transient absorption species, with a maximum at ca. 740 nm, decays monotonically to the baseline within ∼1.2 μs (bottom panel, Figure 8a), indicating the deactivation of the T_1_ state. This assignment is further supported by measurements conducted under N_2_-saturated conditions (Figure S15). A global analysis of the transient data reveals three lifetimes: 1.0 ± 0.3 ns (1.0 ± 0.3 ns in benzene), 2.7 ± 0.3 ns (3.0 ± 0.3 ns in benzene), and 465 ± 25 ns (298 ± 20 ns in benzene) in 1,4-dioxane. These correspond to the S_1_-state fluorescence decay of the two rotamers (τ_1_ and τ_2_) and the triplet lifetime (τ_3_), respectively. Under N_2_-saturated conditions, τ_3_ increases to 4.9 ± 0.2 μs (1.04 ± 0.10 μs in benzene) in 1,4-dioxane. The two S_1_-state lifetimes are consistent with those obtained from fs-TAS data (Figure 6) and align well with previously reported fluorescence lifetimes for ribose-deprotected DAU rotamers in 1,4-dioxane.^34^ The transient absorption data demonstrate that excited-state dynamics of DAU consist of both nanosecond fluorescence and microsecond ISC decay pathways, with the fluorescence decay pathways accounting for approximately 15% of the fluorescence quantum yield.

### Two-Photon Absorption at 800 nm and Determination of the Two-Photon Absorption Cross Sections

The transient absorption data collected over femtosecond to microsecond time scales, as discussed in the previous section, show that both DATU and DAU populate long-lived excited states that can decay over several microseconds. Notably, DATU primarily undergoes nonradiative decay from its triplet excited state, whereas DAU mainly decays via radiative emission, with only about 3% of its decay occurring nonradiatively from the triplet state. These findings suggest that DATU should serve as an effective photosensitizer for PDT applications, while DAU could function as an effective fluorophore for bioimaging purposes.^34^ Two-photon excitation at this wavelength represents a significant breakthrough for improving light penetration in deep-tissue applications. Consequently, we conducted two-photon absorption experiments for both DATU and DAU using femtosecond transient absorption spectroscopy at 800 nm.^52^ To determine the two-photon absorption cross sections for DATU and DAU, we used Rhodamine 6G as a standard under identical experimental laser conditions (73 GM at 800 nm,^53^ Figure 9). It is important to note that while two-photon excited fluorescence is commonly employed to assess the two-photon absorption characteristics of molecules,^53,54^ this method requires the compound to be intrinsically fluorescent, which is not the case for DATU. Our group^55^ and others^52^ have shown that fs-transient absorption spectroscopy can successfully be used as an alternative spectroscopic technique to measure the two-photon absorption properties of molecules in solution, including accurate two-photon absorption cross sections.

**Figure 9:**
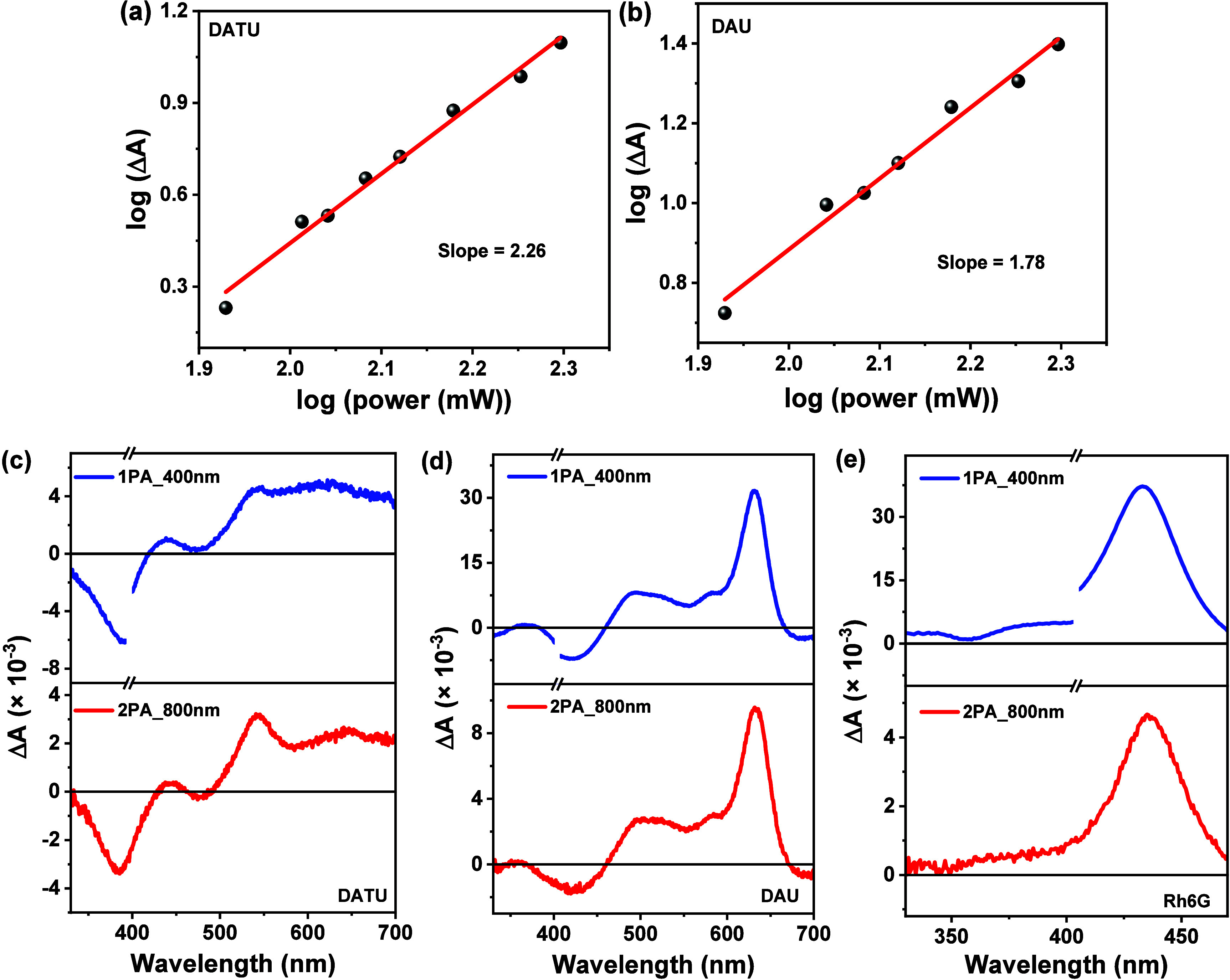
Log–log plots of Δ*A* versus the power of 800 nm fs-pulsed laser for (a) DATU and (b) DAU in 1,4-dioxane. The transient absorption spectra of (c) DATU and (d) DAU in 1,4-dioxane, and (e) Rhodamine 6G in methanol at 20 ps delay, recorded upon 400 nm one-photon (top panel) and 800 nm two-photon (bottom panel) excitations. The breaks in the *x*-axis of the top panels in (c, d, and e) are covering scattering from the 400 nm pump pulse.

The dependence of the transient absorption signal on the laser beam power at 800 nm was measured under identical experimental conditions for both DATU and DAU. The change in absorbance (Δ*A*) at the wavelength of maximum excited-state absorption was plotted against the laser power at 800 nm (see Figure 9a,b). A log–log plot of Δ*A* versus excitation power revealed slopes of 2.26 ± 0.05 for DATU and 1.78 ± 0.05 for DAU. The deviations of the slopes from the expected quadratic behavior (i.e., a value of 2) are primarily due to experimental uncertainties in the collected data, as demonstrated in the Supporting Information (SI, Figure S16). These results indicate that a two-photon absorption process governs the excited-state population under the experimental conditions used. We applied a time delay of 20 ps to measure Δ*A* for DATU, DAU, and Rhodamine 6G, which corresponds to the excited singlet-state ESA for DAU and Rhodamine 6G, and a combined excited singlet- and triplet-state ESA for DATU. The similarities between the spectra obtained under one-photon (400 nm) and two-photon (800 nm) excitation support our methodological approach (see Figure 9c–e).

The two-photon absorption cross sections measured at 800 nm were 160 ± 20 GM for DATU and 55 ± 5 GM for DAU (Goeppert–Mayer units; 1 GM = 10^–50^ cm^4^ s), both evaluated under identical conditions in 1,4-dioxane (see Figure 9c,d). These values are relative to the standard Rhodamine 6G (Figure 9e). The 55 GM value for DAU is 1.6 times smaller than the previously reported value of 90 ± 5 GM at 840 nm for the ribose-deprotected DAU in 1,4-dioxane.^34^ This discrepancy in the two-photon absorption cross section values for DAU is not unexpected and can primarily be attributed to the different excitation wavelengths used (800 versus 840 nm) and the varying experimental methodologies.

Notably, the two-photon absorption cross section of DATU is 3-fold larger than the one measured for DAU under the same experimental conditions. In fact, DATU exhibits the largest two-photon absorption cross section among all nucleoside analogs investigated to date.^34,56–59^ It is generally understood that the presence of stronger donor−π–acceptor (D−π–A) motifs, or larger transition dipole moments, correlates with an increased in the two-photon absorption cross sections.^60,61^ From this perspective, one might expect the carbonyl-containing DAU to exhibit a larger two-photon absorption cross section than DATU, given the greater electronegativity of the carbonyl oxygen atom (3.44) compared to the thiocarbonyl sulfur atom (2.58). This suggests that DAU should possess a stronger electron-accepting character. However, the excited-state calculations reported in Figure 2 show that the LUMO is predominantly localized on the 6-azadithiouridine moiety in DATU, while it is delocalized across both the 6-azauridine and thiophene units in DAU. This indicates that thionation enhances the electron-accepting ability of the 6-azauridine core. Notably, the HOMO remains largely unchanged for both compounds (Figure 2). This trend is consistent with earlier studies on thionated analogs, which employed natural bond orbital (NBO) analysis to demonstrate that thionation stabilizes the LUMO and enhances the electron-accepting properties compared to its oxygen analog.^62^ This stabilization of the LUMO is believed to arise from the weaker orbital overlap between the 3p orbital of sulfur and the 2p orbital of carbon in the C=S bond, compared to the stronger 2p­(C)–2p­(O) interaction in the C=O bond.^62^ Consequently, the π*­(C=S) antibonding orbital—a significant contributor to the LUMO—undergoes less antibonding destabilization, thus lowering its energy relative to the carbonyl analog.^62^ Consistent with this argument, our calculations for DAU and DATU reveal comparable trends in the relative HOMO and LUMO energies in both solvents (Table S18). In 1,4-dioxane, the HOMO energies of DATU_syn_ and DAU_syn_ are very similar (−6.341 and – 6.293 eV, respectively). In contrast, the LUMO of DATU_syn_ is significantly more stabilized (−2.093 eV) than that of DAU_syn_ (−1.531 eV). The same pattern is observed for the corresponding *anti*-rotamers in 1,4-dioxane and for both *syn*- and *anti*-rotamers in benzene (Table S18).

### Singlet Oxygen Generation in DATU upon Two-Photon Absorption at 800 nm

We have shown that DATU can be effectively excited by two-photon absorption in the near-infrared (NIR) range. Now, we investigate whether singlet oxygen is generated, which is essential for two-photon PDT applications. To conduct these measurements, we used DPBF as a singlet oxygen sensor in benzene. DPBF is a well-established and highly sensitive probe that acts as a chemical scavenger for detecting and quantitatively determining singlet oxygen.^46–49^ Figure 10 illustrates that the absorption spectrum of DPBF, measured between approximately 300 and 460 nm, decreases over time when the DPBF and DATU mixture is excited at 800 nm. In contrast, no change is observed when DPBF alone is irradiated under the same conditions for the same duration. These results clearly demonstrate that ^1^O_2_ is generated upon two-photon excitation of DATU at 800 nm in benzene, leading to the degradation of DPBF. Therefore, the findings of this study confirm that DATU can generate ^1^O_2_ under both one-photon and two-photon absorption conditions. Alongside recent progress in other organic thionated chromophores as photosensitizers for PDT,^63–66^ the development of DATU as a thiobase photosensitizer analog marks a key advance in this rapidly evolving field.

**Figure 10:**
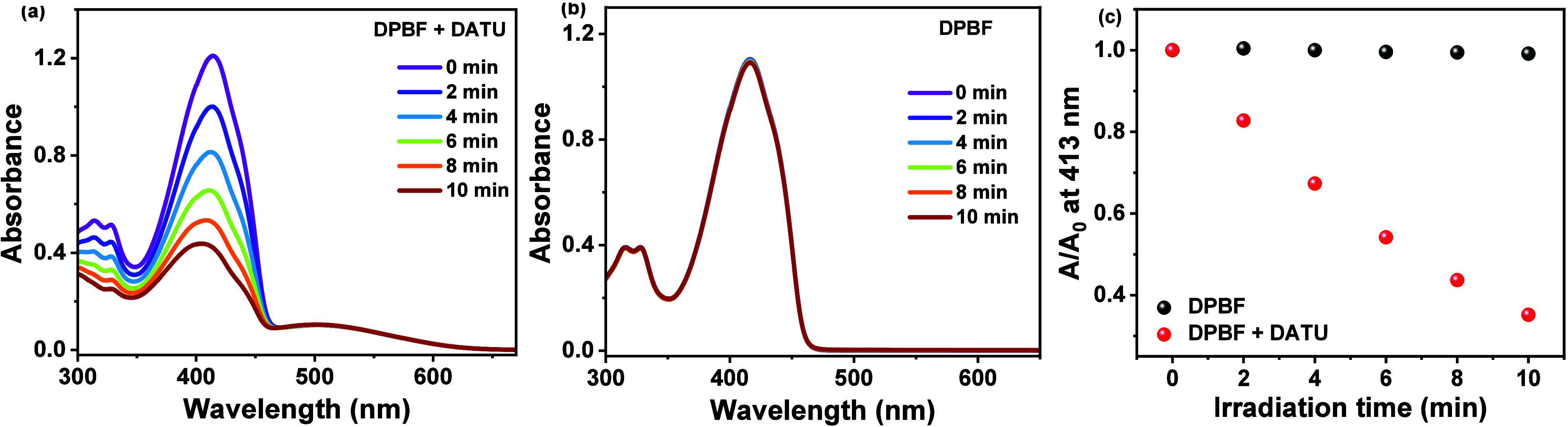
Degradation of DPBF under 800 nm fs-pulsed laser excitation for different irradiation times in (a) the presence and (b) the absence of DATU in benzene. (c) Corresponding plot of degradation (*A*/*A*_0_ vs laser irradiation time) of DPBF in both conditions under 800 nm fs-pulsed laser excitation.

### DATU Generates Reactive Oxygen Species under Physiologically Relevant Conditions and Cellular Environments

While we have demonstrated above that DATU exhibits the hallmarks of an efficient photosensitizer in the optical therapeutic window, evaluating its photosensitizing efficacy under physiologically relevant conditions and cellular environments is necessary because the photophysical properties of DATU are expected to be sensitive to the polarity of the solvent. To demonstrate that singlet oxygen continues to play a role in the PDT efficacy of DATU under physiological conditions that better mimic cellular environments, we conducted the DPBF assay in polar organic solvents, specifically methanol (MeOH) and acetonitrile (MeCN), using 525 nm irradiation.

In these solvents, activating DATU in air-saturated solutions of DATU/DPBF at 525 nm results in the degradation of DPBF (see Figure 11), indicating the generation of singlet oxygen. While these experiments cannot be performed in aqueous solutions using DPBF as a singlet oxygen probe because DPBF is insoluble in aqueous buffers, MeOH and MeCN provide representative polar environments that reflect the diverse microenvironments and local polarities found in cellular organelles.

**Figure 11:**
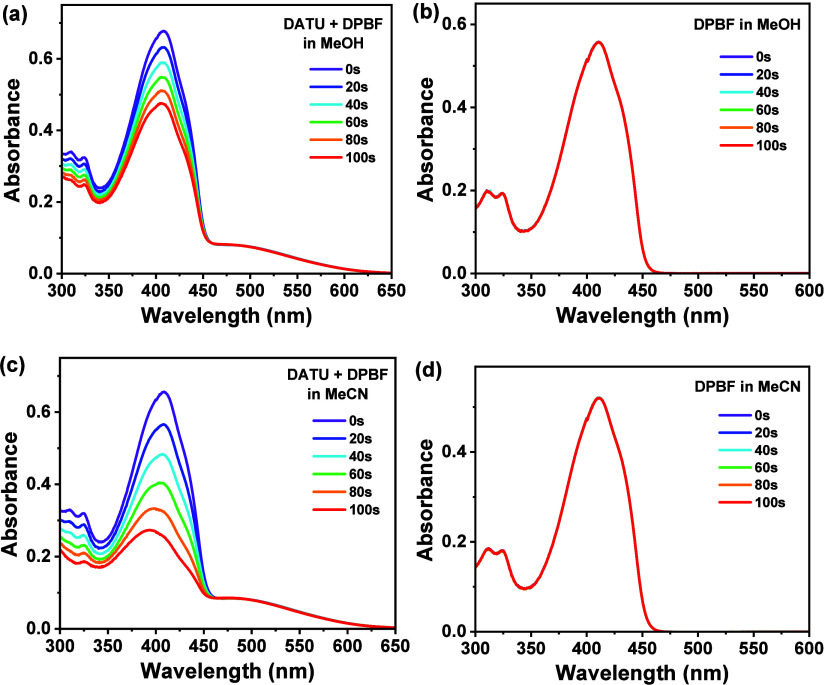
DPBF assay demonstrates the generation of singlet oxygen upon irradiation of DTAU/DPBF mixtures at 525 nm in air-saturated (a) MeOH and (c) MeCN solutions. Controls with DPBF alone are shown in (b) MeOH and (d) MeCN, respectively. These assays were performed in a 2 mm cuvette, with an absorbance of DATU kept at ∼0.06 at 525 nm.

To further evaluate whether irradiation of DATU generates hydroxyl radicals in an aqueous phosphate buffer at pH 7.4, the ^•^OH-specific fluorescent probe HPF was used.^67–69^ An air-saturated solution containing DATU and HPF was irradiated at 525 nm in phosphate buffer (pH 7.4), and HPF fluorescence (λ_ex_ = 490 nm) was monitored as a function of irradiation time (Figure 12a). A progressive increase in fluorescence intensity is observed, reaching nearly a 6-fold enhancement under a light dose of 16 J cm^–2^ at 525 nm. In contrast, a control experiment performed under identical conditions but in the absence of DATU (i.e., only HPF in phosphate buffer) shows no measurable increase in fluorescence (Figure 12b). These results confirm that DATU generates hydroxyl radicals in aqueous buffer solution under 525 nm excitation, which should contribute as an additional highly cytotoxic ROS to its PDT activity in cells. The ROS measurements presented in this section collectively show that DATU functions as an effective ROS generator. It operates through both type I and type II photosensitization mechanisms across various solvent polarities that are relevant to the different organelles within cells.

**Figure 12:**
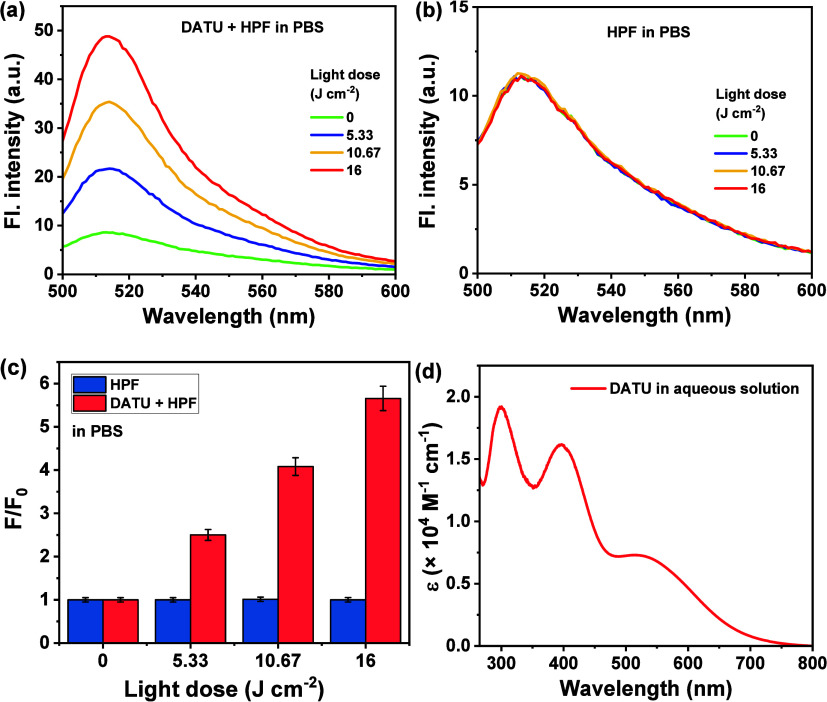
Light dose-dependent changes in HPF fluorescence intensity in aqueous phosphate buffer solution (PBS) upon 525 nm irradiation: (a) in the presence of DATU and (b) in its absence (control). (c) Corresponding plot of *F*/*F*_0_ at the maximum emission as a function of light dose under both conditions. (d) Absorption spectrum of DATU in aqueous buffer solution at pH 7.4 (99:1 PBS/DMSO).

### DATU Acts as an Effective Photosensitizer of 4T1 Murine Mammary Carcinoma Cells upon One-Photon Activation at 525 nm or Two-Photon Activation at 800 nm

To validate the effectiveness of DATU as a PDT agent, we conducted *in vitro* viability assays using 4T1 cells. The *in vitro* photosensitization experiments were performed using both one-photon and two-photon activation of DATU-treated 4T1 cells at 525 and 800 nm, respectively. Figure 13 illustrates the cell viability measurements, which were obtained in triplicates from three independent assays and using two different concentrations of DATU. As shown in Figure 13a, irradiation of 4T1 cells treated with 6 μM DATU resulted in a ∼72% reduction in cell viability using a low-fluence LED lamp of 16 J cm^–2^ at 525 nm, with no detectable dark cytotoxicity. Similarly, irradiation of 4T1 cells treated with 25 μM of DATU resulted in an ∼80% reduction in cell viability upon exposure to a 100 fs, 800 nm laser beam for 30 min (fluence = 40.7 kJ cm^–2^), with no detectable cytotoxicity (Figure 13b). Importantly, 4T1 cells remained viable when irradiated with the same lamp/laser dose at 525 nm/800 nm in the absence of DATU, confirming that the light doses used were not inherently phototoxic (Figure 13a,b). The results presented in Figure 13 unequivocally demonstrate that DATU is a highly effective photosensitizer, capable of targeting 4T1 cancer cells through both one-photon (525 nm) and two-photon (800 nm) excitation. Future work will investigate the mode of action and whether DATU acts as an effective photosensitizer under both normoxic and hypoxic conditions.

**Figure 13:**
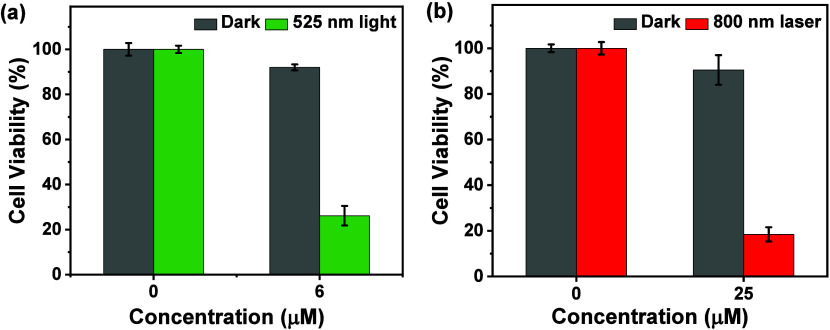
Representative viability studies utilizing 4T1 murine mammary carcinoma cells in the presence and absence of light irradiation: (a) 6 μM DATU with one-photon excitation at 525 nm (16 J cm^–2^) and (b) 25 μM DATU with two-photon excitation using 800 nm laser (40.7 kJ cm^–2^). See the Methods section in the SI for details.

## Conclusions

We have developed a novel nucleoside prodrug analog that can be excited by one and two photons within the optical therapeutic window suitable for deep-tissue PDT applications. We have also established DATU as a potent PDT agent, demonstrating high efficacy against 4T1 murine mammary carcinoma cells upon activation with both one-photon (525 nm) and two-photon (800 nm) excitation. It is shown that both one-photon and two-photon excitations of DATU effectively populate a microsecond-lifetime triplet state and generate reactive oxygen species (^1^O_2_ and ^•^OH) in significantly high yields. Hence, DATU acts through both type I and type II photosensitization mechanisms. Notably, the thionation of DAU resulted in the development of the highest two-photon absorption cross-section nucleoside reported among all nucleobase analogs to date. This advancement marks a significant step forward in the design of nucleoside prodrugs for phototherapeutic applications. Given the expected intracellular behavior of DATU as a prodrug—specifically, the potential enzymatic hydrolysis of the benzoyl esters to produce the deprotected ribose form—we anticipate that the ribose nucleoside of DATU should maintain the photophysical properties of its benzoyl-protected counterpart. This is because the primary chromophore is the π-extended 6-azauridine moiety, not the sugar itself. This hypothesis is further supported by DFT and TDDFT calculations presented in the Supporting Information (Figures S18 and S19).

Another key finding is the first experimental demonstration that a thiobase analog exhibits a 3-fold larger two-photon absorption cross section compared to its carbonyl-containing counterpart under the same experimental conditions. This supports theoretical predictions that thiocarbonyl groups, despite sulfur’s lower electronegativity, can function as better electron acceptors than carbonyl groups. This characteristic is currently attributed to the contribution of the C=S group, which favors a more effective donor−π–acceptor architecture. This new understanding lays the groundwork for the development of other thionated compounds with enhanced two-photon absorption properties for a broad range of applications.

Two-photon-absorption active photosensitizers are currently being used in various fields, including bioimaging,^70^ photocatalysis,^71,72^ and deep-tissue PDT.^73–75^ Our approach highlights the broader potential of structurally engineered thiocarbonyl organic compounds as next-generation photosensitizers.

## Supplementary Material



## Notes

Experimental methods and the synthesis procedure; additional steady-state spectroscopy data; tables, figures, and Cartesian coordinates from quantum-chemical calculations; additional femtosecond- and picosecond-to-microsecond transient absorption spectroscopy data; and ^1^H NMR, ^13^C NMR, and ESI-MS spectra of the synthesized compounds (PDF)
