# Supplementary material for: Development of a Nucleoside Photosensitizer Efficiently Activated by One- or Two-Photon Absorption in the Optical Therapeutic Window
Source: J Am Chem Soc. 2026 Jun 8;148(24):24695–710. doi: 10.1021/jacs.6c00216 (PMC13307366; doi:10.1021/jacs.6c00216)
Supplement: Supplementary file 1 [file ja6c00216_si_001.pdf]

Development of a Nucleoside Photosensitizer Efficiently Activated by One- or Two-Photon Absorption in the Optical Therapeutic Window

Sourav Kanti Seth,[1] Sean J. Hoehn,[1] Chris Acquah,[1] Liraz Levi,[2,3] and Carlos E. Crespo-Hernández[1,*]

[1] Department of Chemistry, Case Western Reserve University, Cleveland, Ohio 44106, USA

[2] Department of Pediatrics, Case Western Reserve University School of Medicine, Cleveland, Ohio 44106, USA

[3] Celloram Inc., Cleveland, Ohio 44106, USA

Supporting Information

1. Experimental

1.1. Materials. 2-Thiopheneglyoxylic acid, semicarbazide hydrochloride, *N*,*O*-bis(trimethylsilyl)acetamide, dry acetonitrile, trimethylsilyl trifluoromethanesulfonate, 1,2-dichloroethane, bromine, sodium thiosulfate, sodium carbonate, palladium acetate, ammonium chloride, anhydrous toluene, Lawesson's reagent, quinine sulfate, pyrrole, benzaldehyde, and 1,3-diphenylisobenzofuran were procured from Sigma-Aldrich. 1-*O*-acetyl-2,3,5-tri-*O*-benzoyl-β-D-ribofuranose, tris(3-sulfophenyl)phosphine trisodium salt, and 4-(dimethylamino)phenylboronic acid were purchased from Tokyo Chemical Industry (TCI). Hydroxyphenyl fluorescein was procured from Invitrogen (Thermo Fisher Scientific). Sodium hydroxide, hydrochloric acid, and glacial acetic acid were of analytical grade. Solvents used for synthesis were of analytical grade, and 1,4-dioxane and benzene were of spectrophotometric grade for spectroscopic measurements. All solvents were air-equilibrated unless otherwise specified. All the reagents were used for synthesis as received.

1.2. Methods

1.2.1. Steady-state measurements. UV–vis absorption and fluorescence emission spectra were recorded using a JASCO V-730 spectrophotometer and an Agilent Cary Eclipse spectrofluorimeter, respectively. The spectrofluorimeter lacks correction for emission intensity above ca. 600 nm, potentially slightly underestimating the intensity in this wavelength range. Molar absorption coefficients (ε) were determined according to Beer–Lambert's Law and represent the mean values from three independent experiments. The fluorescence quantum yields (Φ_f) were measured with quinine sulfate as the standard ($\Phi_f = 0.577$ at 350 nm excitation) following a standard protocol.[1]

1.2.2. Quantum-chemical calculations details. All geometry optimizations, vertical excitation energy (VEE) calculations, and spin–orbit coupling (SOC) analyses were performed using ORCA 5.0.2.[2] Ground-state geometries were optimized using the density functional theory (DFT) formalism employing the B3LYP$_G$-D3BJ[3,4] functional and the def2-TZVPD basis set.[5] Solvent effects (1,4-dioxane and benzene) were modeled using the conductor-like polarizable continuum model (CPCM).[6] Frequency calculations, carried out at the optimized ground-state geometries, confirmed the absence of imaginary frequencies, validating that the structures correspond to local minima.

VEEs were computed using the time-dependent implementation of DFT (TD-DFT) and the M062X-D3ZERO, CAM-B3LYP-D3BJ, X3LYP, PBE0-D3BJ, and B3LYP-D3BJ functionals, with the def2-TZVPD basis set at the optimized ground-state geometries described above. Spin–orbit coupling (SOC) constants between singlet and triplet states were computed using TD-DFT combined with quasi-degenerate perturbation theory, also based on the optimized S_0 geometries.[7] The simulated excited-state absorption spectra (EAS) or the simulated transient absorption spectra were obtained for the first 100 states using the $S_{1(min)}$, $S_{2(min)}$, and $T_{1(min)}$ geometries for DATU, and using the $S_{1(min)}$ geometry of DAU in 1,4-dioxane. The computed EAS of the optimized structures were visualized using Gabedit with a FWHM of 40 nm.[8] To enhance computational efficiency

without significant loss of accuracy, all ground- and excited-state calculations employed the RIJCOSX approximation (a split-resolution of identity-j with chain-of-spheres exchange).[9]

1.2.3. Femtosecond and picosecond-to-microsecond transient absorption spectroscopy. Pump–probe time-resolved transient absorption spectroscopy with femtosecond time resolution was carried out using an amplified laser system (Libra-HE, Coherent, Inc.; 800 nm, 100 fs, 4.0 W, 1 kHz) to generate the pump pulses.[10] A 520 nm excitation pulse was generated using a TOPAS system as previously described.[11] Generation of the 400 nm excitation pulse was done as described previously[10] by pumping an optical rail kit (FKE series, EKSMA optics). A 2-mm translating CaF_2 crystal was used to generate the white-light continuum, providing a probe spectral range of 320–700 nm and a probe window of ca. 2.4 ns. For recording transient absorption spectra on the picosecond-to-microsecond timescale, another white-light (probe) source was used, described in detail elsewhere.[11] Briefly, broadband probe pulses were generated using a photonic crystal fiber (EOS, Ultrafast Systems, LLC). This electronically triggered source provides a time resolution of approximately 400 ps at a 2 kHz repetition rate and a probe wavelength range of ~375–800 nm. Solutions of DATU and DAU were measured in N_2-saturated (15-min purge) and air-equilibrated 1,4-dioxane and benzene, and DATU was additionally recorded under O_2-saturated conditions after a 15-min purge. Transient absorption spectra were recorded over 250 time points spanning 10 or 20 μs. All measurements were conducted in a 2 mm pathlength fused-silica cuvette with continuous stirring using a Teflon-coated magnetic stir bar. Sample degradation was kept below 7%, as confirmed by comparing UV–vis absorption spectra (at their respective absorption maxima) taken before and after laser exposure.

Data acquisition was performed using a custom LabView program, and the resulting datasets were analyzed using global and target analysis via the Glotaran 1.5.1 graphical interface to the TIMP package in R.[12] Time zero was defined as the point of maximum amplitude of the solvent stimulated Raman signal in the femtosecond transient absorption measurements, and as the time at which the transient absorption signal reached its maximum during the initial spectral evolution in the picosecond-to-microsecond transient absorption measurements. Sequential kinetic models were used to fit the data for DATU and DAU in 1,4-dioxane and benzene. All measurements were repeated in triplicate on separate days under identical conditions to ensure reproducibility.

1.2.4. Two-photon absorption cross-section measurements. Two-photon absorption cross-sections were determined using the same femtosecond pump–probe transient absorption setup described above. For one-photon excitation, pump pulses at 400 nm were generated via second-harmonic generation, whereas two-photon excitation was achieved by direct use of the fundamental 800 nm output. All other experimental parameters, including probe generation, sample handling, and data acquisition, were identical to those employed for the fs-transient absorption measurements. Notably, in contrast to the one-photon transient absorption experiments, no significant photodegradation (<1%) was observed under 800 nm two-photon excitation, likely due to the smaller effective absorption cross-section under two-photon conditions compared to one-photon excitation.

Two-photon absorption cross-sections of DATU and DAU in 1,4-dioxane were measured following the reported procedure,[13] using Rhodamine 6G in MeOH as the standard[14] and employing the following equation:

$$\delta_S = \delta_R \left(\frac{\Delta A_S}{\Delta A_R}\right)\left(\frac{\sigma_R^{ex} - \sigma_R^{gr}}{\sigma_S^{ex} - \sigma_S^{gr}}\right)\left(\frac{c_R}{c_S}\right)$$

where, S and R denote the sample and reference, respectively; δ represents the two-photon absorption cross-sections; ΔA is the change in absorbance measured at the peak of the transient absorption spectrum following 800 nm two-photon excitation; σ^{ex} is the one-photon absorption cross-section of the excited state at λ_{max}, the wavelength corresponding to the maximum absorbance in the transient absorption spectrum; σ^{gr} is the one-photon absorption cross-section of the ground state at the same wavelength; c is the concentration of the sample used in the two-photon excitation experiment. The difference between the excited and ground state one-photon absorption cross-section, ($\sigma^{ex} - \sigma^{gr}$), is proportional to the ΔA after one-photon excitation and thus corresponds to the maximum positive ΔA value recorded upon 400 nm one-photon excitation. For all three compounds—DATU, DAU, and Rhodamine 6G—the ΔA values were extracted from their transient absorption spectra at a 20 ps delay, a time point representative of the singlet state population for DAU and Rhodamine 6G, and a combined singlet and triplet state for DATU, as shown in the main text.

1.2.5. Singlet oxygen quantum yields measurements. The singlet oxygen quantum yield (Φ_Δ) was measured using an indirect method involving 1,3-diphenylisobenzofuran (DPBF)[15–18] as the $^1O_2$ probe and meso-tetraphenylporphyrin (TPP) as the reference standard (Φ_Δ = 0.62 in benzene at 355 nm).[19,20] The absorbance of DATU was adjusted to approximately 0.2 at the excitation wavelength of 355 nm in benzene, using a 1 cm path-length quartz cuvette. The spectrophotometer baseline was then corrected using this photosensitizer solution, such that its absorbance was set to zero over the spectral range. Subsequently, DPBF was added, and its absorbance was adjusted to ~1.0 at 415 nm. This procedure ensured that only the absorption of DPBF—and its time-dependent decrease upon irradiation—was monitored, rather than the combined absorbance of DPBF and the photosensitizer, thereby improving the accuracy of absorbance changes associated with DPBF degradation. The solution was irradiated with a laser beam at 355 nm for different time intervals, and the absorption spectra were recorded. The same procedure was applied to the reference, TPP. Subsequently, the Φ_Δ of DATU was determined using the following equation:[16–18]

$$\Phi_{\Delta S} = \Phi_{\Delta R}\left(\frac{m_S}{m_R}\right)\left(\frac{F_R}{F_S}\right)$$

where S and R denote the sample and the reference, respectively, Φ_Δ is the quantum yield of $^1O_2$ generation, m is the slope of (A/A_0) at 315 nm vs. irradiation time plot, and F is the absorption correction factor calculated as ($1 - 10^{-OD}$), where OD is the optical density at the excitation wavelength. It is important to note that DPBF evidences some degree of degradation (ca. 13%) by

self-photolysis when excited at 355 nm. However, this effect is significantly less than its reactivity toward singlet oxygen ($^1O_2$). Since photolysis occurs in both the sample and the reference, its impact is effectively cancelled in the calculation of Φ_Δ.

To demonstrate DATU's ability to generate $^1O_2$ under two-photon excitation, DPBF degradation (OD ~1 at its maximum) was monitored in air-equilibrated benzene in the absence and the presence of DATU (OD ~0.2 at 400 nm) upon irradiation with 0.8 W of 800 nm (100 fs pulses; beam diameter 2.6 ± 0.1 mm).

1.2.6. Cell viability assays under one- and two-photon excitation. Murine mammary carcinoma (4T1) cells were cultured in complete ATCC-formulated RPMI-1640 medium supplemented with 10% fetal bovine serum (FBS) and 1% penicillin/streptomycin. All cell lines were maintained under standard conditions at 37 °C in a humidified incubator with 5% CO_2. For cell viability assays, 5000 cells/well were seeded in 96-well plates and allowed to adhere overnight. The following day, cells were treated with DATU (prepared from a DMSO stock; final DMSO concentration ~1% v/v) for 48 h at 37 °C. Control groups were treated with ~1% DMSO (vehicle). Prior to light exposure, cells were washed three times with phosphate-buffered saline (PBS), and fresh medium was added. For one-photon excitation, cells were irradiated for 60 minutes using a 525 nm LED source (FWHM = 20 nm; total fluence = 16 J cm^{-2}) with a custom-built setup as described previously.[21] For two-photon excitation, cells were irradiated for 30 min using an 800 nm fs-pulsed laser (1.2 W, beam diameter = 2.6 ± 0.1 mm; fluence = 40.7 kJ cm^{-2}) from an amplified laser system (Libra-HE, Coherent; 100 fs pulse duration, 1 kHz repetition rate). After irradiation, cells were incubated overnight before cell viability was assessed using the PrestoBlue Cell Viability Reagent (Invitrogen).[22] Fluorescence (λ_{exc} = 560 nm / λ_{em} = 590 nm) was measured using a SpectraMax i3X plate reader (Molecular Devices) and quantified via SoftMax Pro 6 software. The reported results represent the mean of three replicates with one standard deviation as the error.

2. Supporting results and notes

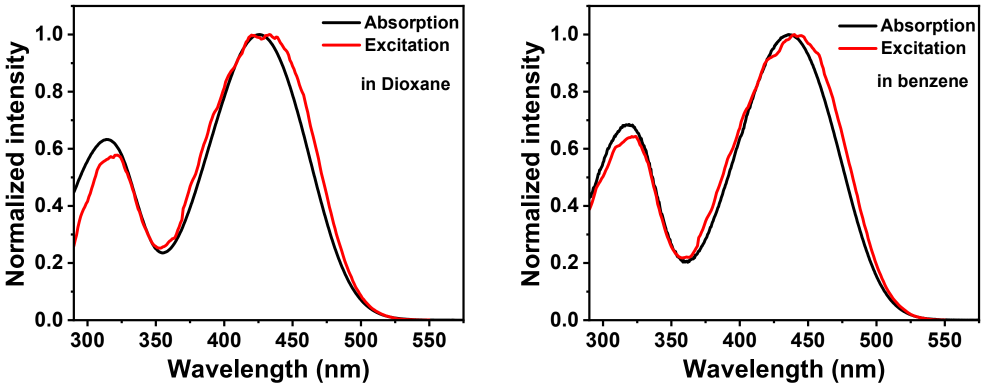

Figure S1. Comparison of experimental absorption and excitation spectra of DAU in (a) 1,4-dioxane and (b) benzene.

2.1. Quantum-chemical calculations

Ground-state (S_0) optimized geometries of DATU and DAU, including *syn*- and *anti*-rotamers ($DATU_{syn}$, $DATU_{anti}$, DAU_{syn}, and DAU_{anti}), computed in 1,4-dioxane and benzene.

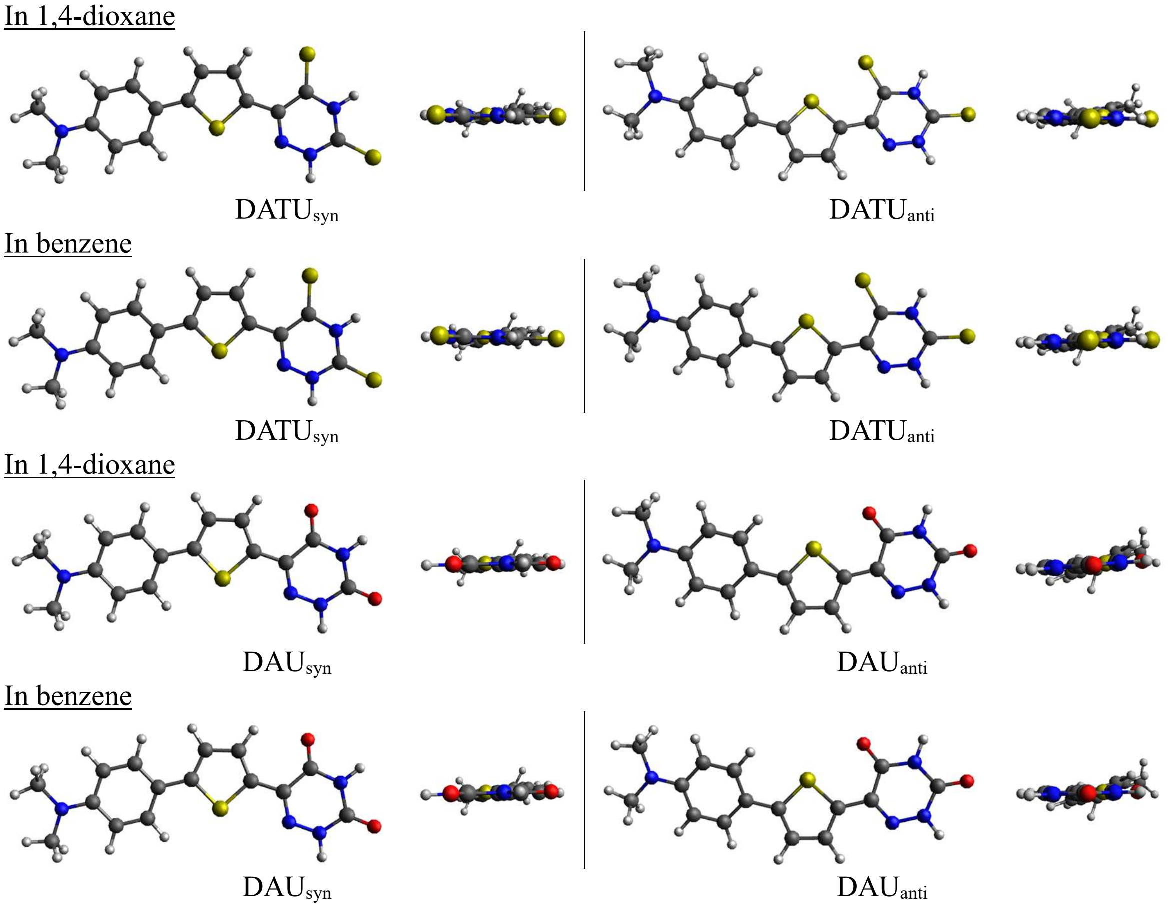

Figure S2. Ground-state (S_0) optimized geometries of $DATU_{syn}$, $DATU_{anti}$, DAU_{syn}, and DAU_{anti} in 1,4-dioxane and benzene.

To select the most suitable functional(s) that best model the experimental absorption spectra, simulated spectra were obtained from VEE calculations at the optimized S_0 geometries for both *syn*- and *anti*-rotamers in 1,4-dioxane for both DATU and DAU. M062X, CAM-B3LYP, X3LYP, PBE0, and B3LYP functionals were tested, and the simulated spectra and the order of the energy levels and electronic characters were observed to be very similar for M062X and CAM-B3LYP, but different from the X3LYP, PBE0, and B3LYP functionals, which were very similar to each other. In addition, both rotamers show very similar results at each of the tested functionals. Therefore, only results for the *syn*-rotamers (major) are presented and discussed here in Figures S3 and S4.

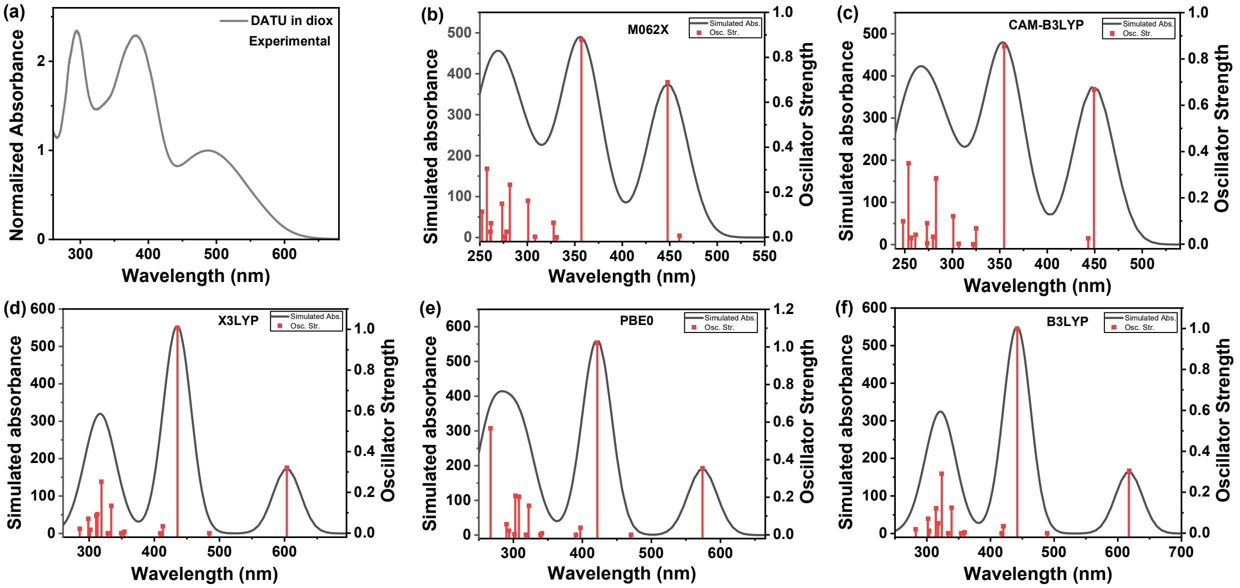

Figure S3. Comparison of (a) experimental and (b-f) simulated absorption spectra and corresponding oscillator strengths for $DATU_{syn}$ in 1,4-dioxane using (b) M062X, (c) CAM-B3LYP, (d) X3LYP, (e) PBE0, and (f) B3LYP functionals.

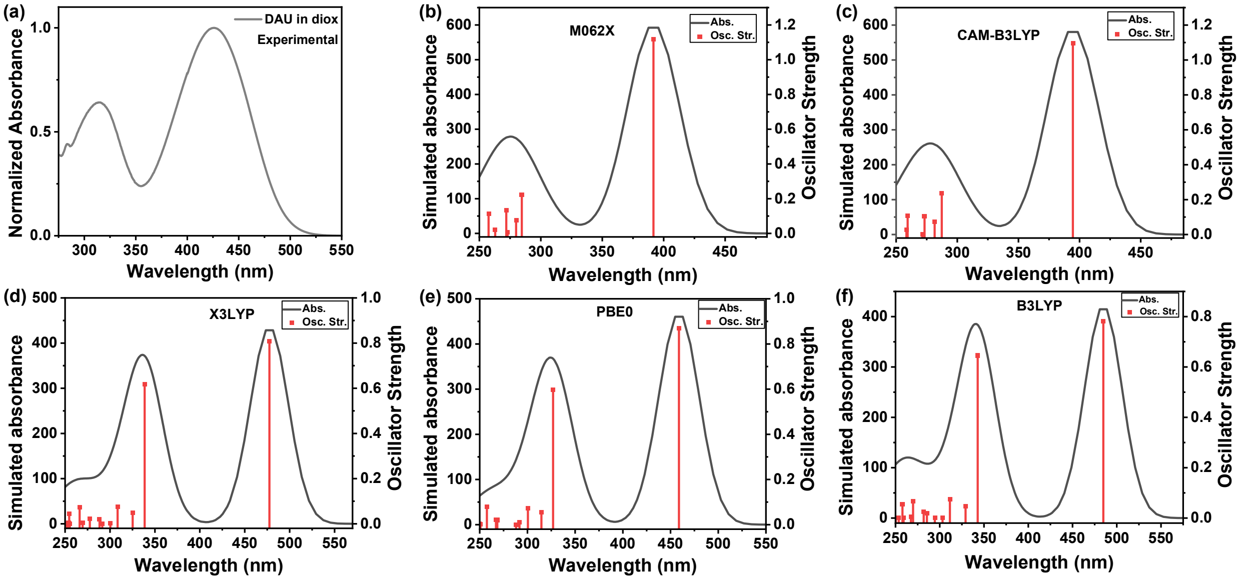

Figure S4. Comparison of (a) experimental and (b-f) simulated absorption spectra and corresponding oscillator strengths for DAU_{syn} in 1,4-dioxane using (b) M062X, (c) CAM-B3LYP, (d) X3LYP, (e) PBE0, and (f) B3LYP functionals.

On the one hand, the vertical S_1 and S_2 are nearly isoenergetic (< 0.1 eV) within the accuracy of the calculations when using the M062X and CAM-B3LYP functionals for DATU (Tables S1 to S4), with the nπ* and ππ* characters reversed. On the other hand, the S_2(nπ*) state is ca. 0.5 eV higher in energy than S_1(ππ*) state when using the X3LYP, PBE0, and B3LYP functionals (Table S5). Regardless of the functional used, the Kohn-Sham orbitals are very similar for all these tested functionals, exhibiting very similar charge-transfer character between the HOMO to LUMO transitions (Figures S5 and S6). As shown in Figure S3, the VEEs and oscillator strengths obtained using the M062X and CAM-B3LYP functionals, more satisfactory model the experimental absorption spectra compared to the X3LYP, PBE0, and B3LYP functionals. Therefore, we decided to use the M062X and CAM-B3LYP functionals for discussing the experimental results in the main text and for the calculations of the SOCs for both rotamers of both compounds in 1,4-dioxane and benzene, reported in Tables S6 to S9 below.

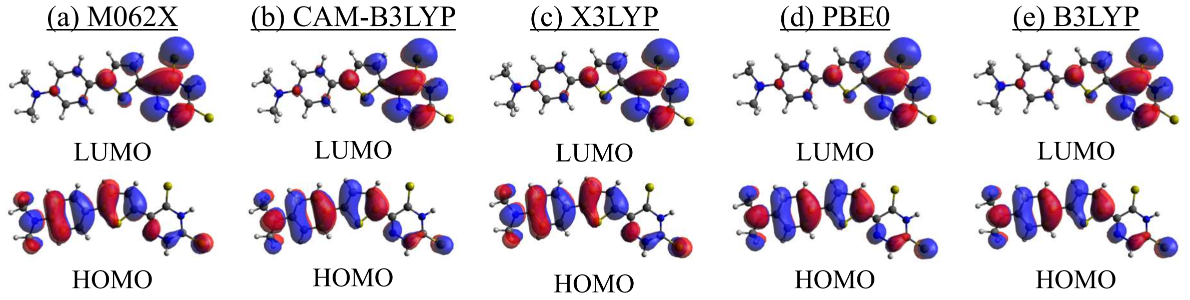

Figure S5. Comparison of Kohn-Sham orbitals for HOMO and LUMO of $DATU_{syn}$ in 1,4-dioxane using (a) M062X, (b) CAM-B3LYP, (c) X3LYP, (d) PBE0, and (e) B3LYP functionals.

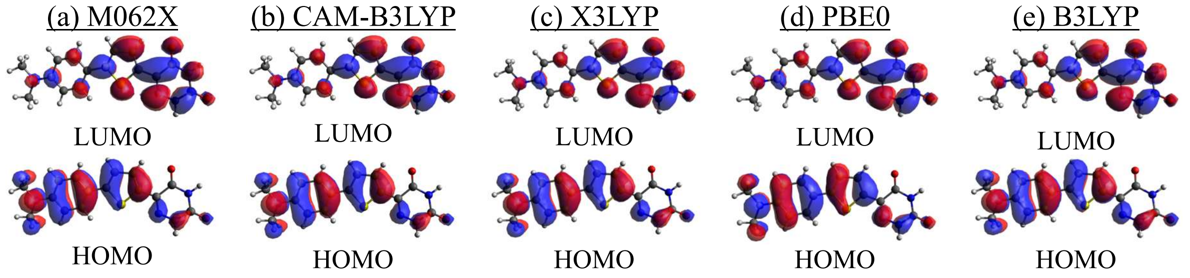

Figure S6. Comparison of Kohn-Sham orbitals for HOMO and LUMO of DAU_{syn} in 1,4-dioxane using (a) M062X, (b) CAM-B3LYP, (c) X3LYP, (d) PBE0, and (e) B3LYP functionals.

Vertical excitation energies (VEEs) and corresponding spin-orbit coupling constants (SOCs) using S_0 geometries for DATU.

VEEs in dioxane

Table S1. VEEs for $DATU_{syn}$

State	M062X			CAM-B3LYP		
	Energy/eV (f, Osc. Str.)	Transition (% contribution)	Electronic character	Energy/eV (f, Osc. Str.)	Transition (% contribution)	Electronic character
S_1	2.70 (0.0054)	H-2 → L (42)<br>H-3 → L (36)	nπ*<br>nπ*	2.76 (0.6686)	H → L (81)	ππ*(CT)
S_2	2.77 (0.6899)	H → L (86)	ππ*(CT)	2.80 (0.0266)	H-2 → L (45)<br>H-3 → L (34)	nπ*<br>nπ*
S_3	3.47 (0.8768)	H → L+1 (81)	ππ*(LE+CT)	3.50 (0.8554)	H → L+1 (76)	ππ*(LE+CT)
T_1	1.97	H → L (60)<br>H-1 → L (21)	ππ*(CT)<br>ππ*(LE+CT)	1.83	H → L (55)<br>H-1 → L (24)	ππ*(CT)<br>ππ*(LE+CT)
T_2	2.42	H-2 → L (39)<br>H-3 → L (37)<br>H-2 → L+1 (10)	nπ*<br>nπ*<br>nπ*	2.48	H-2 → L (43)<br>H-3 → L (36)	nπ*<br>nπ*
T_3	2.61	H-4 → L (29)<br>H-7 → L (18)<br>H → L+1 (19)	ππ*<br>ππ*<br>ππ*(LE+CT)	2.52	H-4 → L (24)<br>H-7 → L (21)<br>H → L+1 (19)	ππ*<br>ππ*<br>ππ*(LE+CT)
T_4	3.00	H → L+1 (37)<br>H-4 → L (16)<br>H-1 → L (10)	ππ*(LE+CT)<br>ππ*<br>ππ*(LE+CT)	2.92	H → L+1 (28)<br>H-4 → L (18)<br>H → L+2 (13)<br>H-1 → L (11)	ππ*(LE+CT)<br>ππ*<br>ππ*<br>ππ*(LE+CT)
T_5	3.17	H-1 → L+1 (33)<br>H → L+2 (16)<br>H-1 → L (16)	ππ*(LE+CT)<br>ππ*<br>ππ*(LE+CT)	3.15	H-1 → L+1 (37)<br>H → L+2 (13)	ππ*<br>ππ*
T_6	3.56	H-2 → L+1 (37)<br>H-3 → L+1 (22)<br>H-2 → L+2 (12)	nπ*<br>nπ*<br>nπ*	3.61	H-1 → L (18)<br>H-5 → L (17)<br>H → L+5 (12)<br>H → L+2 (11)	ππ*<br>ππ*<br>ππ*<br>ππ*
T_7	3.65	H-1 → L (27)<br>H → L (14)<br>H-4 → L+1 (13)<br>H → L+2 (12)	ππ*(LE+CT)<br>ππ*(CT)<br>ππ*<br>ππ*	3.64	H-2 → L+1 (34)<br>H-3 → L+1 (23)<br>H-2 → L+2 (12)	nπ*<br>nπ*<br>nπ*
T_8				3.65	H → L+5 (63)	ππ*

Table S2. VEEs for $DATU_{anti}$

State	M062X			CAM-B3LYP		
	Energy / eV (f, Osc. Str.)	Transition (% contribution)	Electronic character	Energy / eV (f, Osc. Str.)	Transition (% contribution)	Electronic character
S_1	2.68 (0.1260)	H-2 → L (43)<br>H-3 → L (21)<br>H → L (19)	nπ*<br>nπ*<br>ππ*(CT)	2.70 (0.6622)	H → L (84)	ππ*(CT)
S_2	2.72 (0.5405)	H → L (70)<br>H-2 → L (11)	ππ*(CT)<br>nπ*	2.80 (0.0166)	H-2 → L (57)<br>H-3 → L (24)	nπ*<br>nπ*
S_3	3.46 (0.8478)	H → L+1 (83)	ππ*(LE+CT)	3.49 (0.8124)	H → L+1 (79)	ππ*(LE+CT)
T_1	1.89	H → L (62)	ππ*(CT)	1.75	H → L (57)	ππ*(CT)

		H-1 → L (21)	ππ*(LE+CT)		H-1 → L (24)	ππ*(LE+CT)
T_2	2.42	H-2 → L (50)<br>H-3 → L (28)<br>H-2 → L+1 (11)	nπ*<br>nπ*<br>nπ*	2.49	H-2 → L (54)<br>H-3 → L (26)<br>H-2 → L+1 (10)	nπ*<br>nπ*<br>nπ*
T_3	2.62	H-4 → L (24)<br>H-7 → L (20)<br>H → L+1 (25)	ππ*<br>ππ*<br>ππ*(LE+CT)	2.54	H-4 → L (20)<br>H-7 → L (22)<br>H → L+1 (25)	ππ*<br>ππ*<br>ππ*(LE+CT)
T_4	3.01	H → L+1 (33)<br>H-4 → L (20)	ππ*(LE+CT)<br>ππ*	2.94	H → L+1 (23)<br>H-4 → L (21)<br>H → L+2 (15)	ππ*(LE+CT)<br>ππ*<br>ππ*
T_5	3.17	H-1 → L+1 (30)<br>H → L+2 (19)<br>H-1 → L (16)	ππ*(LE+CT)<br>ππ*<br>ππ*(LE+CT)	3.15	H-1 → L+1 (34)<br>H → L+2 (15)	ππ*<br>ππ*
T_6	3.57	H-2 → L+1 (29)<br>H-3 → L+1 (30)	nπ*<br>nπ*	3.59	H-1 → L (18)<br>H → L+5 (30)<br>H → L+2 (12)	ππ*<br>ππ*<br>ππ*
T_7	3.62	H-1 → L (31)<br>H → L (14)<br>H → L+2 (15)	ππ*(LE+CT)<br>ππ*(CT)<br>ππ*	3.64	H-2 → L+1 (26)<br>H-3 → L+1 (30)<br>H-3 → L+2 (10)	nπ*<br>nπ*<br>nπ*
T_8				3.65	H → L+5 (35)<br>H-1 → L (13)<br>H → L (11)	ππ*<br>ππ*(LE+CT)<br>ππ*(CT)

VEEs in benzene

Table S3. VEEs for $DATU_{syn}$

State	M062X			CAM-B3LYP		
	Energy / eV<br>(*f*, Osc. Str.)	Transition<br>(% contribution)	Electronic<br>character	Energy / eV<br>(*f*, Osc. Str.)	Transition<br>(% contribution)	Electronic<br>character
S_1	2.69<br>(0.0070)	H-2 → L (42)<br>H-3 → L (36)	nπ*<br>nπ*	2.75<br>(0.6936)	H → L (81)	ππ*(CT)
S_2	2.76<br>(0.7068)	H → L (85)	ππ*(CT)	2.80<br>(0.0183)	H-2 → L (46)<br>H-3 → L (34)	nπ*<br>nπ*
S_3	3.47<br>(0.8727)	H → L+1 (81)	ππ*(LE+CT)	3.49<br>(0.8517)	H → L+1 (76)	ππ*(LE+CT)
T_1	1.97	H → L (60)<br>H-1 → L (21)	ππ*(CT)<br>ππ*(LE+CT)	1.83	H → L (55)<br>H-1 → L (24)	ππ*(CT)<br>ππ*(LE+CT)
T_2	2.42	H-2 → L (39)<br>H-3 → L (37)<br>H-2 → L+1 (10)	nπ*<br>nπ*<br>nπ*	2.48	H-2 → L (43)<br>H-3 → L (36)	nπ*<br>nπ*
T_3	2.61	H-4 → L (29)<br>H-7 → L (18)<br>H → L+1 (19)	ππ*<br>ππ*<br>ππ*(LE+CT)	2.52	H-4 → L (24)<br>H-7 → L (21)<br>H → L+1 (19)	ππ*<br>ππ*<br>ππ*(LE+CT)
T_4	3.00	H → L+1 (37)<br>H-4 → L (16)<br>H-1 → L (10)	ππ*(LE+CT)<br>ππ*<br>ππ*(LE+CT)	2.92	H → L+1 (28)<br>H-4 → L (18)<br>H → L+2 (13)<br>H-1 → L (12)	ππ*(LE+CT)<br>ππ*<br>ππ*<br>ππ*(LE+CT)
T_5	3.17	H-1 → L+1 (33)<br>H → L+2 (16)<br>H-1 → L (16)	ππ*(LE+CT)<br>ππ*<br>ππ*(LE+CT)	3.15	H-1 → L+1 (37)<br>H → L+2 (13)	ππ*<br>ππ*
T_6	3.56	H-2 → L+1 (37)<br>H-3 → L+1 (22)<br>H-2 → L+2 (12)	nπ*<br>nπ*<br>nπ*	3.61	H-1 → L (18)<br>H-5 → L (17)<br>H → L+5 (12)<br>H → L+2 (11)	ππ*(LE+CT)<br>ππ*(LE+CT)<br>ππ*<br>ππ*

T_7	3.65	H-1 → L (27)<br>H → L (14)<br>H-4 → L+1 (13)<br>H → L+2 (12)	ππ*(LE+CT)<br>ππ*(CT)<br>ππ*<br>ππ*	3.64	H-2 → L+1 (34)<br>H-3 → L+1 (23)<br>H-2 → L+2 (12)	nπ*<br>nπ*<br>nπ*
T_8				3.65	H → L+5 (62)	ππ*

Table S4. VEEs for $DATU_{anti}$

State	M062X			CAM-B3LYP		
	Energy / eV (*f*, Osc. Str.)	Transition (% contribution)	Electronic character	Energy / eV (*f*, Osc. Str.)	Transition (% contribution)	Electronic character
S_1	2.68<br>(0.1914)	H-2 → L (38)<br>H-3 → L (19)<br>H → L (27)	nπ*<br>nπ*<br>ππ*(CT)	2.70<br>(0.6813)	H → L (84)	ππ*(CT)
S_2	2.71<br>(0.4944)	H → L (61)<br>H-2 → L (16)	ππ*(CT)<br>nπ*	2.80<br>(0.0154)	H-2 → L (58)<br>H-3 → L (24)	nπ*<br>nπ*
S_3	3.46<br>(0.8446)	H → L+1 (83)	ππ*(LE+CT)	3.48<br>(0.8088)	H → L+1 (78)	ππ*(LE+CT)
T_1	1.89	H → L (62)<br>H-1 → L (21)	ππ*(CT)<br>ππ*(LE+CT)	1.75	H → L (57)<br>H-1 → L (24)	ππ*(CT)<br>ππ*(LE+CT)
T_2	2.42	H-2 → L (50)<br>H-3 → L (28)<br>H-2 → L+1 (11)	nπ*<br>nπ*<br>nπ*	2.49	H-2 → L (54)<br>H-3 → L (26)<br>H-2 → L+1 (10)	nπ*<br>nπ*<br>nπ*
T_3	2.62	H-4 → L (25)<br>H-7 → L (20)<br>H → L+1 (25)	ππ*<br>ππ*<br>ππ*(LE+CT)	2.54	H-4 → L (20)<br>H-7 → L (22)<br>H → L+1 (25)	ππ*<br>ππ*<br>ππ*(LE+CT)
T_4	3.01	H → L+1 (33)<br>H-4 → L (20)	ππ*(LE+CT)<br>ππ*	2.94	H → L+1 (23)<br>H-4 → L (21)<br>H → L+2 (15)	ππ*(LE+CT)<br>ππ*<br>ππ*
T_5	3.17	H-1 → L+1 (30)<br>H → L+2 (19)<br>H-1 → L (16)	ππ*(LE+CT)<br>ππ*<br>ππ*(LE+CT)	3.15	H-1 → L+1 (34)<br>H → L+2 (15)	ππ*<br>ππ*
T_6	3.57	H-2 → L+1 (29)<br>H-3 → L+1 (30)	nπ*<br>nπ*	3.59	H-1 → L (18)<br>H → L+5 (30)<br>H → L+2 (12)	ππ*(LE+CT)<br>ππ*<br>ππ*
T_7	3.62	H-1 → L (31)<br>H → L (14)<br>H → L+2 (15)	ππ*(LE+CT)<br>ππ*(CT)<br>ππ*	3.65	H-2 → L+1 (26)<br>H-3 → L+1 (30)<br>H-3 → L+2 (10)	nπ*<br>nπ*<br>nπ*
T_8				3.65	H → L+5 (35)<br>H-1 → L (13)<br>H → L (11)	ππ*<br>ππ*(LE+CT)<br>ππ*(CT)

Table S5. VEEs of $DATU_{syn}$ in 1,4-dioxane using B3LYP, X3LYP and PBE0 functionals

State	B3LYP			X3LYP			PBE0		
	Energy/eV (*f*, Osc. Str.)	Transition (% contribution)	Electronic character	Energy/eV (*f*, Osc. Str.)	Transition (% contribution)	Electronic character	Energy/eV (*f*, Osc. Str.)	Transition (% contribution)	Electronic character
S_1	2.01<br>(0.3051)	H → L (97)	ππ*(CT)	2.06<br>(0.3215)	H → L (97)	ππ*(CT)	2.16<br>(0.3543)	H → L (97)	ππ*(CT)
S_2	2.53<br>(0.0002)	H-2 → L (74)<br>H-3 → L (22)	nπ*	2.56<br>(0.0002)	H-2 → L (71)<br>H-3 → L (24)	nπ*<br>nπ*	2.64<br>(0.0004)	H-2 → L (68)<br>H-3 → L (25)	nπ*<br>nπ*
T_1	1.48	H → L (86)	ππ*(CT)	1.50	H → L (84)	ππ*(CT)	1.56	H → L (80)<br>H-1 → L (12)	ππ*(CT)<br>ππ*(LE+CT)

T_2	2.26	H-2 → L (62) H-3 → L (30)	nπ* nπ*	2.28	H-2 → L (60) H-3 → L (31)	nπ* nπ*	2.33	H-2 → L (57) H-3 → L (31)	nπ* nπ*

SOCs in 1,4-dioxane

Table S6. SOCs (cm^{-1}) for $DATU_{syn}$

Transition	M062X	CAM-B3LYP
S_1-T_1	41.2	8.27
S_1-T_2	1.6	4.81
S_1-T_3	112.1	22.37
S_1-T_4	66.2	14.78
S_2-T_1	3.7	42.63
S_2-T_2	5.0	3.45
S_2-T_3	10.3	115.8
S_2-T_4	6.4	77.48

Table S7. SOCs (cm^{-1}) for $DATU_{anti}$

Transition	M062X	CAM-B3LYP
S_1-T_1	40.3	6.70
S_1-T_2	2.9	4.45
S_1-T_3	94.0	15.25
S_1-T_4	62.8	12.06
S_2-T_1	20.1	46.27
S_2-T_2	3.3	1.87
S_2-T_3	47.4	109.27
S_2-T_4	30.6	79.90

SOCs in benzene

Table S8. SOCs (cm^{-1}) for $DATU_{syn}$

Transition	M062X	CAM-B3LYP
S_1-T_1	41.2	6.77
S_1-T_2	1.6	4.76
S_1-T_3	112.0	18.21
S_1-T_4	66.2	12.14
S_2-T_1	4.1	42.88
S_2-T_2	4.9	3.16
S_2-T_3	11.6	116.54
S_2-T_4	7.1	78.02

Table S9. SOCs (cm^{-1}) for $DATU_{anti}$

Transition	M062X	CAM-B3LYP
S_1-T_1	37.9	6.30
S_1-T_2	3.3	4.84
S_1-T_3	88.2	14.28
S_1-T_4	59.2	11.45
S_2-T_1	24.4	46.44
S_2-T_2	3.3	1.74
S_2-T_3	57.3	109.39
S_2-T_4	37.2	79.95

VEEs and corresponding SOCs using S_0 geometries for DAU.

VEEs in dioxane

Table S10. VEEs for DAU_{syn}

State	M062X			CAM-B3LYP		
	Energy / eV (*f*, Osc. Str.)	Transition (% contribution)	Electronic character	Energy / eV (*f*, Osc. Str.)	Transition (% contribution)	Electronic character
S_1	3.17 (1.1179)	H → L (91)	ππ*(LE+CT)	3.14 (1.0953)	H → L (88)	ππ*(LE+CT)
S_2	4.36 (0.2216)	H → L+1 (59)<br>H-1 → L (22)	ππ*(LE+CT)<br>ππ*(LE+CT)	4.31 (0.2376)	H → L+1 (61)<br>H-1 → L (21)	ππ*(LE+CT)<br>ππ*(LE+CT)
T_1	2.22	H → L (73)<br>H-1 → L (19)	ππ*(LE+CT)<br>ππ*(LE+CT)	2.04	H → L (66)<br>H-1 → L (23)	ππ*(LE+CT)<br>ππ*(LE+CT)
T_2	3.22	H → L+1 (43)<br>H-1 → L (30)	ππ*(LE+CT)<br>ππ*(LE+CT)	3.05	H → L+1 (44)<br>H-1 → L (24)<br>H-4 → L (11)	ππ*(LE+CT)<br>ππ*(LE+CT)<br>ππ*
T_3	3.74	H → L+4 (87)	ππ*	3.63	H → L+4 (81)	ππ*

Table S11. VEEs for DAU_{anti}

State	M062X			CAM-B3LYP		
	Energy / eV (*f*, Osc. Str.)	Transition (% contribution)	Electronic character	Energy / eV (*f*, Osc. Str.)	Transition (% contribution)	Electronic character
S_1	3.18 (1.0698)	H → L (90)	ππ*(LE+CT)	3.16 (1.0609)	H → L (87)	ππ*(LE+CT)
S_2	4.31 (0.2703)	H → L+1 (78)	ππ*(LE+CT)	4.28 (0.2540)	H → L+1 (76)<br>H-1 → L (11)	ππ*(LE+CT)<br>ππ*(LE+CT)
T_1	2.22	H → L (68)<br>H-1 → L (23)	ππ*(LE+CT)<br>ππ*(LE+CT)	2.04	H → L (62)<br>H-1 → L (27)	ππ*(LE+CT)<br>ππ*(LE+CT)
T_2	3.23	H → L+1 (53)<br>H-1 → L (22)	ππ*(LE+CT)<br>ππ*(LE+CT)	3.06	H → L+1 (52)<br>H-1 → L (17)	ππ*(LE+CT)<br>ππ*(LE+CT)
T_3	3.73	H → L+4 (85)	ππ*	3.63	H → L+4 (55)<br>H → L+3 (26)	ππ*<br>ππ*

VEEs in benzene

Table S12. VEEs for DAU_{syn}

State	M062X			CAM-B3LYP		
	Energy / eV (*f*, Osc. Str.)	Transition (% contribution)	Electronic character	Energy / eV (*f*, Osc. Str.)	Transition (% contribution)	Electronic character
S_1	3.15 (1.1271)	H → L (91)	ππ*(LE+CT)	3.13 (1.1036)	H → L (88)	ππ*(LE+CT)
S_2	4.35 (0.2244)	H → L+1 (59)<br>H-1 → L (23)	ππ*(LE+CT)<br>ππ*(LE+CT)	4.31 (0.2412)	H → L+1 (60)<br>H-1 → L (22)	ππ*(LE+CT)<br>ππ*(LE+CT)
T_1	2.22	H → L (73)<br>H-1 → L (19)	ππ*(LE+CT)<br>ππ*(LE+CT)	2.04	H → L (66)<br>H-1 → L (23)	ππ*(LE+CT)<br>ππ*(LE+CT)
T_2	3.22	H → L+1 (43)<br>H-1 → L (30)	ππ*(LE+CT)<br>ππ*(LE+CT)	3.05	H → L+1 (44)<br>H-1 → L (24)<br>H-4 → L (11)	ππ*(LE+CT)<br>ππ*(LE+CT)<br>ππ*
T_3	3.74	H → L+4 (87)	ππ*	3.63	H → L+4 (78)	ππ*

Table S13. VEEs for DAU_{anti}

State	M062X			CAM-B3LYP		
	Energy / eV (*f*, Osc. Str.)	Transition (% contribution)	Electronic character	Energy / eV (*f*, Osc. Str.)	Transition (% contribution)	Electronic character
S_1	3.17 (1.0857)	H → L (90)	ππ*(LE+CT)	3.14 (1.0748)	H → L (87)	ππ*(LE+CT)
S_2	4.30 (0.2675)	H → L+1 (78)<br>H-1 → L (10)	ππ*(LE+CT)<br>ππ*(LE+CT)	4.27 (0.2525)	H → L+1 (75)<br>H-1 → L (12)	ππ*(LE+CT)<br>ππ*(LE+CT)
T_1	2.22	H → L (68)<br>H-1 → L (23)	ππ*(LE+CT)<br>ππ*(LE+CT)	2.04	H → L (62)<br>H-1 → L (27)	ππ*(LE+CT)<br>ππ*(LE+CT)
T_2	3.22	H → L+1 (53)<br>H-1 → L (22)	ππ*(LE+CT)<br>ππ*(LE+CT)	3.06	H → L+1 (52)<br>H-1 → L (17)	ππ*(LE+CT)<br>ππ*(LE+CT)
T_3	3.73	H → L+4 (85)	ππ*	3.63	H → L+4 (54)<br>H → L+3 (27)	ππ*<br>ππ*

SOCs in 1,4-dioxane

Table S14. SOCs (cm^{-1}) for DAU_{syn}

Transition	M062X	CAM-B3LYP
S_1-T_1	0.15	0.15
S_1-T_2	0.28	0.28

Table S15. SOCs (cm^{-1}) for DAU_{anti}

Transition	M062X	CAM-B3LYP
S_1-T_1	0.15	0.15
S_1-T_2	1.30	1.30

SOCs in benzene

Table S16. SOCs (cm^{-1}) for DAU_{syn}

Transition	M062X	CAM-B3LYP
S_1-T_1	0.19	0.14
S_1-T_2	0.24	0.20

Table S17. SOCs (cm^{-1}) for DAU_{anti}

Transition	M062X	CAM-B3LYP
S_1-T_1	0.13	0.15
S_1-T_2	1.35	1.23

Optimized excited state geometries for DATU$_{syn}$ and DAU$_{syn}$ in 1,4-dioxane and associated simulated excited state absorption spectra.

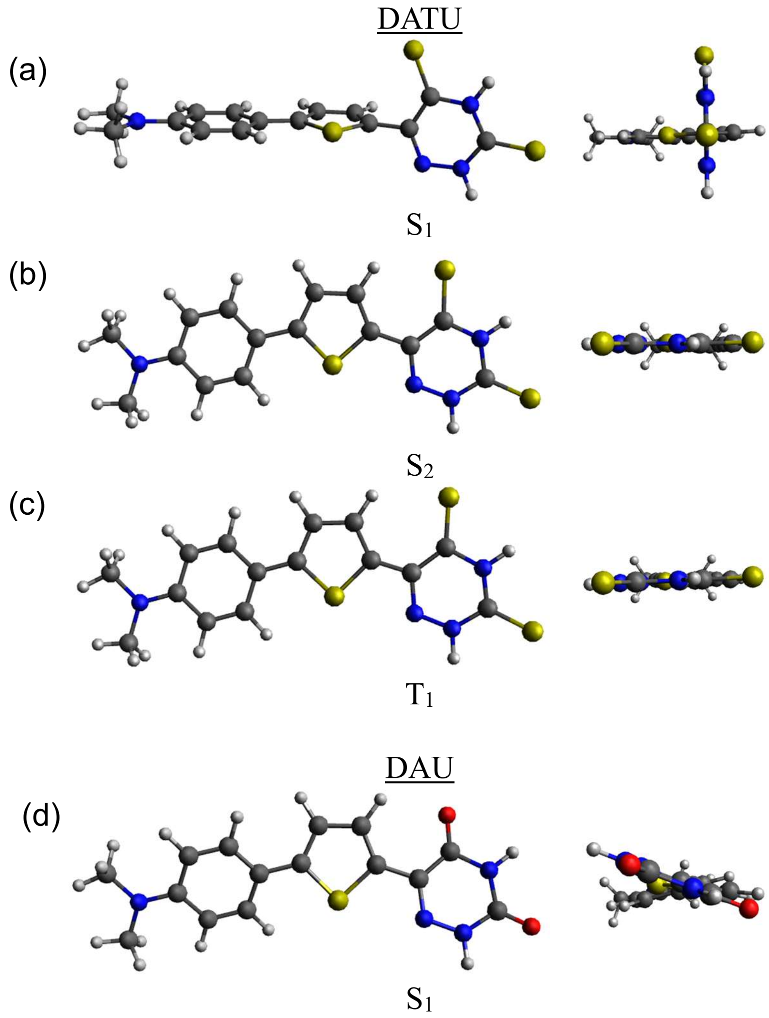

Figure S7. Optimized excited-state geometries: (a) S_1, (b) S_2, and (c) T_1 for DATU$_{syn}$, and (d) S_1 for DAU$_{syn}$ in 1,4-dioxane.

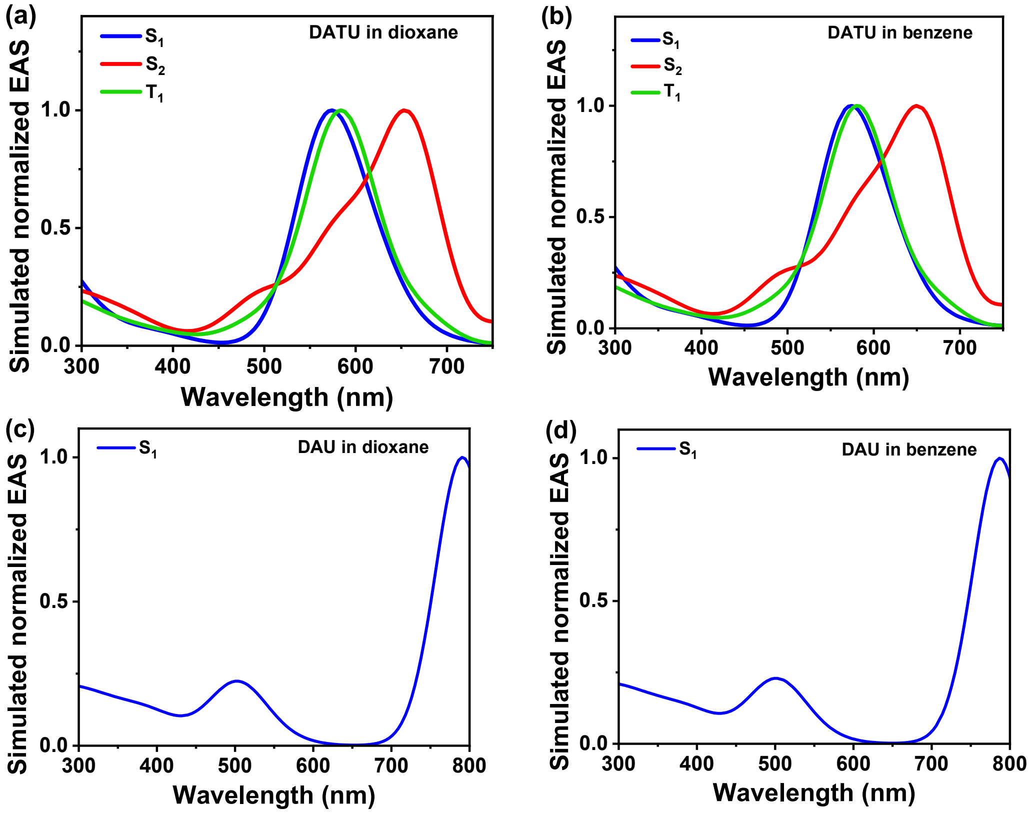

Figure S8. Normalized simulated excited-state absorption (ESA) spectra calculated using optimized S_1, S_2, and T_1 geometries for DATU in (a) 1,4-dioxane and (b) benzene and using the optimized S1 geometry for DAU in (c) 1,4-dioxane and (d) benzene.

Table S18. Frontier orbital (HOMO and LUMO) energies (eV) of DATU and DAU in 1,4-dioxane and benzene using M062X and CAM-B3LYP functionals

Solvent	Functional	Parameter	$DATU_{syn}$	$DATU_{anti}$	DAU_{syn}	DAU_{anti}
1,4-dioxane	M062X	E_{HOMO}	-6.341	-6.276	-6.293	-6.266
		E_{LUMO}	-2.093	-2.090	-1.531	-1.512
		ΔE	4.248	4.186	4.762	4.754
1,4-dioxane	CAM-B3LYP	E_{HOMO}	-6.425	-6.360	-6.372	-6.345
		E_{LUMO}	-1.858	-1.854	-1.293	-1.278
		ΔE	4.567	4.506	5.079	5.067
Benzene	M062X	E_{HOMO}	-6.342	-6.276	-6.294	-6.264
		E_{LUMO}	-2.093	-2.090	-1.530	-1.513

		ΔE	4.249	4.186	4.764	4.751
Benzene	CAM-B3LYP	E_{HOMO}	-6.424	-6.360	-6.372	-6.344
		E_{LUMO}	-1.858	-1.854	-1.292	-1.280
		ΔE	4.566	4.506	5.080	5.064

2.2. Femtosecond- and picosecond-to-microsecond transient absorption spectroscopy and singlet oxygen quantum yields.

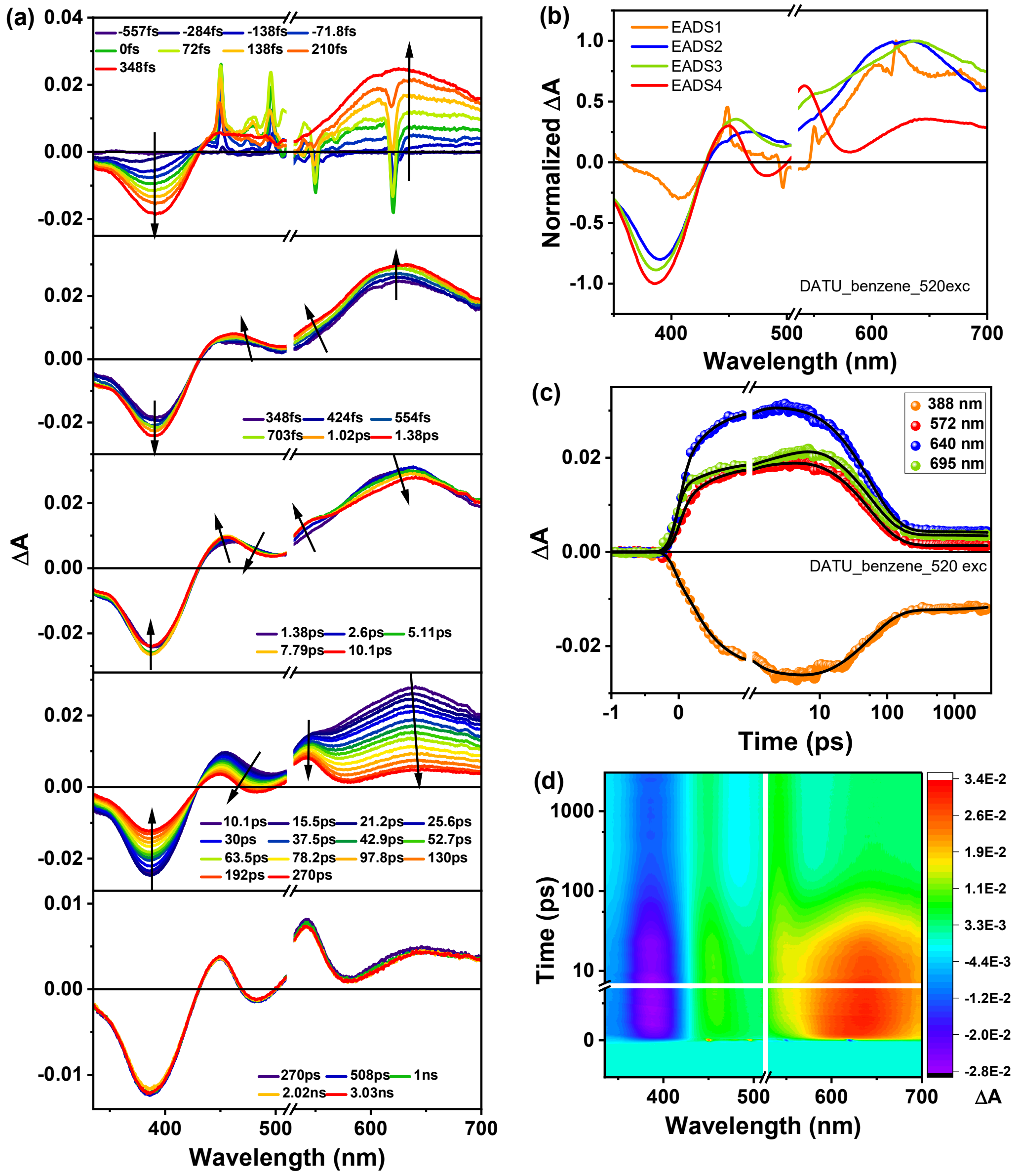

Figure S9. (a) Spectral evolution of fs-TAS of DATU in benzene at 520 nm excitation, (b) extracted EADS, (c) kinetic decay traces with fits at different wavelengths, and (d) contour plots

of fs-TAS. The breaks on the x-axis in panels (a, b, and d) cover the scattering from the pump pulse, and those on the x-axis in panel (c) and the y-axis in panel (d) represent the change in the scale from linear to logarithmic. The sharp signals in the top panel of panel (a) correspond to the coherent stimulated Raman scattering signals from the solvent, which were used to define time-zero at its maximum amplitude.

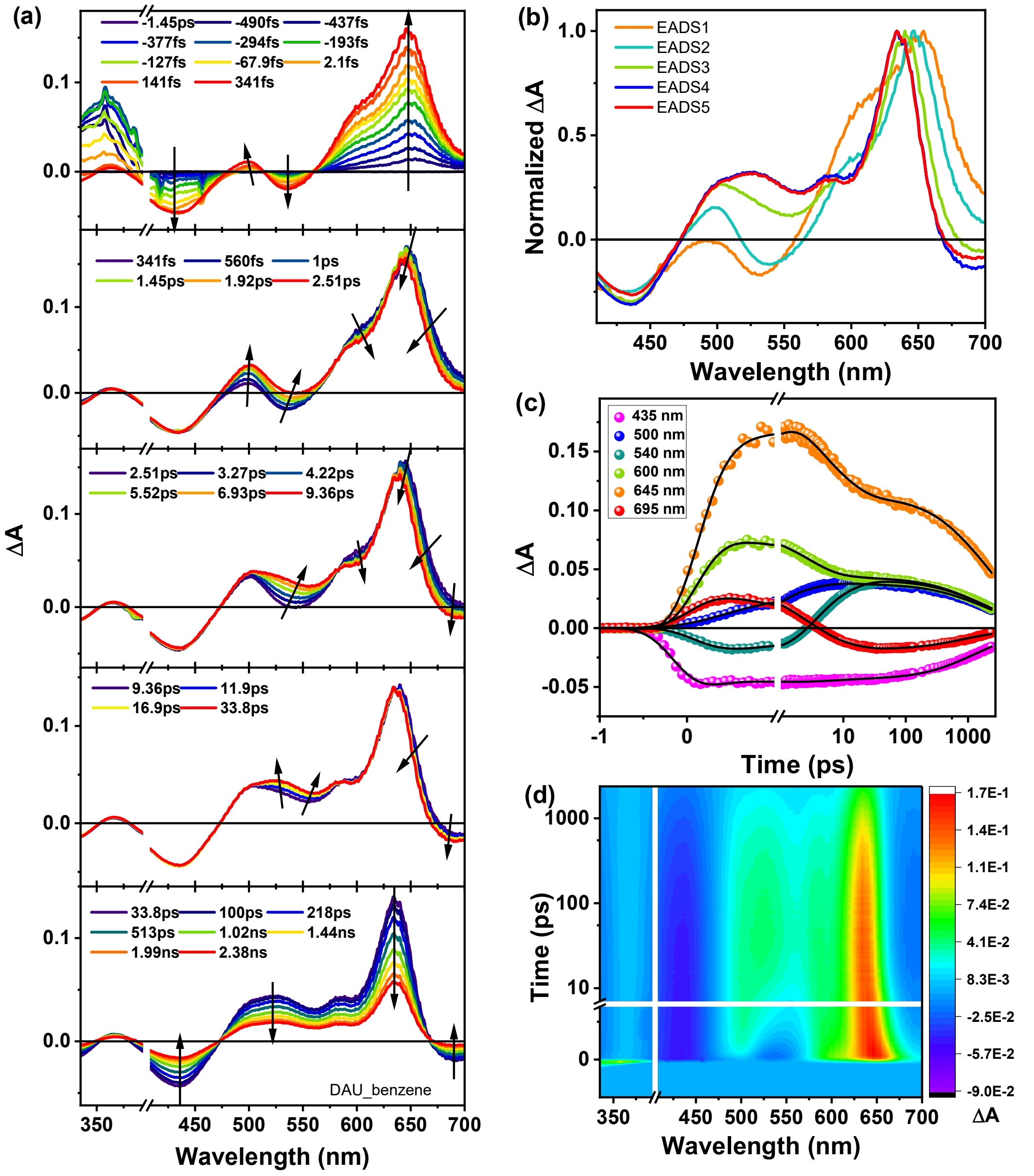

Figure S10. (a) Spectral evolution of fs-TAS of DAU in benzene at 400 nm excitation, (b) extracted EADS, (c) kinetic decay traces with fits at different wavelengths, and (d) contour plots of fs-TAS. The breaks on the x-axis of panels (a and d) cover the scattering from the pump pulse, and those on the x-axis of panel (c) and the y-axis in panel (d) represent the change in the scale from linear to logarithmic. The relatively small sharp signals in the top panel of panel (a)

correspond to the coherent stimulated Raman scattering signals from the solvent, which were used to define time-zero at its maximum amplitude.

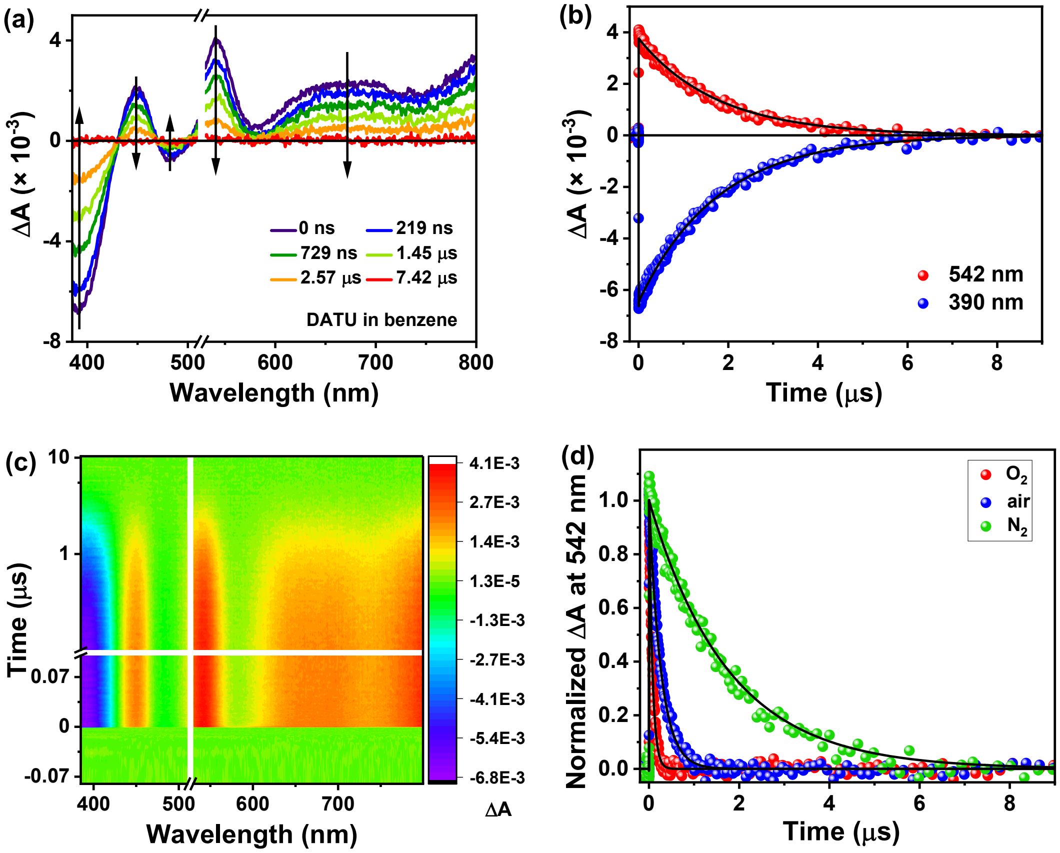

Figure S11. (a) ps-to-µs-TAS of DATU, (b) the respective kinetic traces, and (c) the contour plots for TAS data in N_2-saturated benzene following excitation at 520 nm. (d) The representative kinetic traces of TAS recorded under O_2, air, and N_2-saturated conditions. Breaks on the x-axis of panels (a) and (c) cover the scattering from the pump pulse, while that on the y-axis of panel (c) represents the change in the scale from linear to logarithmic.

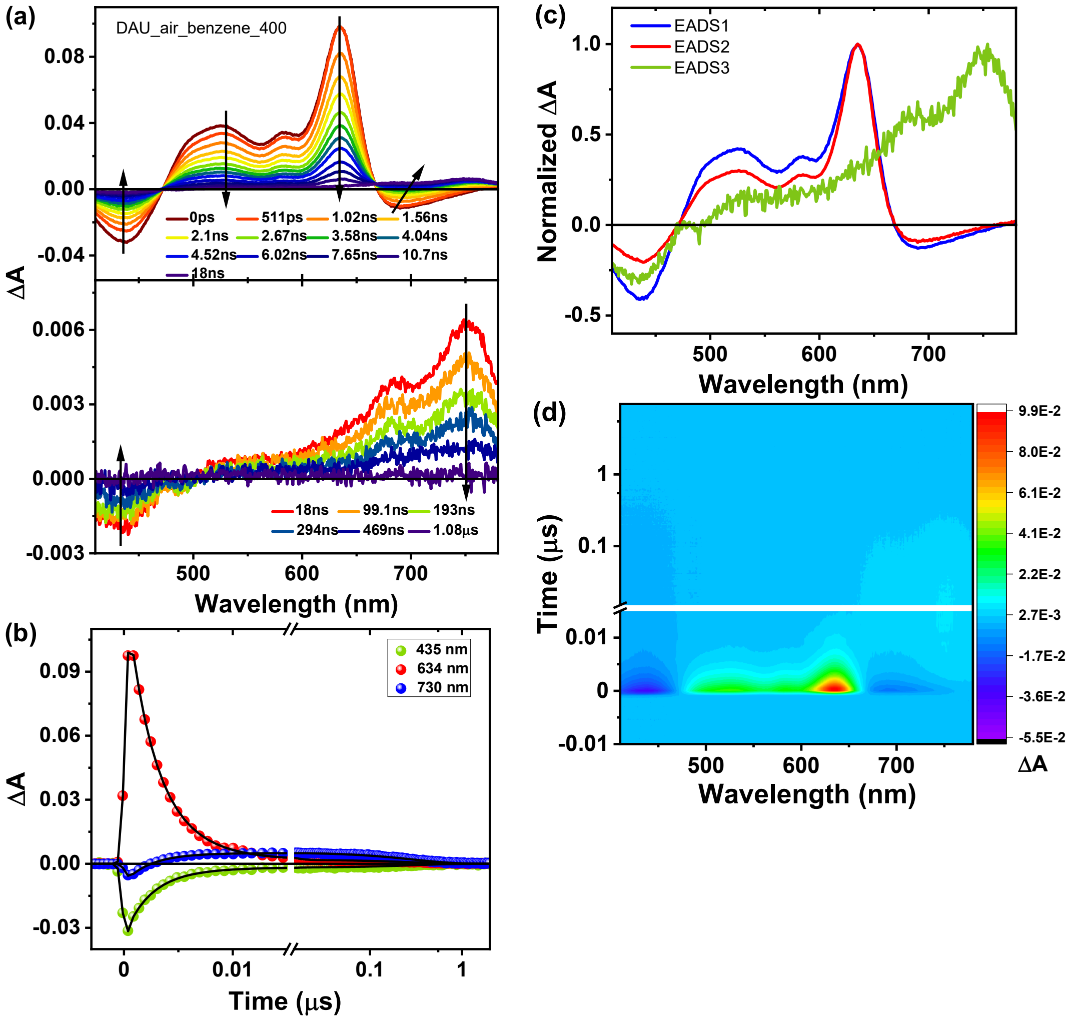

Figure S12. (a) ps-to-μs-TAS of DAU, (b) the respective kinetic traces, (c) the EADS in air-equilibrated benzene following excitation at 400 nm, and (d) contour plots of TAS data. Breaks on the x-axis of panel (b) and the y-axis in panel (d) represent the change in the scale from linear to logarithmic.

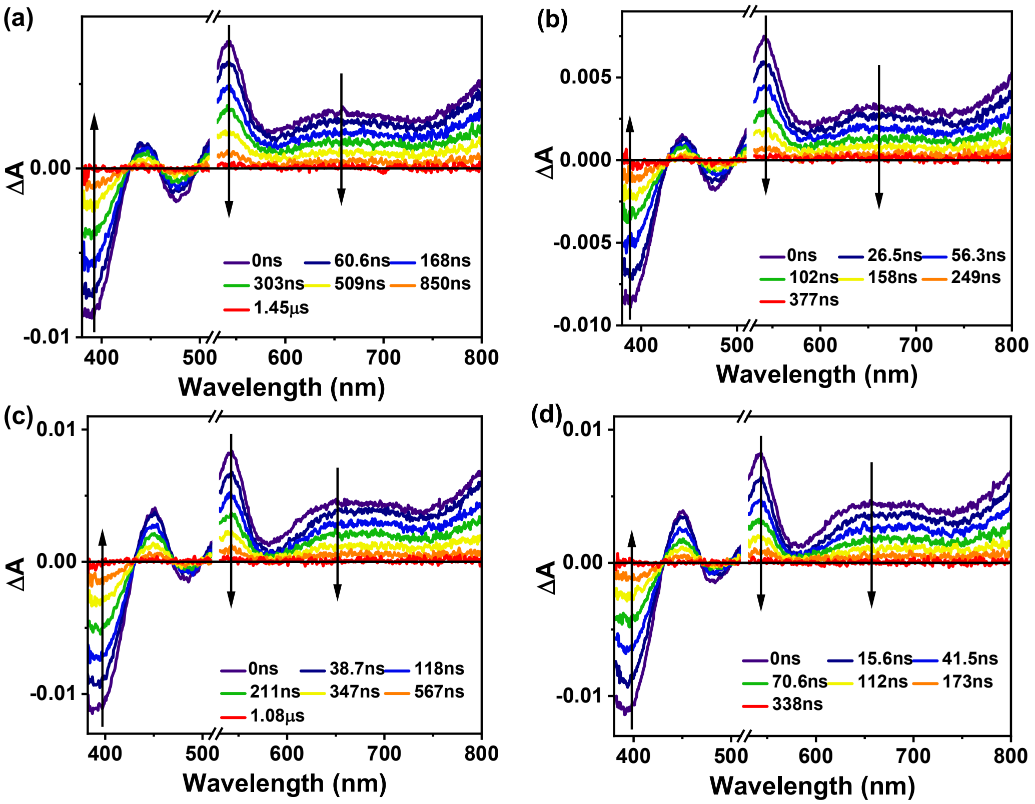

Figure S13. ps-to-µs-TAS of DATU in (a) air-equilibrated and (b) O_2-saturated 1,4-dioxane, and (c) air-equilibrated and (d) O_2-saturated benzene following excitation at 520 nm. Breaks on the x-axis of all the panels cover the scattering from the pump pulse.

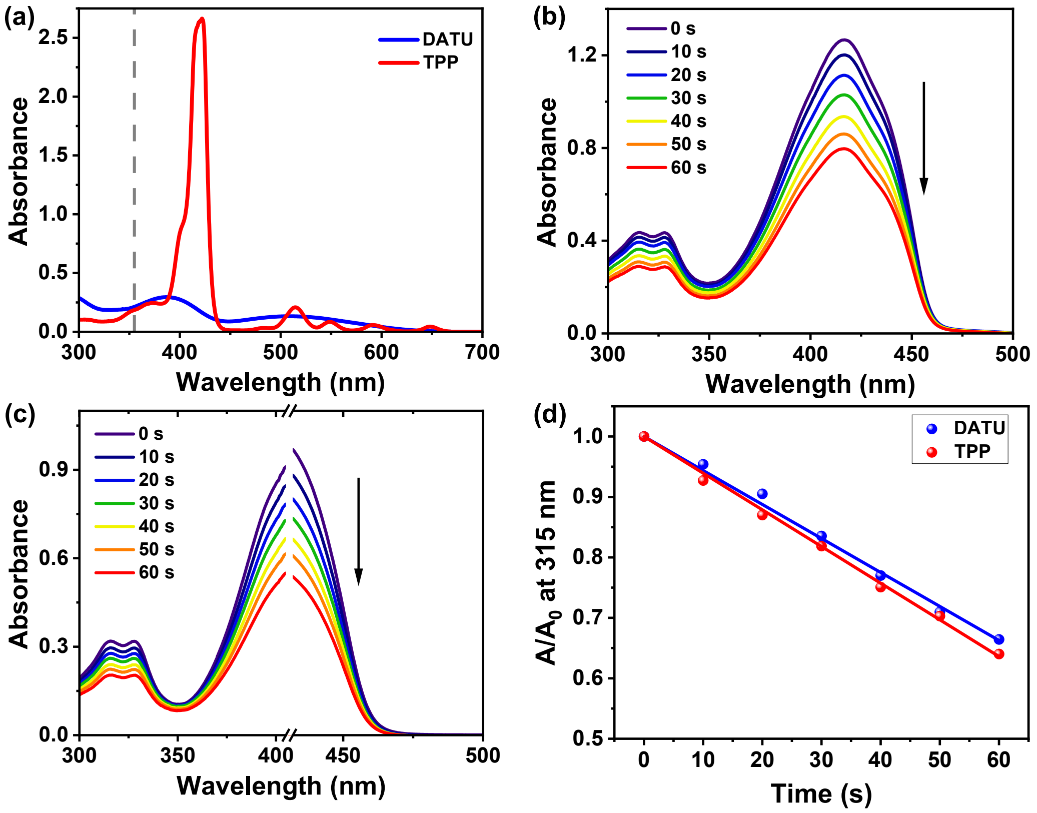

Figure S14. (a) Absorption spectra of DATU and TPP (Φ_Δ standard) in benzene obtained during the measurements, with the vertical dashed line marking the 355 nm excitation wavelength. (b, c) Degradation of DPBF at various 355 nm irradiation times in the presence of (b) DATU and (c) TPP in benzene, with baselines corrected using samples containing DATU or TPP prior to DPBF addition (see Experimental). The x-axis break in panel (c) spans the detector-saturated region caused by the combined strong absorption of TPP and DPBF. (d) Plot of A/A_0 at 315 nm versus irradiation time for DPBF in the presence of DATU and TPP, used to determine Φ_Δ.

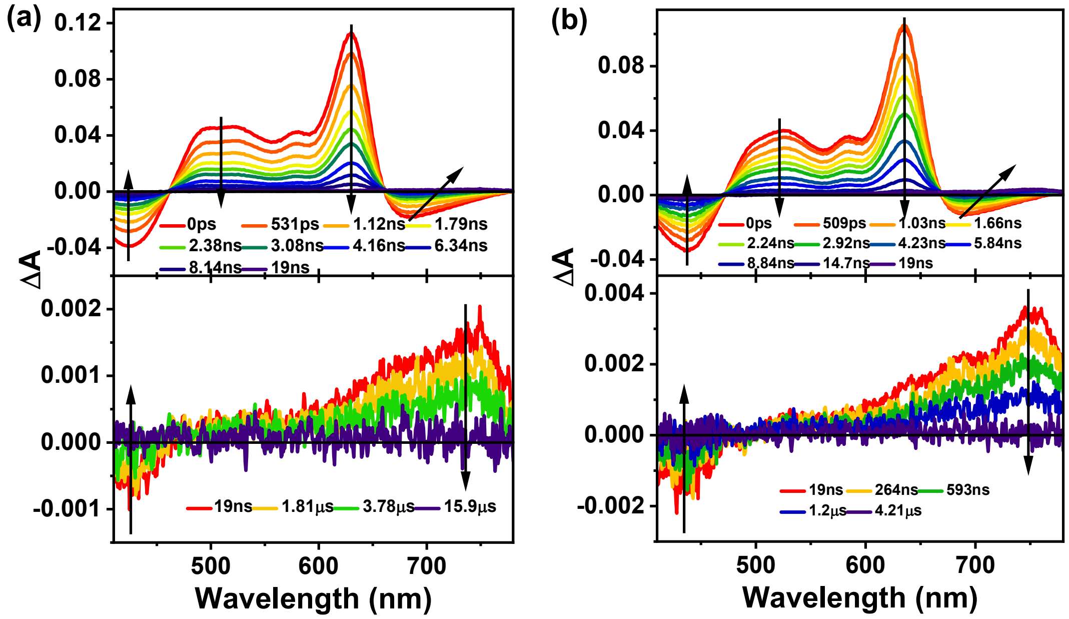

Figure S15. ps-to-μs-TAS of DAU in N_2-saturated (a) 1,4-dioxane and (b) benzene following excitation at 400 nm.

Verification of the two-photon absorption process at 800 nm.

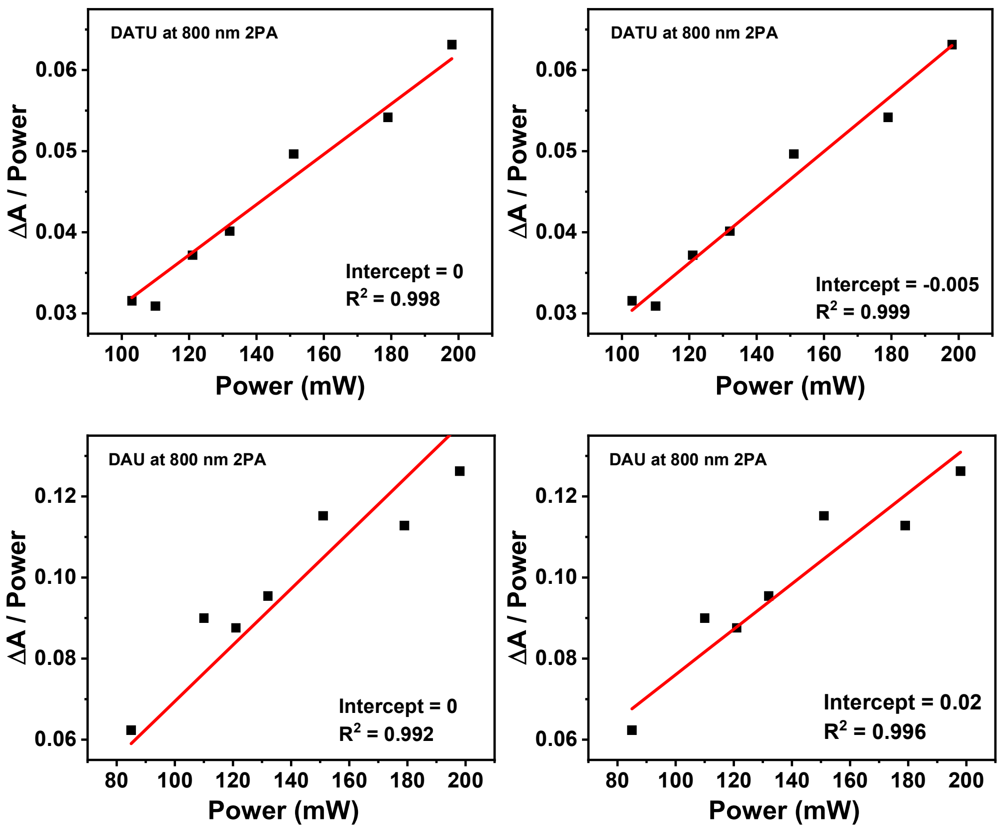

Figure S16. Plots of ΔA/P against P for DATU (top) and DAU (bottom) upon two-photon absorption at 800 nm with intercept fixed to zero (left planes) or allowed to take any value (right panels).

The minor deviation from the theoretical slope of 2.0 is mainly due to experimental uncertainty. Contributions from one-photon absorption at 800 nm have been excluded because DAU does not exhibit one-photon absorption beyond approximately 550 nm (Figure 1b), and DATU does not exhibit one-photon absorption beyond ca. 650 nm in dioxane (Figure 1a).

An effective method for examining whether the data in Figure 9a and 9b includes a linear combination of one- and two-photon absorption processes is to plot ΔA/P against P, as illustrated in Figure S16. If the data represents a combination of both one- and two-photon processes, the intercept of a plot of ΔA/P against P should be positive and not equal to zero. In contrast, if the data solely reflects a two-photon process, the intercept should be zero. Figure S16 shows the results of this analysis for DATU (top) and DAU (bottom). In the left panels of Figure S16, the linear regression is constrained to pass through a zero intercept (i.e., the intercept is fixed at 0), while in the right panels, the intercept is allowed to take any value. The goodness of fit in these plots indicates that all results have R^2 values greater than 0.99, demonstrating that any deviations from quadratic behavior are primarily due to experimental uncertainties in the collected data.

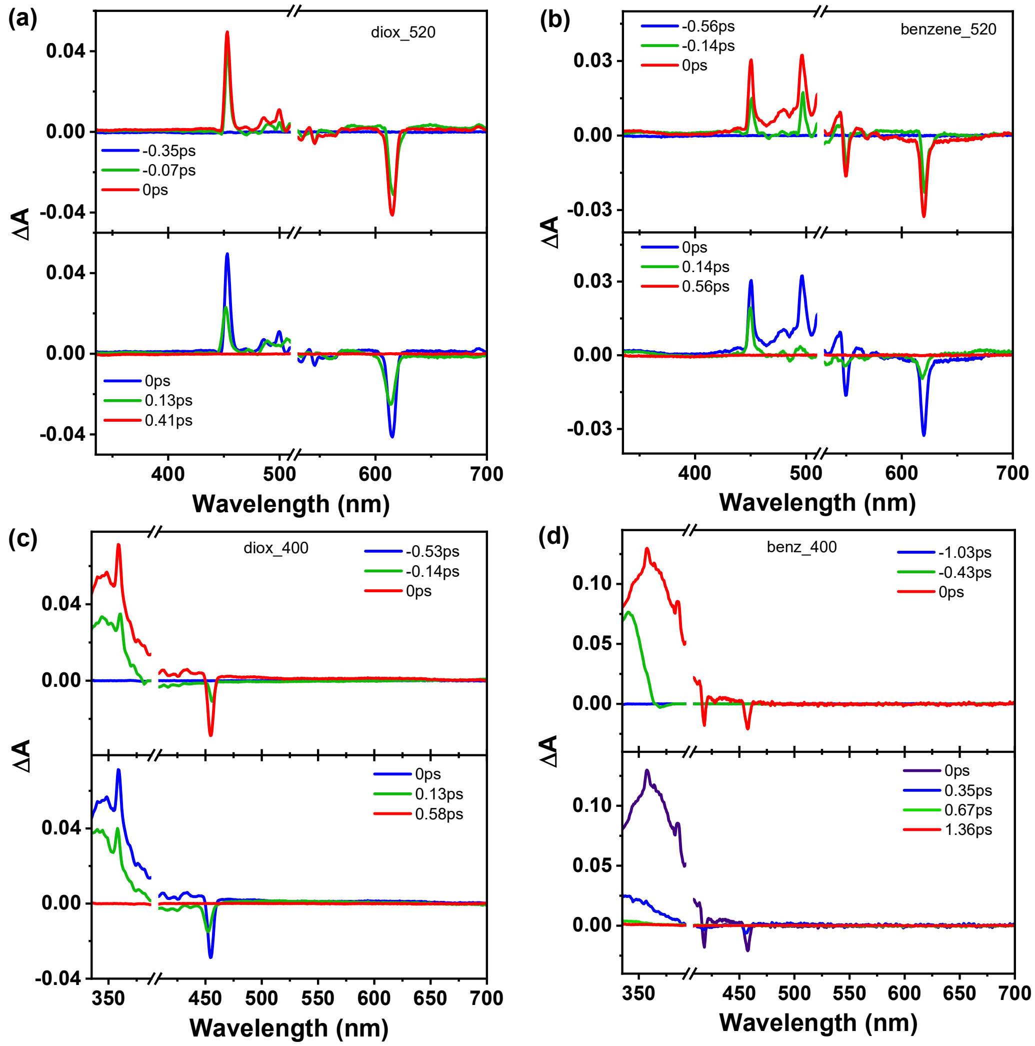

Figure S17. Coherent solvent and stimulated Raman signals observed in the fs-TAS of pristine (a) 1,4-dioxane and (b) benzene upon 520 nm excitation, and of pristine (c) 1,4-dioxane and (d) benzene upon 400 nm excitation, recorded within the temporal cross-correlation of the pump and probe pulses. Breaks on the x-axis of all the panels cover the scattering from the pump pulse.

Role of sugar protection/deprotection on the photophysics of DATU, as predicted by DFT and TDDFT calculations.

Does the deprotection of the ribose sugar in DATU upon enzymatic hydrolysis significantly change its photophysical properties? To answer this question, we performed DFT and TDDFT calculations to predict whether deprotection of the benzoyl group would result in significant changes in DATU's photophysical properties. As shown in Figures S18 and S19, the hydrolysis of the benzoyl protecting groups is expected to have a minor effect on the photophysics of DATU, including its triplet state lifetime and 2PACS, because the protected or free sugar is not part of the primary chromophore. In support of this argument, the one- and two-photon in vitro cell experiments

presented in Figure 13 demonstrate the excellent PDT activity of DATU in the 4T1 murine mammary carcinoma cell line.

DFT and TD-DFT simulations were performed for both the protected and deprotected DATU (see Figures S18 and S19 below). The ground-state geometries of *syn*-rotamers of both DATU(OBz) and DATU(OH) were optimized at the B3LYP-D3BJ/def2-SVP/CPCM level of theory in 1,4-dioxane. For ground state optimizations, we had to replace the larger def2-TZVPD basis set with a smaller def2-SVP basis to enable optimization of these large structures within our computational resources. Vertical excitation energies and simulated absorption spectra were subsequently computed at the TD-M062X/def2-TZVPD/CPCM level in 1,4-dioxane; the same level of theory used in the main manuscript. The resulting spectra clearly evidence that the two lowest-energy absorption bands are nearly identical for both molecules, exhibiting only minor spectral shifts at longer wavelengths than ca. 300 nm (Figure S18).

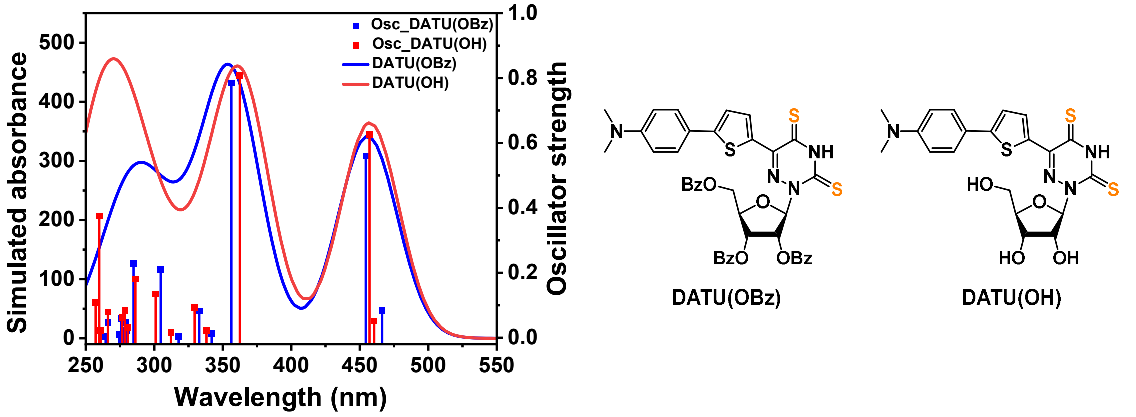

Figure S18. Simulated absorption spectra of the *syn*-rotamer of ribose sugar-protected and deprotected-DATU: DATU(OBz) and DATU(OH) (left). Structures of DATU(OBz) and DATU(OH) (right).

Similarly, the electron density distributions of Kohn-Sham frontier orbitals for both DATU(OBz) and DATU(OH) shown in Figure S19 are very similar in both systems. Collectively, the experimental and computational results demonstrate that the protected/deprotected ribose only has minor effects on the photophysics of DATU. However, transient absorption experiments for the free-sugar DATU in solution are required to validate these computational predictions.

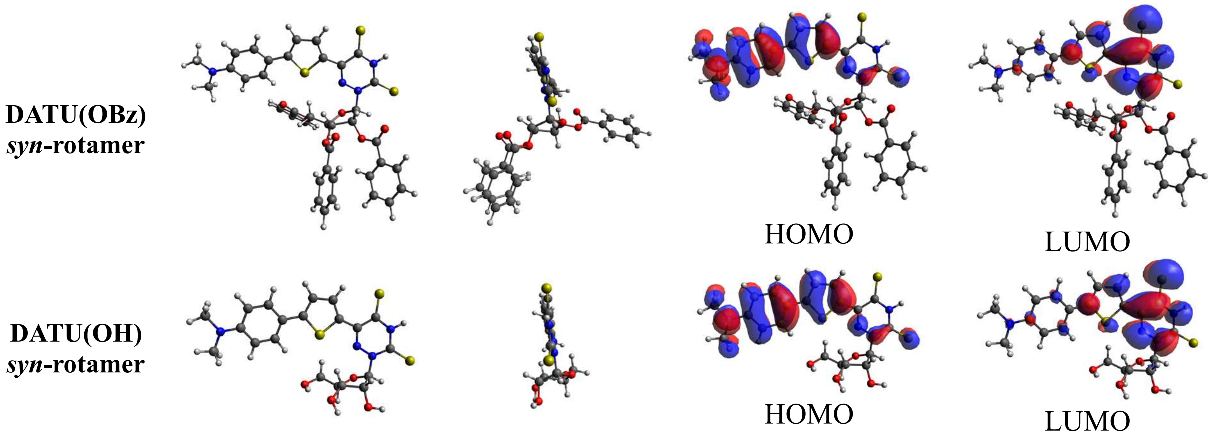

Figure S19. Electron density distributions of Kohn-Sham frontier orbitals for both (top) DATU(OBz) and (bottom) DATU(OH).

3. Synthesis

The synthetic routes for DAU and DATU are depicted in Scheme S1. The synthesis of DAU was carried out following a modified literature procedure.[23] The intermediates **C1**, **C2**, and **C3** were prepared as previously reported by our group.[24] Accordingly, only the synthetic procedures for DAU and DATU are detailed herein.

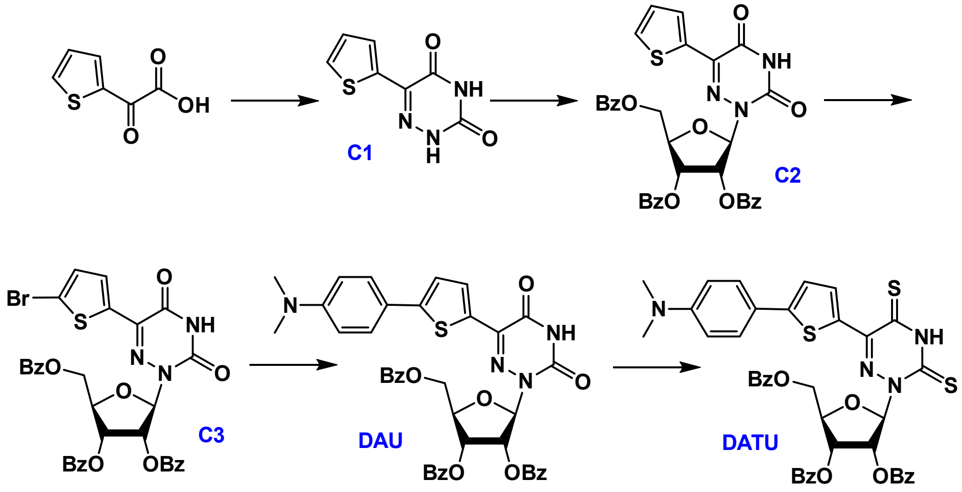

Scheme S1. Synthetic outlines for DATU and DAU.

Synthesis of DAU. Sodium carbonate (140 mg, 1.32 mmol) was dissolved in water (3 mL) under a nitrogen atmosphere. While maintaining inert conditions, 4-(dimethylamino)phenylboronic acid (97 mg, 0.59 mmol), compound **C3** (300 mg, 0.42 mmol), and acetonitrile (5 mL) were added to the solution, and nitrogen purging was continued for an additional 15 minutes. Palladium acetate (6.7 mg, 0.03 mmol) and tris(3-sulfophenyl)phosphine trisodium salt (85 mg, 0.15 mmol) were

then added, and the reaction mixture was stirred at 65°C for 20 hours. Upon completion, the reaction was cooled to room temperature and neutralized with saturated NH_4Cl solution. Acetonitrile was removed under reduced pressure using a rotary evaporator, and the aqueous phase was extracted with ethyl acetate (4 × 15 mL). The combined organic layers were dried and concentrated to afford the crude product. The crude product was purified by silica gel column chromatography using a mixture of ethyl acetate and hexane as the eluent to obtain DAU as a red solid. Yield – 170 mg (54%). ^{1}H-NMR (500 MHz, DMSO-d_6): δ 12.51 (s, 1H), 8.01 (d, 1H), 7.97 (d, 2H), 7.89 (d, 2H), 7.87 (d, 2H), 7.69 (t, 1H), 7.64 (t, 1H), 7.55 - 7.51 (m, 5H), 7.43 (t, 2H), 7.35 (d, 1H), 7.29 (t, 2H), 6.71 (d, 2H), 6.53 (d, 1H), 6.23 - 6.21 (m, 1H), 6.07 (dd, 1H), 4.87-4.84 (m, 1H), 4.73 – 4.70 (m, 1H), 4.63 – 4.59 (m, 1H), 2.96 (s, 6H). ^{13}C-NMR (125 MHz, DMSO-d_6): δ 165.38, 164.63, 155.44, 150.37, 148.23, 147.80, 138.34, 133.98, 133.82, 133.42, 131.12, 130.78, 129.48, 129.29, 129.16, 129.04, 128.87, 128.70, 128.59, 128.55, 128.51, 126.53, 121.54, 120.76, 112.27, 87.97, 78.36, 74.48, 71.07, 63.68. ESI-MS (positive-ion mode): m/z for $[M+Na]^+$ calcd. = 781.19, found = 781.40, and ESI-MS (negative-ion mode): m/z for $[M-H]^-$ calcd. =757.20, found = 757.13.

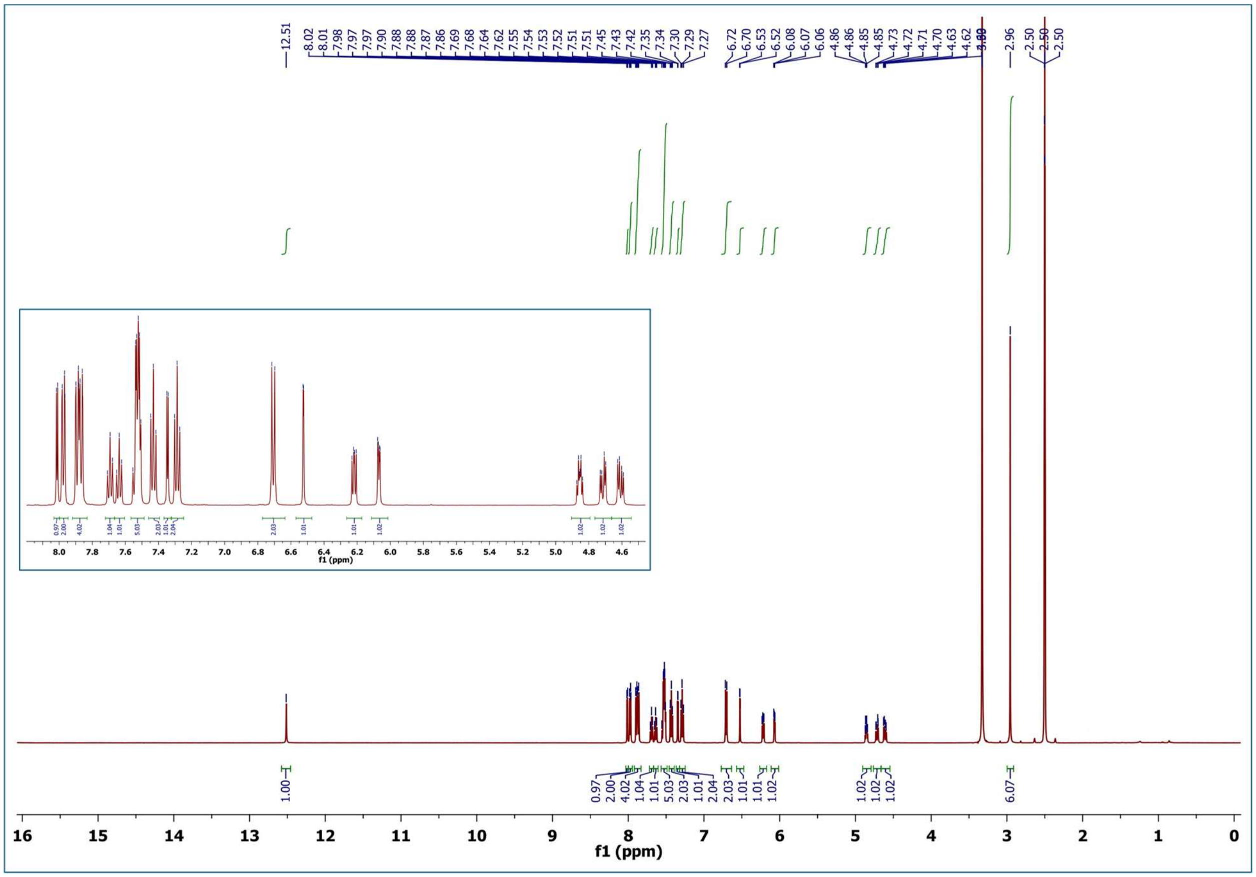

Figure S20. ^{1}H-NMR spectrum of DAU in DMSO-d_6. The inset shows a zoomed-in view of the spectral region approximately from δ 4.5 to 8.1 ppm.

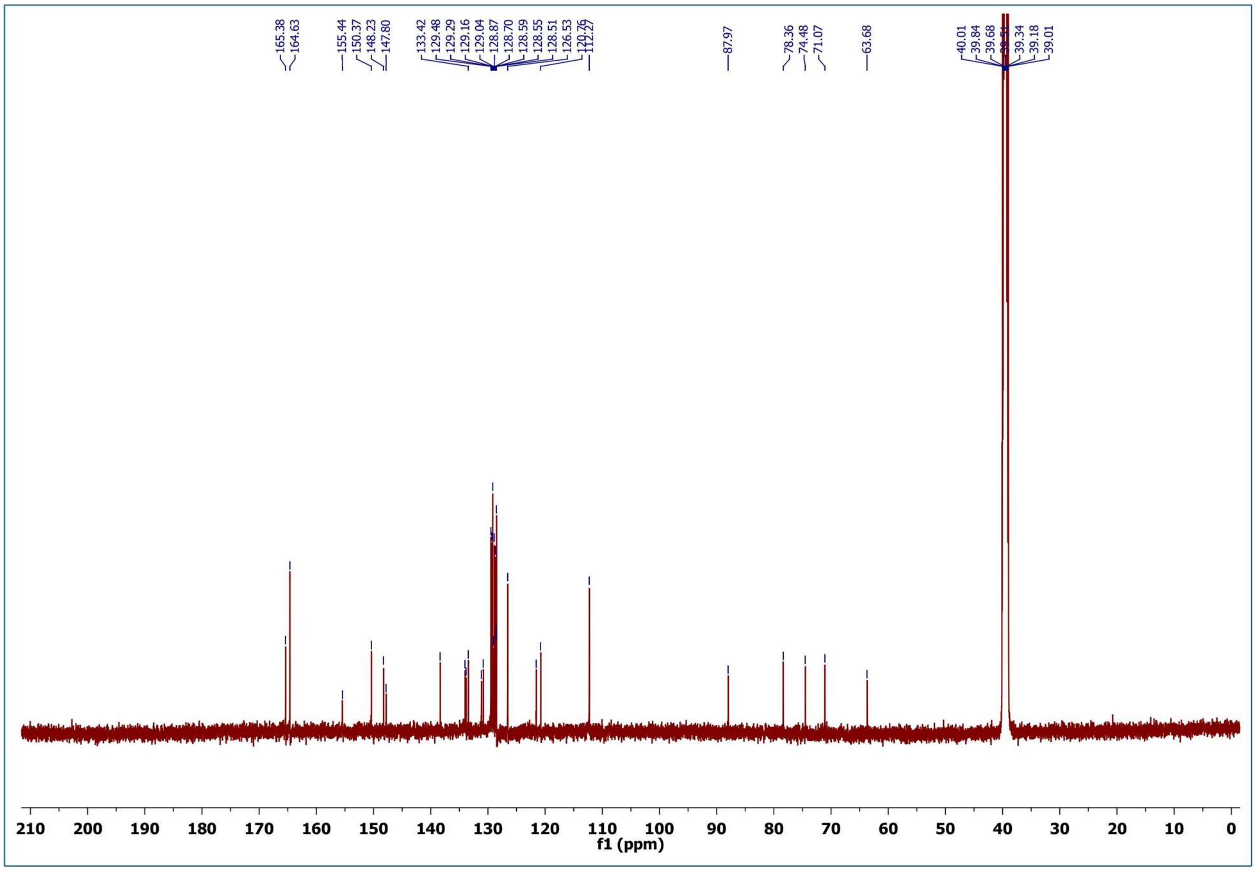

Figure S21. ^{13}C-NMR spectrum of DAU in DMSO-d_6.

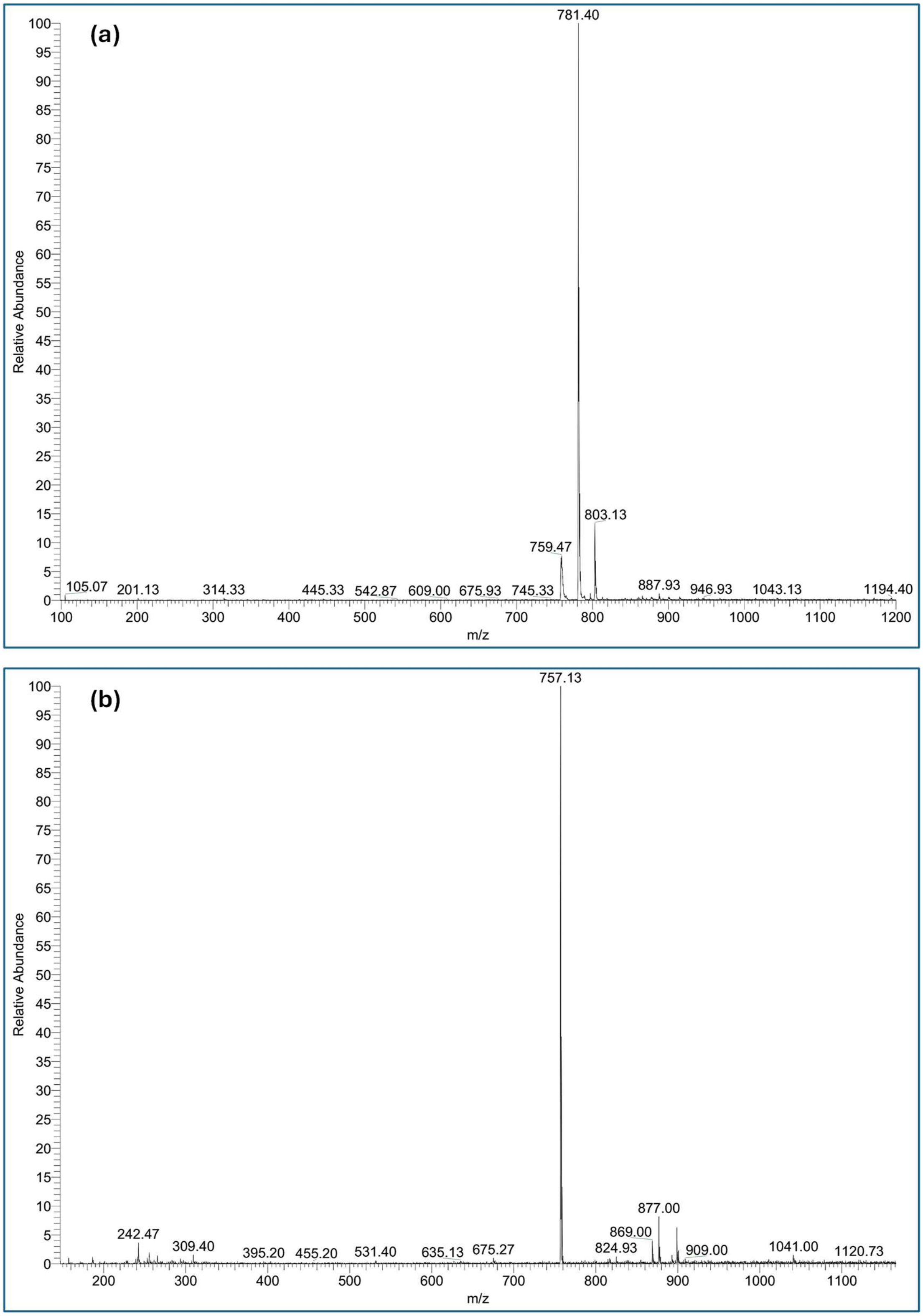

Figure S22. ESI-MS spectra of DAU in (a) positive-ion and (b) negative-ion modes.

Synthesis of DATU. DAU (76 mg, 0.10 mmol) was dissolved in toluene (7 mL) in an oven-dried Schlenk flask under a nitrogen atmosphere. The solution was stirred for 10 minutes while being continuously purged with nitrogen. Lawesson's reagent (162 mg, 0.40 mmol) was then added under nitrogen, and the resulting mixture was refluxed under dark conditions for approximately 24 hours. Upon completion, as monitored by TLC, the reaction mixture was concentrated under reduced pressure using a rotary evaporator to remove toluene. The crude residue was treated with methanol (10 mL), stirred at room temperature for 5–6 hours, and then concentrated under reduced pressure. The resulting solid was purified by silica gel column chromatography using a gradient of ethyl acetate and hexane as the eluent, affording pure DATU as a dark blackish-red solid with a product yield of 35 mg (44%). The entire process was carried out under light-protected conditions. ^{1}H-NMR (500 MHz, DMSO-d_6): δ 14.77 (s, br, 1H), 8.34 (d, 1H), 7.99 (d, 2H), 7.85 – 7.83 (m, 4H), 7.71 (t, 1H), 7.63 (t, 1H), 7.60 - 7.50 (m, 5H), 7.42 (t, 2H), 7.35 – 7.33 (m, 2H), 7.26 (t, 2H), 6.70 (d, 2H), 6.27 – 6.25 (m, 1H), 6.16 (dd, 1H), 4.93 - 4.90 (m, 1H), 4.76 - 4.72 (m, 1H), 4.65 – 4.61 (m, 1H), 2.96 (s, 6H). ^{13}C-NMR (125 MHz, DMSO-d_6): δ 176.25, 167.79, 165.35, 164.59, 164.54, 150.48, 150.01, 144.65, 134.06, 133.98, 133.85, 133.38, 131.43, 129.51, 129.28, 129.10, 128.97, 128.95, 128.70, 128.52, 128.49, 126.69, 121.13, 120.61, 112.22, 92.26, 78.70, 74.83, 70.89, 63.30. ESI-MS (positive-ion mode): m/z for $[M+Na]^+$ calcd. = 813.15, found = 813.27, and ESI-MS (negative-ion mode): m/z for $[M-H]^-$ calcd. =789.15, found = 788.93.

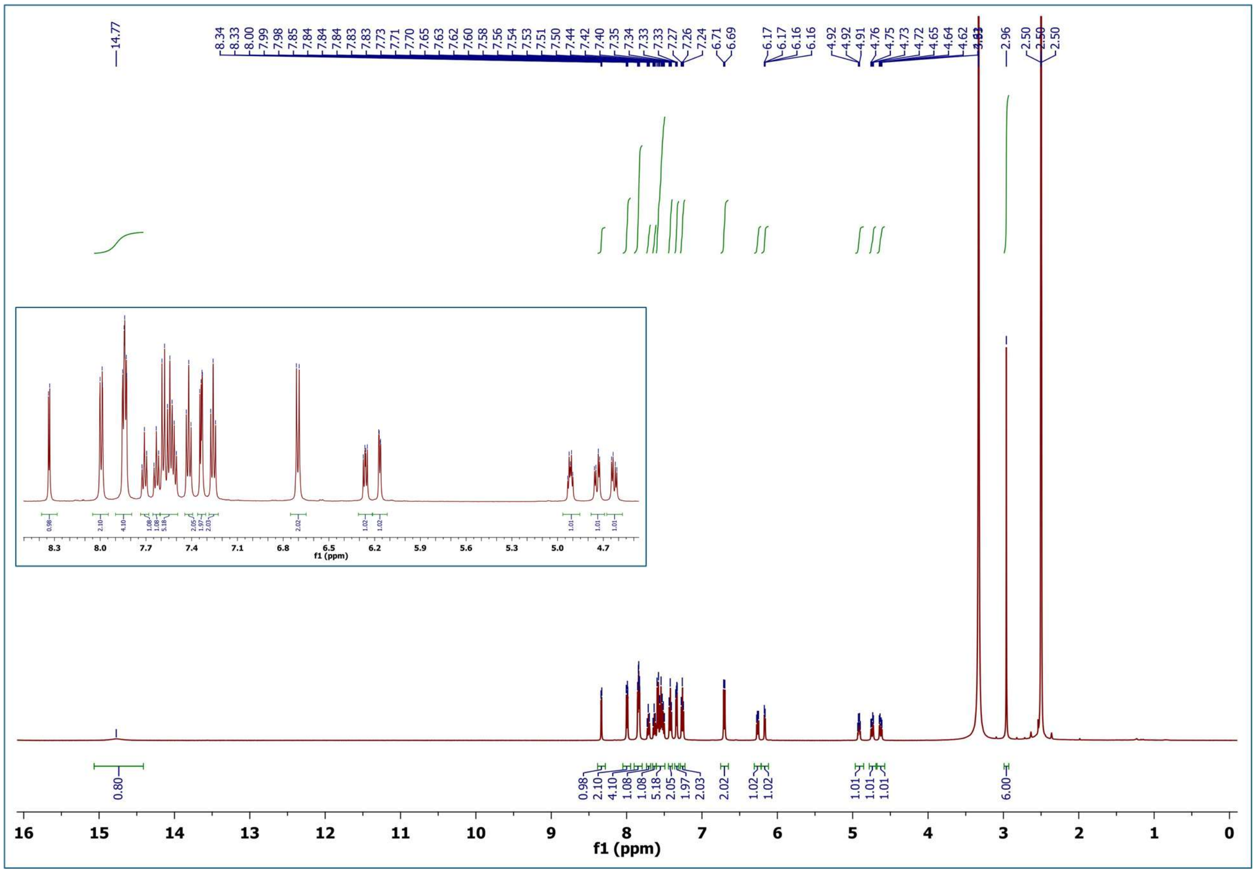

Figure S23. ^{1}H-NMR spectrum of DATU in DMSO-d_6. The inset shows a zoomed-in view of the spectral region approximately from δ 4.5 to 8.5 ppm.

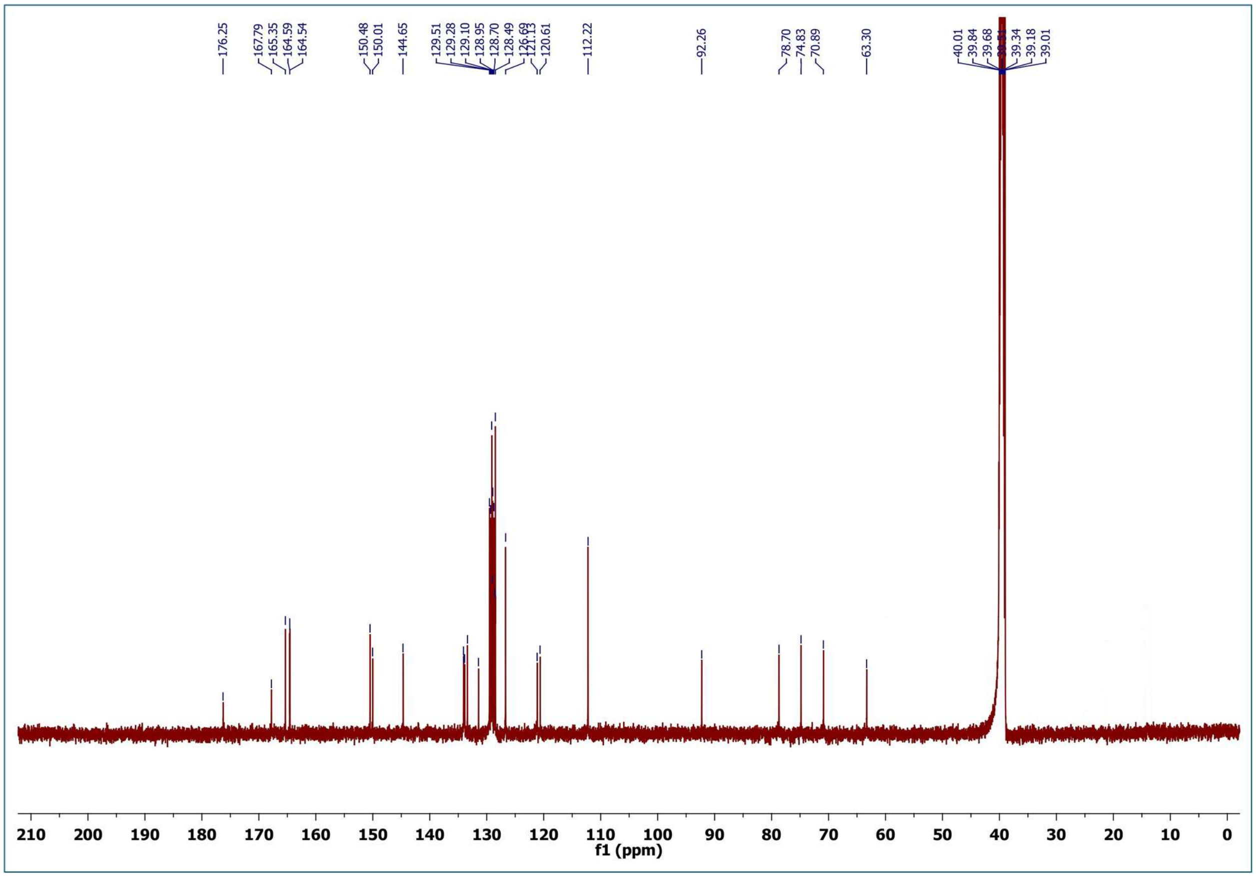

Figure S24. ^{13}C-NMR spectrum of DATU in DMSO-d_6.

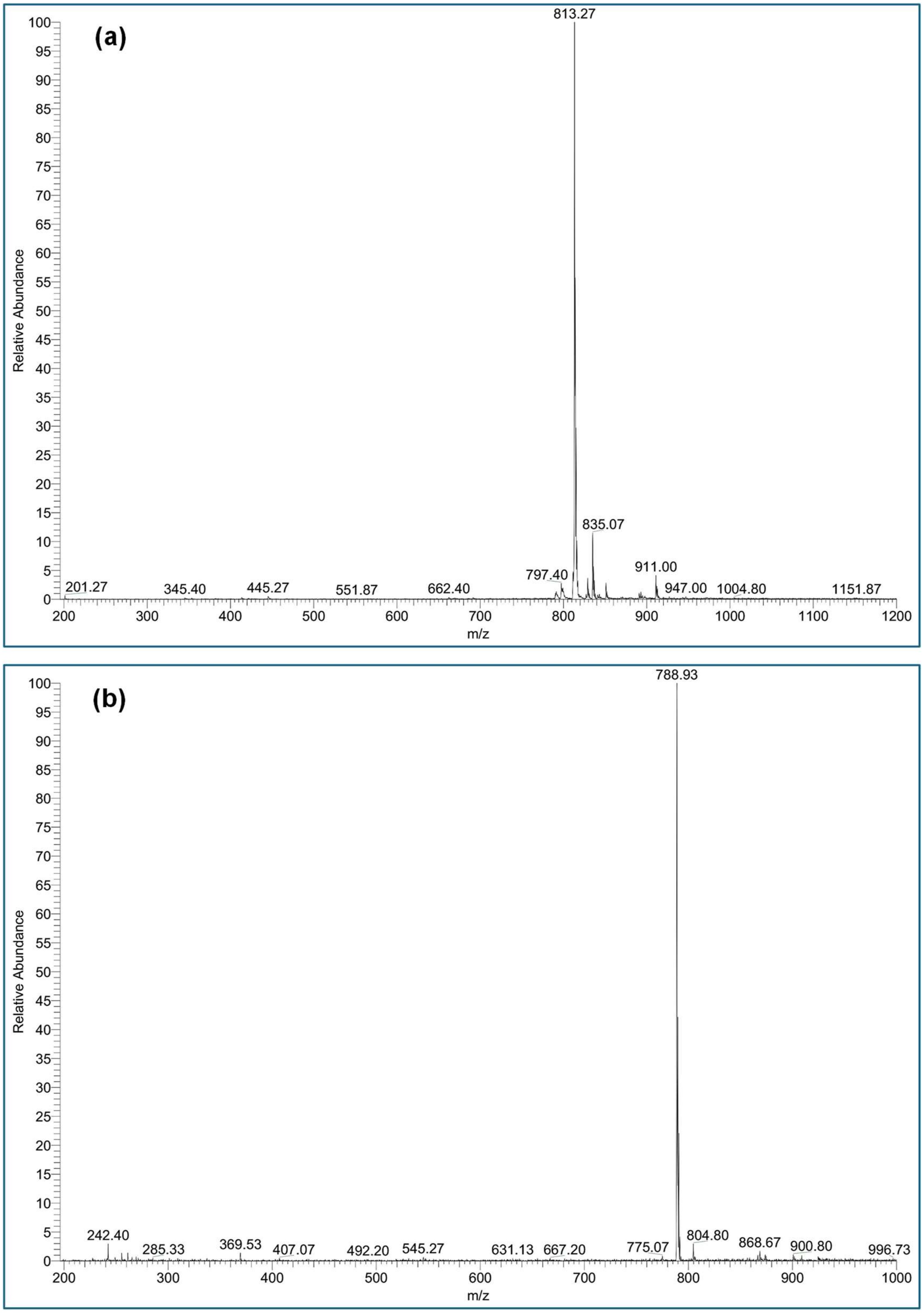

Figure S25. ESI-MS spectra of DATU in (a) positive-ion and (b) negative-ion modes.

Synthesis of tetraphenylporphyrin (TPP). TPP was synthesized following a literature procedure.[25,26] Benzaldehyde (1 mL, 9.8 mmol) and pyrrole (0.68 mL, 9.8 mmol) were dissolved in glacial acetic acid (25 mL) and refluxed for 2 h. After cooling to room temperature, the reaction mixture was filtered through a fritted glass funnel to afford a purple solid, which was washed thoroughly with water followed by methanol. The crude product was purified by silica gel column chromatography using a dichloromethane/hexane eluent to give the desired compound. ^{1}H-NMR (500 MHz, $CDCl_3$): δ 8.85 (s, 8H), 8.22 (dd, 8H), 7.76 (m, 12H), −2.77 (s, 2H).

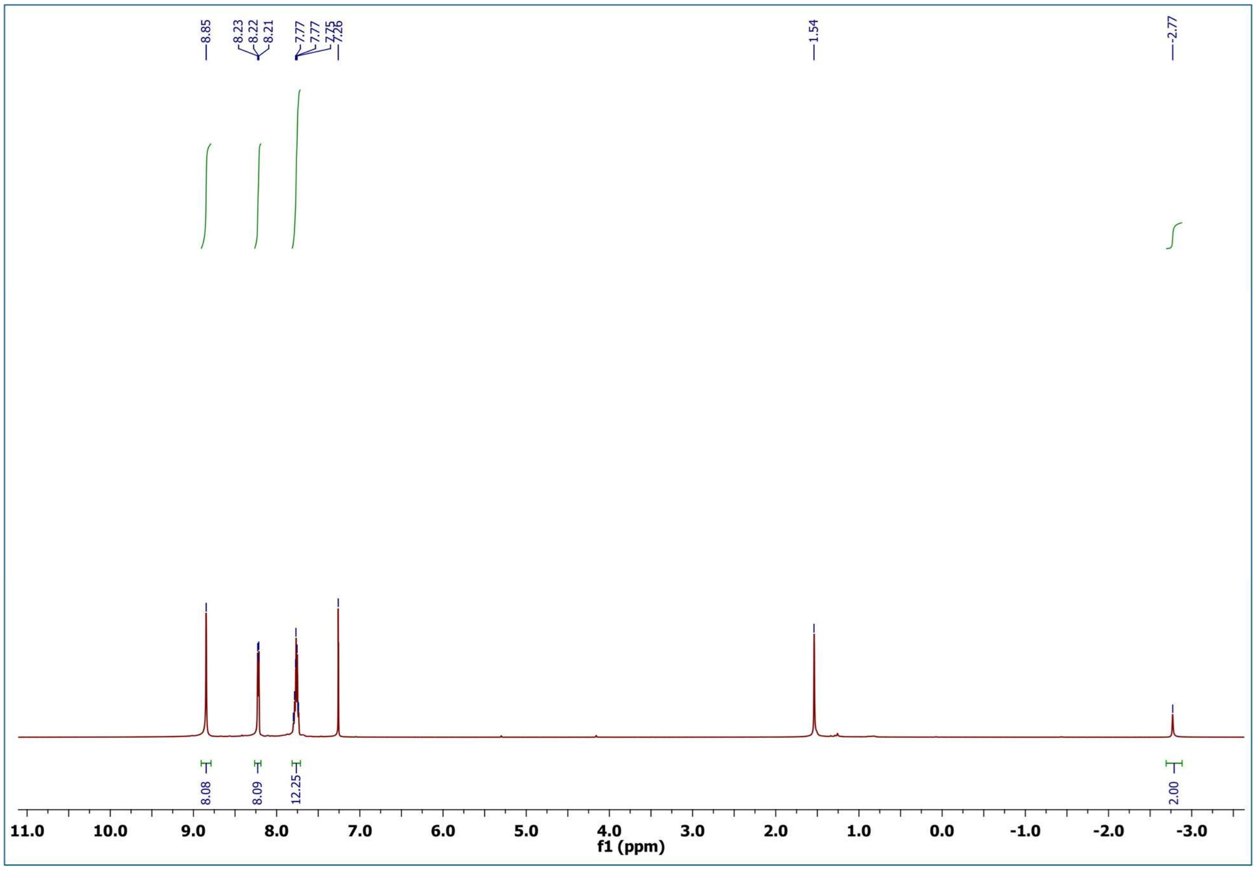

Figure S26. ^{1}H-NMR spectrum of TPP in $CDCl_3$.

4. Cartesian coordinates of the optimized geometries

4.1. In 1,4-dioxane

S_0 ($DATU_{syn}$)

C	-1.17141057149156	0.68479238016787	-0.05859520135205
C	-0.57842257989355	1.93092378956984	-0.10152492240532
C	0.82754971380933	1.89204649432595	-0.09508758271078
C	1.34367392087103	0.61318703079982	-0.04632653070654
S	0.04302296040813	-0.55464324488662	-0.00378788908993
H	-1.13890381204121	2.85232249248269	-0.15130576350076
H	1.44967744407078	2.76903137154153	-0.13047510448926
C	2.69799878962776	0.10750294401362	-0.02039082558345
C	3.91164962856033	0.94784325315722	-0.02594331339855
C	5.20355982048626	-1.14160941912048	0.05766210382648
C	-2.58518778724541	0.35310516480240	-0.04357564705023
C	-3.04484204784084	-0.95744609568023	-0.23746135987812
C	-3.55952241453119	1.33889048824983	0.17499092586975
C	-4.38750287130489	-1.27475077987892	-0.21377787958553
H	-2.33472160702578	-1.75352140244071	-0.42355194646434
C	-4.90649606504984	1.04126618688902	0.20141357652359
H	-3.25735777584654	2.36381030051424	0.34321725227802
C	-5.36615321203369	-0.28138821695446	0.01034250878338
H	-4.67972098120054	-2.30077812789912	-0.37545030307040
H	-5.60930418433431	1.84045461496267	0.38007560304451
N	5.07442684646767	0.21935861813526	0.01695741231191
H	5.93777170644052	0.74892850040304	0.01720923541886
N	4.00757795968629	-1.77354679869339	0.05070880206685
H	4.00369727603130	-2.78271679379802	0.07788572366122

N	2.80409731379768	-1.19760071153718	0.01552958037637
N	-6.69898016707759	-0.58672654439788	0.04274449929004
C	-7.68273110971932	0.45834764769283	0.24981706644930
H	-7.64065312799141	1.21602385789377	-0.53926911343262
H	-8.67532439510406	0.01739613106619	0.24464296101453
H	-7.53853129411629	0.96207293455439	1.21049675914684
C	-7.13936325342413	-1.95216910753685	-0.16801183981917
H	-6.71679688904156	-2.63047757301831	0.57930133706022
H	-8.22175647270009	-1.99258818906202	-0.08725586756959
H	-6.85607700473225	-2.32166097484019	-1.15901905636708
S	6.66359276472247	-1.93006567990083	0.10907521041755
S	4.00592347876661	2.59385545842301	-0.07611041106573

S_0 ($DATU_{anti}$)

C	-0.50275679430830	2.34647792990268	-0.13250487365419
C	-0.83673082789312	1.00486932977189	-0.07856506247765
C	0.27600692958858	0.15692450957508	-0.04478424276454
C	1.49115997500012	0.82046729885926	-0.07337241740166
S	1.22041444605079	2.54091814165341	-0.15285613854217
H	-1.85464155527093	0.64625127240985	-0.04539285235412
H	0.21097538565652	-0.91875728953225	0.00942266351632
C	2.77819972286200	0.15238114054709	-0.03117240720445
C	4.07655499929689	0.84880711069927	-0.05255722268143
C	5.13498385320956	-1.35839779999688	0.08454576675572
C	-1.39276943866329	3.49528758462618	-0.17339359369145
C	-0.93674777825888	4.80265587940239	0.04885525830579
C	-2.76168303100123	3.34091730021907	-0.43780159492157

C	-1.78494171051055	5.89113299079883	0.01218738584760
H	0.10865069675368	4.97663594091515	0.27133729432867
C	-3.62569469138445	4.41667180680469	-0.47578564739407
H	-3.16249887575461	2.35490052886375	-0.63100821528554
C	-3.16287191888963	5.73253259451352	-0.25296601675217
H	-1.37563472723292	6.87196861772018	0.19876470384196
H	-4.66803606210305	4.23570613566996	-0.68877646994845
N	5.15615124182801	0.00767867432044	0.01481639490652
H	6.07186740712685	0.43989584897685	0.00783988869431
N	3.87673934011137	-1.85431398943033	0.08109829863671
H	3.76403554025783	-2.85641999577990	0.12801520095697
N	2.73879359955396	-1.15507786862778	0.03087846013395
N	-4.01181763399451	6.80533333884848	-0.29235398136130
C	-5.42706467009548	6.60763943620530	-0.53630058737292
H	-5.88519066751638	5.97196830332821	0.22837112136980
H	-5.92754961744471	7.57156591752873	-0.52035956535861
H	-5.60815988262455	6.14637594815743	-1.51217225619540
C	-3.50849880980145	8.14302266853483	-0.04780142945342
H	-2.73530964122324	8.42000572724058	-0.77078266782142
H	-4.32626216043815	8.85207289118159	-0.13919550575105
H	-3.08251557942546	8.23956826496062	0.95635704285633
S	6.50121104980050	-2.29911591951126	0.16291880345781
S	4.31418188673824	2.47613973064300	-0.14818553522085

S_0 (DAU_{syn})

C	0.62218739973358	0.57705768261844	-0.32405132543037
C	-0.00842861591149	1.64628443889170	-0.92661929459013

C	-1.41446117252490	1.58341479576954	-0.87218302235470
C	-1.88448943235611	0.46149315226554	-0.22434593066816
S	-0.55720095874292	-0.52883415650595	0.31825429239004
H	0.52461029923738	2.46347942553887	-1.38873306202919
H	-2.07070628844821	2.33061191521042	-1.28572987899953
C	-3.24001328249347	0.03993533732978	0.03784910440327
C	-4.39693717389175	0.84543176484526	-0.44390105556406
C	-5.83984320869960	-0.87516641634355	0.58817164046845
C	2.04694697373153	0.31629274816761	-0.19911607104918
C	2.54824518498013	-0.73316199522058	0.58341477397535
C	2.99084641807427	1.11018354732148	-0.86774130478624
C	3.90120907188531	-0.98325204248180	0.69775946052405
H	1.86404924213844	-1.37205705803613	1.12785736870572
C	4.34711011535249	0.87689012633866	-0.76474080609258
H	2.65856991683820	1.92792358883051	-1.49276526040039
C	4.84861150513152	-0.18293672151921	0.02325762472472
H	4.22497068611225	-1.80469069521564	1.31807376359245
H	5.02442587536552	1.52006396951659	-1.30523175439429
O	-4.32959321505251	1.88778050509669	-1.06357370917386
O	-6.94506440344513	-1.31069897565030	0.84885226445034
N	-5.62167385264917	0.29344077114244	-0.11533784710149
H	-6.44648384209124	0.79731317359025	-0.41655787452469
N	-4.67151703292467	-1.48747421721755	0.95865739322726
H	-4.73901407800435	-2.34930493880418	1.47791511243233
N	-3.42335409523267	-1.06184794068334	0.70234892803456
N	6.19203848612941	-0.42354901735774	0.12689447548827
C	7.14052938560251	0.42098452377563	-0.57212462372962

H	7.07439879531861	1.46326325839150	-0.24347680356444
H	8.14713905330441	0.06570058610467	-0.37113306773978
H	6.98086295421109	0.39711984940347	-1.65461597761631
C	6.67592084086999	-1.51267464015696	0.95221481180976
H	6.29650683637474	-2.47978479629954	0.60756131526959
H	7.76083153419852	-1.53834647554313	0.90574498598381
H	6.38210007787835	-1.38835507311343	1.99951135432907

S_0 (DAU_{anti})

C	0.08855882917274	1.76248021464461	-1.08945472239069
C	0.37960412644025	0.55209135069782	-0.48812183035261
C	-0.75889846055168	-0.13937713299095	-0.04565735502255
C	-1.94157873617803	0.52728026748726	-0.29638861217442
S	-1.62137926225230	2.04378065319829	-1.08769452819667
H	1.38376940313907	0.16628393724811	-0.39234652977670
H	-0.73161147580029	-1.10584197654672	0.43500294555663
C	-3.26969157590936	0.05583414298044	0.03623030530000
C	-4.46240965340253	0.87411632083686	-0.30233622934006
C	-5.81808207772516	-0.92221620530729	0.69964596055162
C	1.01214701405635	2.73257773650501	-1.65917487224548
C	0.59410343919057	3.71318292647489	-2.56901391220910
C	2.37119775574995	2.72749535990843	-1.31503501878946
C	1.47018225009360	4.63671388025800	-3.10473527980246
H	-0.44224902875072	3.74784082639568	-2.88112547346650
C	3.26283701317696	3.64072564257323	-1.84315289230604
H	2.73917135574890	2.00169740802434	-0.60189150373385
C	2.83839658429412	4.62999146708260	-2.75717343003412

H	1.09005950058152	5.36302028635994	-3.80643958655852
H	4.29595777749563	3.59259790545759	-1.53480948906911
O	-4.42385732324017	1.95963205262924	-0.84827247953126
O	-6.89903738021522	-1.38684217838793	1.00671272860008
N	-5.65971587073096	0.29300845868591	0.06031490299175
H	-6.50650452180760	0.80697260275257	-0.14891722652949
N	-4.62160052601887	-1.54182424147445	0.94843277653784
H	-4.64812838695896	-2.43583210543984	1.41435712235165
N	-3.39390227095676	-1.08961193283467	0.63582148075846
N	3.71437528805307	5.54341946281612	-3.28179459331485
C	5.11793963516795	5.49658806244634	-2.92431330824452
H	5.57497671773796	4.54133902049025	-3.20231251052768
H	5.64437386800781	6.28893097987630	-3.44877531383736
H	5.26726609033529	5.64203432527170	-1.84952154840394
C	3.24470041888930	6.55266704982713	-4.20995837647767
H	2.47144882870419	7.18398776779882	-3.76116080458844
H	4.07774231103714	7.18919350153202	-4.49446353284098
H	2.82974834342623	6.10647216272225	-5.11985726288335

4.2. In benzene

S_0 (DATU$_{syn}$)

C	-1.17140862349389	0.68455935754789	-0.05850019333439
C	-0.57848841987675	1.93065725016156	-0.10317608911593
C	0.82748025468637	1.89183866804055	-0.09668171372968
C	1.34368321994809	0.61306627061419	-0.04622122640538
S	0.04305952201607	-0.55468399416211	-0.00079492725808
H	-1.13906541680614	2.85190997212242	-0.15473963015234

H	1.44953893408701	2.76880729813802	-0.13371743805593
C	2.69803939628300	0.10750116850971	-0.02020963063971
C	3.91163312409164	0.94787638113926	-0.02658207581049
C	5.20360496645461	-1.14133626305648	0.05859988272808
C	-2.58517758272597	0.35286175168777	-0.04338532339398
C	-3.04493158512933	-0.95753218638791	-0.23813827876846
C	-3.55936614175355	1.33857424122952	0.17618616581897
C	-4.38761814581491	-1.27473715104517	-0.21445981705415
H	-2.33487274711158	-1.75347960647226	-0.42503564256052
C	-4.90636013500384	1.04105428365860	0.20258683374367
H	-3.25700537239442	2.36329901462683	0.34528753347344
C	-5.36616085368256	-0.28142217154632	0.01047637895443
H	-4.67997925477253	-2.30062877463389	-0.37676022005235
H	-5.60908704623535	1.84014168875234	0.38202866783725
N	5.07444717556001	0.21955304232826	0.01675757406772
H	5.93776903035602	0.74917555294343	0.01646919296525
N	4.00777593989237	-1.77341174983160	0.05226182546685
H	4.00391867039104	-2.78257500255343	0.08025693216175
N	2.80424927953140	-1.19757561974742	0.01673595748395
N	-6.69897057769009	-0.58662468189059	0.04268931568637
C	-7.68259815878689	0.45823601084686	0.25174212684828
H	-7.64141418221568	1.21659869229390	-0.53673267489276
H	-8.67514155097525	0.01718473551097	0.24730901411492
H	-7.53732287866250	0.96108427729274	1.21270109306221
C	-7.13963952660668	-1.95173440456183	-0.16989254203896
H	-6.71863578893088	-2.63093808688347	0.57751409036234
H	-8.22218517606184	-1.99166956581771	-0.09103616282337

H	-6.85487191273052	-2.32041259647430	-1.16075120418845
S	6.66382475990829	-1.92963561359018	0.11056791494563
S	4.00573680425535	2.59388781120989	-0.07820570944617

S_0 ($DATU_{anti}$)

C	-0.50284757930507	2.34642231834942	-0.13249567799533
C	-0.83679710986031	1.00495777106447	-0.07644616778873
C	0.27595464809618	0.15697195003775	-0.04343272599567
C	1.49106569574849	0.82040368354795	-0.07479064634914
S	1.22032171805714	2.54075370868355	-0.15644745248352
H	-1.85474291659519	0.64660698339881	-0.04098474247115
H	0.21101977262187	-0.91863186054687	0.01251691959777
C	2.77816066725899	0.15243080268494	-0.03228265578772
C	4.07651000706860	0.84856490264669	-0.05861800563246
C	5.13487181679185	-1.35810315827802	0.08589707697132
C	-1.39279297819943	3.49527926704240	-0.17318565876686
C	-0.93720224003347	4.80229586858840	0.05189227535829
C	-2.76122151588431	3.34114757387855	-0.44022868647346
C	-1.78543203286002	5.89075836535294	0.01539837079531
H	0.10778580280251	4.97588769018499	0.27662112586132
C	-3.62526779685310	4.41688744890043	-0.47798318812103
H	-3.16143544373675	2.35535141184157	-0.63585748632413
C	-3.16291851779384	5.73241429163502	-0.25222046226220
H	-1.37648336383940	6.87133341688054	0.20412737885993
H	-4.66721147843650	4.23614281958019	-0.69311582431002
N	5.15610641631879	0.00764750894007	0.01093473114069
H	6.07186102198426	0.43974165931355	0.00082920855907

N	3.87661982915203	-1.85397547906164	0.08565205839834
H	3.76399556267420	-2.85591515850960	0.13658964424524
N	2.73876839600640	-1.15477900656482	0.03485301658646
N	-4.01188045591779	6.80517383223317	-0.29113871325420
C	-5.42640910822800	6.60802812828771	-0.53976538954844
H	-5.88688605498337	5.97098812283755	0.22233595214365
H	-5.92689534322668	7.57194570785216	-0.52356779702688
H	-5.60460796280276	6.14855010697169	-1.51702142784868
C	-3.50895502574275	8.14259858229654	-0.04422750853217
H	-2.73596267336211	8.42134118047254	-0.76679079299166
H	-4.32699642695932	8.85152954886355	-0.13393005013096
H	-3.08281590310164	8.23725671436104	0.96000841874231
S	6.50105452656720	-2.29875141278777	0.16644780952384
S	4.31421604657329	2.47543470902038	-0.16225292668903

S_0 (DAU_{syn})

C	0.62234212788213	0.58042199617235	-0.31952679662039
C	-0.00820786232534	1.65298936386786	-0.91611944293357
C	-1.41430254206023	1.58949514450639	-0.86244310094683
C	-1.88430443325573	0.46377278436038	-0.22120261206152
S	-0.55695566188846	-0.52958585694317	0.31580034192714
H	0.52497613200904	2.47331471960897	-1.37254106028396
H	-2.07059897223502	2.33909945228874	-1.27152425360822
C	-3.23982313715897	0.04030515674015	0.03822846340789
C	-4.39688354892129	0.84708417130745	-0.44096839213942
C	-5.83942829912024	-0.88067390596671	0.57949548477738
C	2.04703809152562	0.31877627144586	-0.19591441677869

C	2.54867099709450	-0.72563621198732	0.59312800609090
C	2.99033479246054	1.10636391161455	-0.87275693402305
C	3.90154229272087	-0.97712693092482	0.70570142461195
H	1.86478098062431	-1.35865232635072	1.14477576579878
C	4.34651949430275	0.87168173326291	-0.77147574274363
H	2.65747833974726	1.91941292727807	-1.50358778692778
C	4.84840902646074	-0.18339982584525	0.02266215341488
H	4.22561474278063	-1.79405120386656	1.33179060459905
H	5.02344137477568	1.50938136910583	-1.31889446867264
O	-4.32967017984688	1.89119188365110	-1.05771766575096
O	-6.94458689989394	-1.31898620663947	0.83583576736965
N	-5.62147637509232	0.29136698968426	-0.11831794714727
H	-6.44641578429462	0.79515468373149	-0.41938377739484
N	-4.67099626598594	-1.49271866903727	0.95011895197822
H	-4.73832275404371	-2.35682849322033	1.46563406572926
N	-3.42296748096420	-1.06442971866008	0.69784666800345
N	6.19167633861114	-0.42579595688089	0.12394782728736
C	7.13988865882674	0.41606752082748	-0.57865929766998
H	7.07121679344121	1.46011225410459	-0.25632361036175
H	8.14678043105541	0.06391650226114	-0.37361160935708
H	6.98229678582188	0.38554950502801	-1.66132948367528
C	6.67585406595367	-1.51205769822840	0.95285287377884
H	6.28833993744315	-2.47901587054145	0.61718031942352
H	7.76019454735634	-1.54431032390115	0.89797297123395
H	6.39087424619327	-1.37965914185398	2.00168670966467

S_0 (DAU_{anti})

C	0.08896009601489	1.76195792397250	-1.08957090092322
C	0.37991599221254	0.55376773661427	-0.48377316919435
C	-0.75866441926947	-0.13764061735802	-0.04154830484007
C	-1.94158793458955	0.52673527820588	-0.29699692545166
S	-1.62161094219610	2.04040705934103	-1.09370658648793
H	1.38426921357396	0.16952032200012	-0.38381690349957
H	-0.73138044791122	-1.10229385687388	0.44274829828328
C	-3.26986439627688	0.05539409768447	0.03497907320068
C	-4.46284593861994	0.87067264239199	-0.30935343358849
C	-5.81811073425320	-0.92177992720875	0.69962167036398
C	1.01248352015540	2.73183889558160	-1.65968329655439
C	0.59368794371707	3.71491670237608	-2.56653630589375
C	2.37241955769557	2.72434960628576	-1.31889501536090
C	1.46964444599013	4.63845645902984	-3.10239785200819
H	-0.44337309464354	3.75204100989963	-2.87601380315429
C	3.26396327161855	3.63759668543551	-1.84711845147191
H	2.74154332658263	1.99652817223847	-0.60842816869248
C	2.83867632744593	4.62925634348531	-2.75813403058649
H	1.08861148346797	5.36682365229882	-3.80146969408489
H	4.29774085811554	3.58728254564474	-1.54132491732451
O	-4.42469328184848	1.95493634106059	-0.85796935036269
O	-6.89905693228833	-1.38606196647835	1.00772028239514
N	-5.66007583048227	0.29083518688417	0.05563057873823
H	-6.50690451854519	0.80365895360764	-0.15626370336846
N	-4.62154552350022	-1.53981066171085	0.95181491824707
H	-4.64830497376465	-2.43190800905779	1.42141875422813

N	-3.39399577976983	-1.08773785417360	0.63905225121400
N	3.71453990120962	5.54254392040646	-3.28299230924889
C	5.11836767825007	5.49525327815993	-2.92645226228618
H	5.57508338227266	4.54027525129906	-3.20579269068805
H	5.64453351359461	6.28804242055368	-3.45049307263147
H	5.26847534996706	5.63953289462873	-1.85159110792295
C	3.24375043176208	6.55522068451960	-4.20691448688501
H	2.47338307118331	7.18711475136214	-3.75390176964980
H	4.07717152367669	7.19059235247103	-4.49284344855937
H	2.82470385945262	6.11209172542201	-5.11638386595037

4.3. Excited state geometries in 1,4-dioxane.

S_1 for $DATU_{syn}$

C	-1.17453613077956	0.58544350831700	-0.29014841989801
C	-0.56252692622415	1.80063948549427	-0.61517861963707
C	0.82941461463460	1.73612969286119	-0.61117879232924
C	1.31569250653299	0.48108654822963	-0.28632455770155
S	0.04183111938442	-0.62766574660368	0.00585236769079
H	-1.11622295410768	2.70041372770742	-0.83629194617405
H	1.48380221143186	2.56823592851309	-0.82209171390898
C	2.72611310180871	0.04443433534867	-0.20122953301079
C	3.40042451739592	0.16684925903912	1.00911950655544
C	5.32778523528826	-0.81108287286651	-0.12883280466264
C	-2.56565376393620	0.30549104735348	-0.17444058391230
C	-3.03842298462158	-0.97228381929444	0.22017133756842
C	-3.54248356554358	1.30020797316356	-0.44049703883480
C	-4.37088278681236	-1.24133391510670	0.34813697367530
H	-2.32854544537553	-1.75936220150267	0.43497812355853

C	-4.87886793460459	1.04580858379820	-0.32144169265141
H	-3.22830777632482	2.28721029294253	-0.74638548544695
C	-5.34624372847575	-0.23812520483235	0.08238040844931
H	-4.67747299331072	-2.22798719250466	0.65663148365651
H	-5.58187193259497	1.83509238347901	-0.53537323737139
N	4.73952127752788	-0.28723890813733	0.97270619838559
H	5.28772706270414	-0.22231499557589	1.81581908092111
N	4.54156565773876	-0.87111687261170	-1.22097103536720
H	4.94363397957569	-1.25411644041354	-2.06004381278951
N	3.22700160560708	-0.46062391869520	-1.33628018765064
N	-6.66262941485811	-0.49426849784836	0.21012736746360
C	-7.65528865800105	0.53795037306485	-0.07347877594171
H	-7.57656174370298	0.87847577809709	-1.10732220601917
H	-8.64586096800061	0.12435178329754	0.08081439064942
H	-7.52680494829627	1.39440220140206	0.59075203652547
C	-7.12873969429748	-1.80940833909018	0.64039581836946
H	-6.73882952096479	-2.05452641011829	1.62971442325732
H	-8.21219625140509	-1.80002930876026	0.68679609196010
H	-6.81567543220476	-2.58249551819414	-0.06339150708338
S	6.91937147026604	-1.35164517497555	-0.14708255530687
S	2.77520119454648	0.79287243502277	2.45873889701128

S_2 for DATU$_{syn}$

C	-1.16832505059388	0.64681585966630	0.07653641358986
C	-0.56272301242676	1.88443818735153	0.13024851005215
C	0.84614470354476	1.82509340575661	0.09801129359785
C	1.34553845260757	0.54428702170404	0.02771981547251
S	0.03816990252985	-0.60542816151936	-0.00554766674504

H	-1.10873840307678	2.81420597714675	0.18545621205219
H	1.45984359843222	2.71604019578816	0.12264549607611
C	2.70419683028480	0.05158162652319	-0.03268995609622
C	3.83117063270612	0.88341555876358	0.00845321376098
C	5.24861596875569	-1.08437396718170	-0.13470157052338
C	-2.58855975017657	0.32871451106981	0.06843757880479
C	-3.05616233325773	-0.99144044766068	0.01058186047687
C	-3.56049509751609	1.33948735480151	0.10696868422046
C	-4.40367404662581	-1.29402656824937	-0.01379962693616
H	-2.35012581594410	-1.81244117349375	-0.01899974704494
C	-4.91194201985996	1.05714789752304	0.08444124210945
H	-3.25670114208864	2.37688808124775	0.15226115872840
C	-5.37906778430339	-0.27435300529883	0.02013459565812
H	-4.69983749190848	-2.33087439794853	-0.06114534698345
H	-5.61190611201108	1.87836763073772	0.11366240798301
N	5.07342351706925	0.27612010939004	-0.05326879882440
H	5.91656179999745	0.83014357189437	-0.04153780984219
N	4.09861417497827	-1.77266903108156	-0.16433549842041
H	4.16078844876698	-2.77801921859440	-0.22510198842948
N	2.83040376038678	-1.28530833472621	-0.12169846004108
N	-6.71823247589072	-0.56219453990883	-0.00918627407361
C	-7.69475443936840	0.50879294433219	0.01299656038927
H	-7.58793794693716	1.17464220642586	-0.85032586960702
H	-8.69279624443998	0.07994199713028	-0.01153190232796
H	-7.60750092196284	1.11572982351111	0.92000600917806
C	-7.16549163220653	-1.93754982415107	-0.10752394740414
H	-6.82859068913234	-2.53453269422683	0.74625824804801

H	-8.25168598067727	-1.95732649692578	-0.12611429101188
H	-6.79941493768910	-2.41857894002630	-1.02100298697204
S	6.76462576975627	-1.77712283146183	-0.19349283615908
S	3.87102576827775	2.60385567169120	0.13233527724438

T_1 for DATU$_{syn}$

C	-1.17121000802214	0.93143106335984	-0.96046665354980
C	-0.59395715533172	2.01385515417011	-0.27804817231728
C	0.79122641160241	2.00075749521212	-0.26409072110931
C	1.34295911195922	0.90161628457924	-0.94013000069634
S	0.07565190257441	-0.11186925437818	-1.59082320975123
H	-1.17723507333516	2.78939792878113	0.19655635775465
H	1.41232697223797	2.74140153480140	0.21054056718287
C	2.69377167136648	0.49575278483368	-1.15709440250203
C	3.84359471414152	1.17179675160141	-0.68394896524369
C	5.18424943378831	-0.57824852871993	-1.76542027090414
C	-2.55864424629105	0.65364871190626	-1.15118757528166
C	-3.00251727626697	-0.49384343383006	-1.84986313163014
C	-3.56096965838919	1.51165971235895	-0.63778723077774
C	-4.33419645331390	-0.77083952459645	-2.02455705978302
H	-2.27553334709046	-1.18254623288152	-2.26145195035771
C	-4.89694306146580	1.24778798812743	-0.80273202335058
H	-3.27615353812388	2.40247022746422	-0.09529285347696
C	-5.33452358666883	0.09212943336350	-1.50343027200187
H	-4.61304435908780	-1.66160361051107	-2.56629023740848
H	-5.61749371689620	1.93614946485104	-0.38812249238652
N	5.05814486212050	0.55636015671663	-1.04343948589225
H	5.90382679377506	1.00502725691671	-0.72461536001778

N	4.00357295256411	-1.12310060897076	-2.15322680112344
H	4.04447008928190	-1.97218466402430	-2.69600288729750
N	2.76693790084502	-0.65828795633499	-1.89778773471490
N	-6.65408295554804	-0.17801641195445	-1.66664483176751
C	-7.66475696361945	0.71776853725717	-1.12249333701860
H	-7.57683592546079	1.72054399269012	-1.54921667040642
H	-8.64909724042176	0.32802432358761	-1.36333621762030
H	-7.58066659132736	0.79606567277979	-0.03508203031623
C	-7.08052880891538	-1.36952523012807	-2.38713833080490
H	-6.71110427258579	-2.27798909224043	-1.90390533768049
H	-8.16557684336368	-1.40531939741681	-2.40029711428629
H	-6.72246426761742	-1.35735944675223	-3.42022081406209
S	6.64768466664345	-1.27460650660752	-2.16169075583683
S	3.96547786624256	2.57229542398837	0.23239800643662

S_1 for DAU_{syn}

C	0.60919964156641	0.53395923785111	-0.30202718412272
C	0.06079641649577	1.68563506042830	-0.82135918803904
C	-1.34164097856311	1.61156036713438	-1.00827883410715
C	-1.86299429575842	0.39423317014076	-0.63613705365389
S	-0.62678852520863	-0.66545276883215	-0.04083888584183
H	0.64359194461032	2.56841324410547	-1.04614115903718
H	-1.95989066284379	2.40868557210867	-1.38763884727916
C	-3.25266565692746	-0.09120185294189	-0.66084417790462
C	-4.34398015752748	0.81151813297134	-0.80621917711487
C	-5.81676134812968	-1.17167042091693	-0.66488671026893
C	1.99788912215298	0.26787098623996	0.01871069852852
C	2.37397571511794	-0.74634086010126	0.92381447750667

C	3.03514554815314	1.01158893694290	-0.58400625718113
C	3.69182828228299	-1.00811406634811	1.21183084871400
H	1.60702436638437	-1.32974805232672	1.41607468352557
C	4.35627211259463	0.77314005762733	-0.29769902715489
H	2.78609688259599	1.77377690316971	-1.30937269566563
C	4.72963659607237	-0.24926614598079	0.61379338175060
H	3.92673802123601	-1.78971523136384	1.91789528339416
H	5.11272610486913	1.35986841695822	-0.79588760003833
O	-4.29186604282789	2.04638032457649	-0.94093691073559
O	-6.91734731619560	-1.70919992545689	-0.66704005782822
N	-5.61497408925840	0.17374178702905	-0.80288783347812
H	-6.42887930271662	0.75869154812042	-0.91831008716683
N	-4.65151764344578	-1.88869899399663	-0.52615690237819
H	-4.74144721781818	-2.88807378569843	-0.43575903389625
N	-3.38254718782530	-1.40843289512915	-0.51674028811352
N	6.03638669104272	-0.49432494443627	0.89879164211827
C	7.08895936722159	0.33853759302605	0.33554608052638
H	6.91342566555152	1.39224296831802	0.56182112082227
H	8.04040703777544	0.04563240773700	0.76763818360083
H	7.14663875826390	0.22122859764840	-0.75037481588244
C	6.41131667899187	-1.59478208755759	1.77427108048953
H	5.99378701136298	-2.53716515006243	1.41370737040940
H	7.49312245866096	-1.67930751242678	1.79202024732940
H	6.05608600204318	-1.42521061855775	2.79476762817293

References

1. Lakowicz, J. R. Instrumentation for Fluorescence Spectroscopy. In *Principles of Fluorescence Spectroscopy*; Springer US: Boston, MA, **2006**, pp 27−61.
2. Neese, F. Software Update: The ORCA Program System—Version 5.0. *WIREs Comput. Mol. Sci.* **2022**, *12* (5), e1606.
3. Becke, A. D. A New Mixing of Hartree–Fock and Local Density-Functional Theories. *J. Chem. Phys.* **1993**, *98* (2), 1372–1377.
4. Tsuzuki, S.; Uchimaru, T. Accuracy of Intermolecular Interaction Energies, Particularly Those of Hetero-Atom Containing Molecules Obtained by DFT Calculations with Grimme's D2, D3 and D3BJ Dispersion Corrections. *Phys. Chem. Chem. Phys.* **2020**, *22* (39), 22508–22519.
5. Weigend, F.; Ahlrichs, R. Balanced Basis Sets of Split Valence, Triple Zeta Valence and Quadruple Zeta Valence Quality for H to Rn: Design and Assessment of Accuracy. *Phys. Chem. Chem. Phys.* **2005**, *7* (18), 3297.
6. Barone, V.; Cossi, M.; Tomasi, J. A New Definition of Cavities for the Computation of Solvation Free Energies by the Polarizable Continuum Model. *J. Chem. Phys.* **1997**, *107* (8), 3210–3221.
7. de Souza, B.; Farias, G.; Neese, F.; Izsák, R. Predicting Phosphorescence Rates of Light Organic Molecules Using Time-Dependent Density Functional Theory and the Path Integral Approach to Dynamics. *J. Chem. Theory Comput.* **2019**, *15* (3), 1896–1904.
8. Allouche, A. Gabedit—A Graphical User Interface for Computational Chemistry Softwares. *J. Comput. Chem.* **2011**, *32* (1), 174–182.
9. Neese, F.; Wennmohs, F.; Hansen, A.; Becker, U. Efficient, Approximate and Parallel Hartree–Fock and Hybrid DFT Calculations. A 'Chain-of-Spheres' Algorithm for the Hartree–Fock Exchange. *Chem. Phys.* **2009**, *356* (1–3), 98–109.
10. Brister, M. M.; Crespo-Hernández, C. E. Excited-State Dynamics in the RNA Nucleotide Uridine 5′-Monophosphate Investigated Using Femtosecond Broadband Transient Absorption Spectroscopy. *J. Phys. Chem. Lett.* **2019**, *10* (9), 2156–2161.
11. Reichardt, C.; Vogt, R. A.; Crespo-Hernández, C. E. On the Origin of Ultrafast Nonradiative Transitions in Nitro-Polycyclic Aromatic Hydrocarbons: Excited-State Dynamics in 1-Nitronaphthalene. *J. Chem. Phys.* **2009**, *131* (22), 224518.
12. Snellenburg, J. J.; Laptenok, S. P.; Seger, R.; Mullen, K. M.; Stokkum, I. H. M. van. Glotaran: A *Java* -Based Graphical User Interface for the *R* Package TIMP. *J. Stat. Softw.* **2012**, *49* (3), 1.

13. Oulianov, D. A.; Tomov, I. V.; Dvornikov, A. S.; Rentzepis, P. M. Observations on the Measurement of Two-Photon Absorption Cross-Section. *Opt. Commun.* **2001**, *191*, 235-243.

14. de Reguardati, S.; Pahapill, J.; Mikhailov, A.; Stepanenko, Y.; Rebane, A. High-Accuracy Reference Standards for Two-Photon Absorption in the 680–1050 nm Wavelength Range. *Opt. Express* **2016**, *24* (8), 9053-9066.

15. Spiller, W.; Kliesch, H.; Wöhrle, D.; Hackbarth, S.; Röder, B.; Schnurpfeil, G. Singlet Oxygen Quantum Yields of Different Photo-Sensitizers in Polar Solvents and Micellar Solutions. *J. Porphyr. Phthalocyanines* **1998**, *2* (2), 145–158.

16. Zhou, Z.; Liu, J.; Huang, J.; Rees, T. W.; Wang, Y.; Wang, H.; Li, X.; Chao, H.; Stang, P. J. A Self-Assembled Ru–Pt Metallacage as a Lysosome-Targeting Photosensitizer for 2-Photon Photodynamic Therapy. *Proc. Natl. Acad. Sci. U. S. A.* **2019**, *116* (41), 20296–20302.

17. Lin, X.; Chen, F.; Yu, X.; Wang, H.; Qiu, H.; Li, Y.; Yin, S.; Stang, P. J. Phenylthiol-BODIPY-Based Supramolecular Metallacycles for Synergistic Tumor Chemo-Photodynamic Therapy. *Proc. Natl. Acad. Sci. U. S. A.* **2022**, *119* (29), No. e2203994119.

18. Nguyen, V. N.; Qi, S.; Kim, S.; Kwon, N.; Kim, G.; Yim, Y.; Park, S.; Yoon, J. An Emerging Molecular Design Approach to Heavy-Atom-Free Photosensitizers for Enhanced Photodynamic Therapy under Hypoxia. *J. Am. Chem. Soc.* **2019**, *141* (41), 16243–16248.

19. Redmond, R. W.; Gamlin, J. N. A Compilation of Singlet Oxygen Yields from Biologically Relevant Molecules. *Photochem. Photobiol.* **1999**, *70* (4), 391–475.

20. Wilkinson, F.; Helman, W. P.; Ross, A. B. Quantum Yields for the Photosensitized Formation of the Lowest Electronically Excited Singlet State of Molecular Oxygen in Solution. *J. Phys. Chem. Ref. Data* **1993**, *22* (1), 113–262.

21. Acquah, C.; Pabis, Z.; Seth, S. K.; Levi, L.; Crespo-Hernández, C. E. Low-cost, 3D Printed Irradiation System for in Vitro Photodynamic Therapy Experiments. *Photochem. Photobiol.* **2024**, *100* (3), 530–540.

22. Lall, N.; Henley-Smith, C. J.; De Canha, M. N.; Oosthuizen, C. B.; Berrington, D. Viability Reagent, PrestoBlue, in Comparison with Other Available Reagents, Utilized in Cytotoxicity and Antimicrobial Assays. *Int. J. Microbiol.* **2013**, *2013*, 1–5.

23. Hopkins, P. A.; Sinkeldam, R. W.; Tor, Y. Visibly Emissive and Responsive Extended 6-Aza-Uridines. *Org. Lett.* **2014**, *16* (20), 5290–5293.

24. Seth, S. K.; Acquah, C.; Levi, L.; Jockusch, S.; Crespo-Hernández, C. E. Harnessing the Excited States of 5-(5-Phenylthiophen-2-yl)-6-Azauridine as a Three-Pronged Agent for Skin Cancer Therapy: Photodynamic Action, Cell Imaging, and Cancer Cell Inhibition. *ACS Appl. Bio. Mater.* **2025**, *8* (8), 7357–7369.

25. Seth, S. K.; Purkayastha, P. Unusually Large Singlet Oxygen ($^1O_2$) Production by Very Weakly Emissive Pyrene-Functionalized Iridium(III) Complex: Interplay between Excited ^{3}ILCT/^{3}IL and ^{3}MLCT States. *Eur. J. Inorg. Chem.* **2020**, *2020* (31), 2990–2997.

26. Adler, A. D.; Longo, F. R.; Finarelli, J. D.; Goldmacher, J.; Assour, J.; Korsakoff, L. A Simplified Synthesis for Meso-Tetraphenylporphine. *J. Org. Chem.* **1967**, *32* (2), 476–476.
